# Traditional Chinese Medicine for Post-stroke Sleep Disorders: The Evidence Mapping of Clinical Studies

**DOI:** 10.3389/fpsyt.2022.865630

**Published:** 2022-06-15

**Authors:** Qing Su, Danmei Zou, Nuo Gai, Huishan Li, Zhuoran Kuang, Xiaojia Ni

**Affiliations:** ^1^The Second Clinical School of Guangzhou University of Chinese Medicine, Guangzhou, China; ^2^Guangdong Provincial Hospital of Chinese Medicine, Guangdong Provincial Academy of Chinese Medical Sciences, Guangzhou, China

**Keywords:** complementary therapy, stroke, sleep disorders, evidence map, traditional Chinese medicine

## Abstract

**Background and Purpose:**

Recently, there are a number of clinical studies on traditional Chinese medicine (TCM) for post-stroke sleep disorders (PSSDs). This study aimed to map the current clinical studies and identify gaps to inform future study agendas.

**Methods:**

PubMed, Embase, Cochrane Library, and Chinese databases, including SinoMed, CNKI, and Wanfang, were searched for clinical studies on PSSDs treated with TCM from their inception to September 2021. Evidence sources, number of studies, types of PSSDs, intervention categories, effectiveness, and quality assessment were graphically displayed.

**Results:**

The evidence map involved 810 clinical studies, of which the earliest report was dated back to 1993, and an advanced growth of the whole evidence was observed in 2012. Randomized controlled trials (RCTs) were the most common type of study design (78.15%), and post-stroke insomnia was the most common type of sleep disorders (65.80%). The benefits of Chinese herbal medicine (CHM) and acupuncture therapies for post-stroke insomnia have been widely reported in RCTs (81.60% and 75.38%, respectively). However, the benefits of CHM interventions were assessed using a global approach rather than being based on a specific formula, and the highest level of evidence supporting the effectiveness of acupuncture therapies was of low methodological quality. In addition, evidence from primary studies was insufficient in the areas of TCM for post-stroke sleep-related breathing disorders (SBDs) and Chinese mind-body exercises for post-stroke insomnia.

**Conclusions:**

PSSDs treated with TCM have been widely assessed in clinical studies. For better evidence translation, clinical trials on specific CHM interventions and high-quality systematic reviews on acupuncture for post-stroke insomnia should be conducted. For a better solution to clinical questions, TCM on SBDs after stroke and the benefits of Chinese mind-body exercises for post-stroke insomnia should be explored in future clinical studies.

## Introduction

Stroke leads to mortality and disability worldwide, and the number of stroke survivors has been continuously increasing (1). Sleep disorders are prevalent after stroke (e.g., up to 69.2% for post-stroke insomnia, and up to 72% for sleep apnea after stroke) (2, 3), in either the acute or rehabilitation phase (4). Its cause, persistence, and progression are often considered multifaceted, including stroke-related brain impairments and environmental, psychological, and other factors (5, 6). Increasing evidence suggests that sleep disorders are associated with an increased risk of recurrent stroke and poor prognosis in stroke patients (7–10). Conversely, some evidences have shown that good sleep improves neuroplasticity and functional recovery after stroke (11, 12).

However, current treatments for post-stroke sleep disorders (PSSDs) are unsatisfactory. For example, although cognitive-behavioral therapy for insomnia (CBT-i) is the first-line recommendation for insomnia (13, 14), its application in post-stroke insomnia is challenging. First, the evidence of CBT-i for post-stroke insomnia was limited; second, patients might not comply well with the CBT-i program due to feasibility problems, such as cognitive impairment and physical disability after stroke (15, 16). Pharmacotherapy, such as benzodiazepine drugs, is an alternative treatment for insomnia (13) and has also been used to improve sleep in post-stroke patients. However, adverse events, such as hangover symptoms, drug tolerance and dependence, and increased risk of falls, have been considerable (17, 18). In addition, the uncertain risks of multi-drug interactions between stroke medications and sedative-hypnotics may increase (19). Finally, the clearance rate of sedative-hypnotics drugs declined as most stroke patients were at the middle-to-old age, which might amplify the adverse effects of pharmacological agents (20–22). Another example is the infeasibility of using continuous positive airway pressure (CPAP) for breathing-related sleep disorders after stroke (23, 24). Stroke patients might not comply with CPAP because stroke-related impairments, such as facial paralysis and dysphasia, often result in mask leakage and risk of aspiration with positive pressure (25, 26). Thus, there is a substantial need to develop effective, safe, and affordable treatment regimens for patients with stroke and sleep disorders.

Accumulated evidence has suggested that traditional and complementary therapies improved sleep (27, 28), such as Chinese herbal medicine (CHM), acupuncture and Taichi for insomnia (29–31), acupuncture for sleep-related breathing disorders (SBDs) (32), and acupuncture for restless leg syndrome (RLS) (33). There have been a number of clinical studies on traditional Chinese medicine (TCM) for PSSDs, suggesting that TCM might be a source of new therapies. However, an overview synthesizing all its clinical evidence has not been published.

An evidence map is an evidence synthesis tool that assists clinical investigators in developing future study agendas, which often includes a systematic search of a board field and identification gaps in knowledge and future study directions by visualizations of the evidence (34). In light of various sources of clinical evidence, we established an evidence map to primarily learn about the completeness of current evidence and the evidence gaps in TCM for PSSDs and to identify future study directions.

## Methods

We developed this evidence map based on a previous methodological study (35) and the *International Initiative for Impact Evaluation Evidence Gap Methodolog*y (3iE) (36). This study was reported in compliance with the *Preferred Reporting Items for Systematic Reviews and Meta-Analyses extension for scoping reviews* (37).

### Eligibility Criteria

In brief, this study included clinical studies on TCM for PSSDs. Studies that met all of the following criteria were eligible:

#### Participants

Participants who were diagnosed with PSSDs, including insomnia, SBDs, RLS, sleep-wake disorders, and daytime sleepiness.

#### Intervention

Participants who were treated with TCM, which included CHM; acupuncture therapies, such as needling, electro-acupuncture, acupressure, and acupoint moxibustion; other TCM therapies, such as Taichi and massage; and a combination of multiple TCM methods.

#### Study Designs

Any type of clinical studies as follows: systematic reviews (SRs), randomized clinical trials (RCTs), non-randomized controlled clinical trials (non-RCTs), cohort studies, case-control studies, case series, case reports, and personal views/opinions on treatments, and protocols of the study design described above as protocols were evidence in production.

There were no limitations in publication language, control group, and outcome measurements. Duplicates and studies without sufficient information to make a judgment on the participants, intervention, or study design were excluded.

### Information Source, Literature Search, and Quality Assessment

PubMed, Embase, Cochrane Library, and Chinese databases, including SinoMed, CNKI, and Wanfang, were searched for clinical studies on PSSDs treated with TCM from their inception to September 2021. The search strategy is shown in Supplementary Table 1. Two reviewers (QS and HL) screened the literature independently by removing duplicates, reviewing the titles and abstracts, and reading the full texts. If there was any uncertainty, a third reviewer (DZ) was consulted. As the preliminary search identified SRs, the highest level of evidence pyramid, the reviewers (QS, NG) only assessed the methodological quality of SRs using *A MeaSurement Tool to Assess systematic Reviews-2* (AMSTAR 2) (38).

### Data Extraction, Analysis, and Evidence Map Production

Two reviewers (QS and DZ) collected the data using a pre-designed spreadsheet, and the senior reviewer (ZK) validated the data. The data items included the basic characteristics of the studies, such as language, publication type, journal type and specialty, research collaboration, country, study design, disease, and type of intervention. Only the outcome data for RCTs and SRs were extracted, as they were considered the highest level of the evidence pyramid. Their results on the comparative effectiveness between TCM and control were summarized as potentially better, potentially worse, mixed, no difference, and unclear. The mixed result referred to the case that one therapy resulted differently per outcomes. Descriptive statistics were applied in the data analysis and evidence synthesis to gain an overview of existing evidence and identify study gaps. The evidence was mapped using area charts for the presentation of time-dependent changes; using sunburst charts to present the composition of studies in terms of diseases, interventions, and study designs; and using bubble graphs and pie charts for the results of SRs and RCTs, respectively. Microsoft Excel 2021 was used for statistical analysis and graph production.

## Results

### Selection of Sources of Evidence

**Figure 1:**
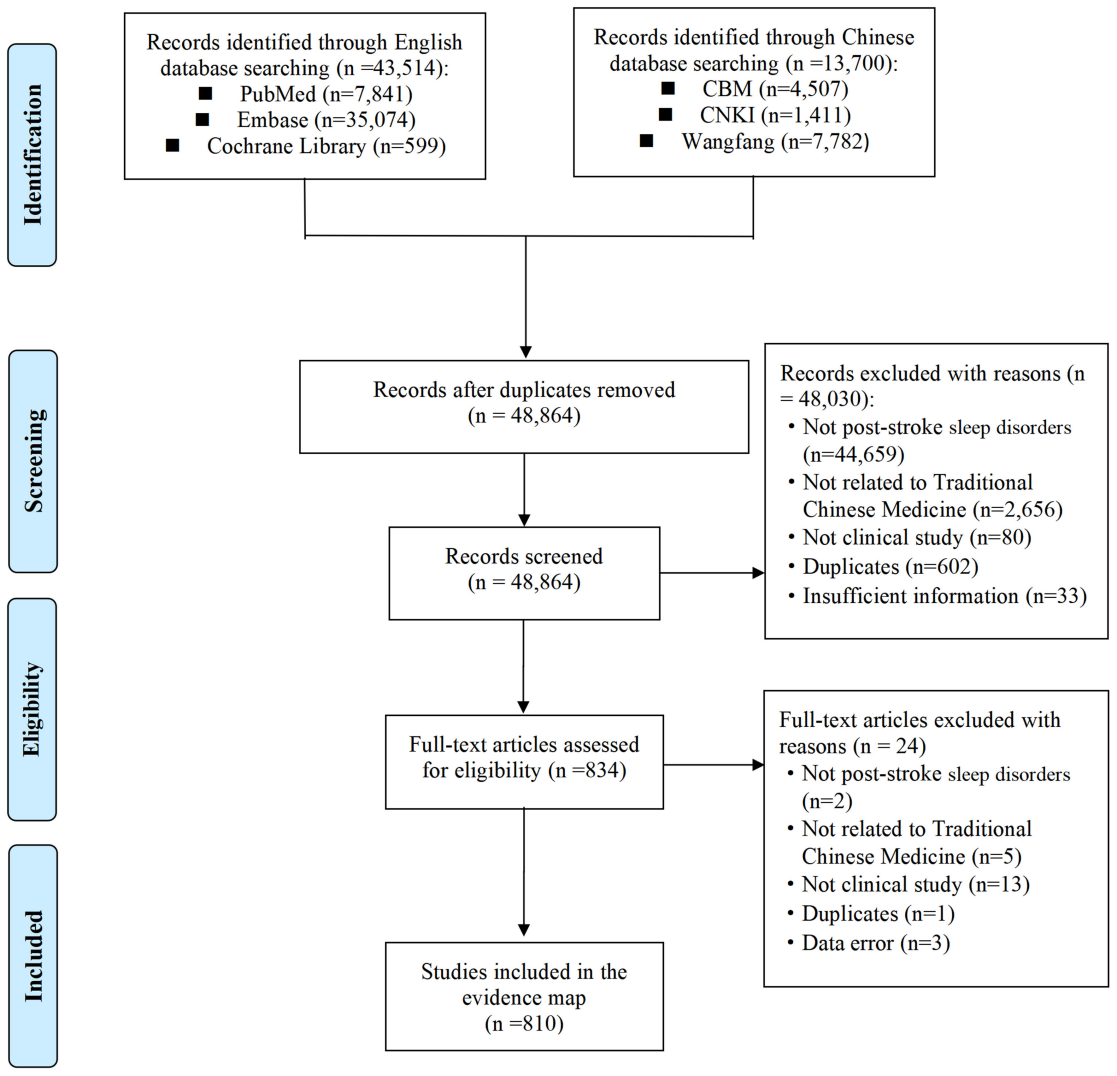
Flowchart of study identification.

The search initially identified 57,214 citations, and 810 eligible studies were finally included in this evidence map. The study identification and selection process are illustrated in Figure 1. The full list of included studies is shown in Supplementary Table 2.

### Basic Characteristics of Sources of Evidence

Most studies were written in Chinese (790, 97.53%), including 699 journal articles, 76 theses, and 15 conference papers. Among the few studies from English journals, most were Science Citation Indexed articles (*n* = 14, 70%). The included studies were widely published in journals within the scope of traditional, complementary, and alternative medicine (*n* = 410, 57.02%), and only 22 were published in journals specific to sleep medicine (3.06%). Studies in collaboration with other institutions (*n* = 106, 13.09%) or international partners (*n* = 3, 0.37%) accounted for minor modes of study implementation. Most of the studies were initiated by Chinese academics, whereas the others were conducted by Korean researchers (807 vs. 3). The basic characteristics of the sources of evidence are specified in Table 1.

**Table 1:** Basic characteristics of the source of evidence.

**Basic characteristics**	**Number (*n* = 810)**	**%**
**Language**		
Chinese	790	97.53
English	20	2.47
**Type of publication**		
Journal articles	719	88.77
Thesis	76	9.38
Conference paper	15	1.85
**Journal specialty** ^**a**^		
Sleep medicine	22	3.06
Traditional/complementary and alternative medicine	410	57.02
Other specialties	287	39.92
**Journal types** ^**b**^		
Chinese journal	699	97.22
Chinese scientific and technical papers and citation indexed^c^	49	7.01
English journal	20	2.78
Science Citation Indexed^d^	14	70
**Research collaboration**		
Single institution	701	86.54
Cross-institutions	106	13.09
International	3	0.37
**Country for primary corresponding author(s)**		
China	807	99.63
Korea	3	0.37

^a^ *The calculation of journal specialty was based on the total number of journal articles (n = 719).*

*^b^The calculation of journal types was based on the total number of journal articles (n = 719)*.

*^c^The calculation of Chinese scientific and technical papers and citations indexed was based on the total number of Chinese journals (n = 699)*.

*^d^The calculation of the science citation index was based on English journals (n = 20)*.

### The Overview of Evidence Composition

Regarding study design, the evidence body was composed of 21 SRs (2.59%), 633 RCTs (78.15%), 74 non-RCTs (9.14%), 1 cohort studies (0.12%), 1 case control (0.12%), 46 case series (5.68%), 24 case reports (2.96%), and 10 personal viewpoints on treatments (1.23%).

Regarding the type of intervention, 265 were for CHM (32.72%), 349 were for acupuncture therapies (43.09%), 22 were for other TCM therapies (2.72%), 172 were for a combination of multiple TCM methods (21.23%), and 2 did not specify the types of TCM therapies. Further details in terms of TCM interventions are shown in Supplementary Table 3.

**Figure 2:**
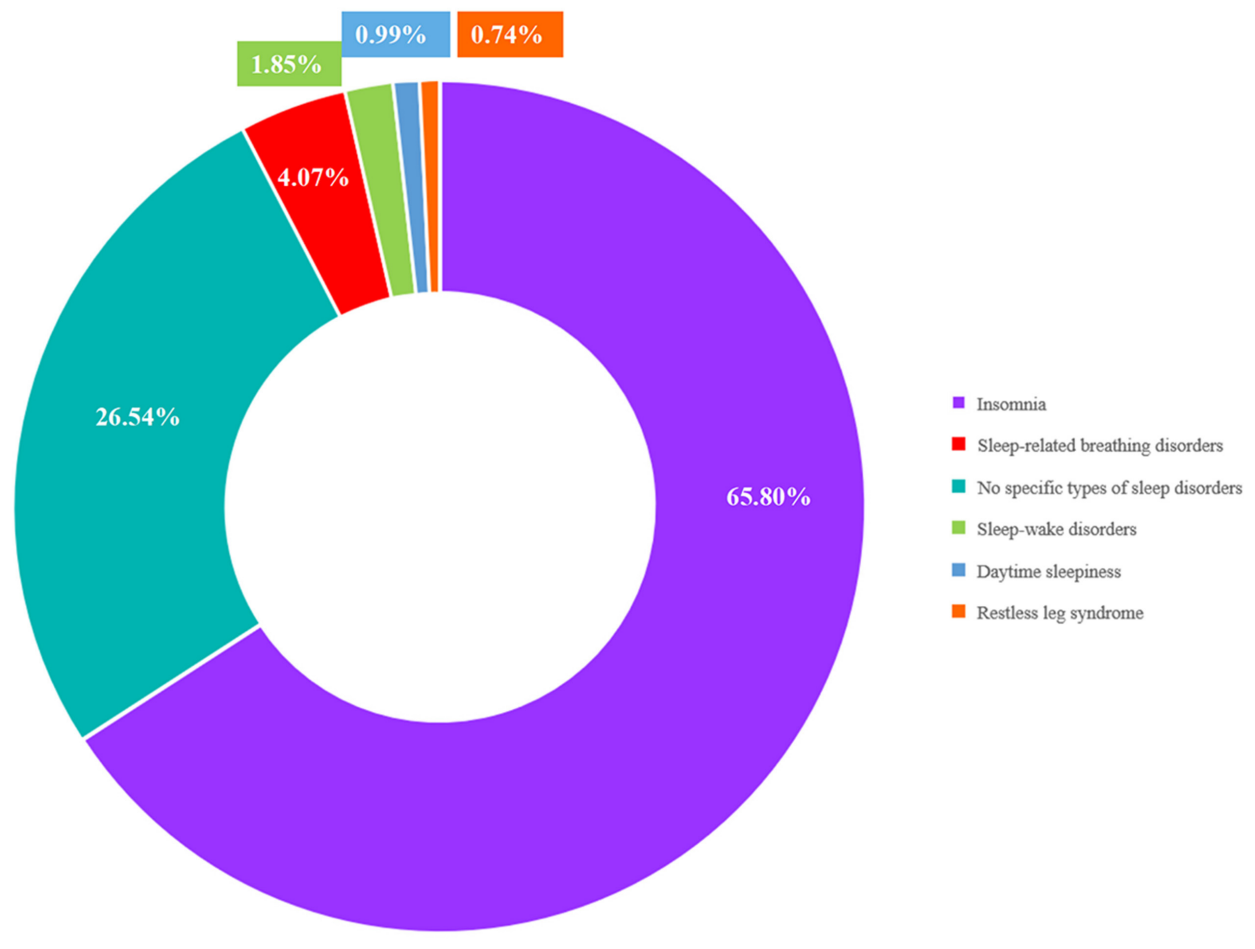
The overview of evidence body per sleep disorders.

With respect to the type of sleep disorder in post-stroke participants, 533 studies were for insomnia (65.80%), 33 were for SBDs (4.07%), and 29 were for other sleep disorders (3.58%), such as RLS, excessive sleepiness, and sleep-wake disorders. Unexpectedly, 215 studies did not specify the type of sleep disorders. Figure 2 shows the composition of the entire body of evidence in terms of the type of sleep disorders.

### The Time-Dependent Changes of Evidence

The first published study was traced back to 1993. Between 1993 and 2011, the growth rate was 5.21%, and the total number of studies was 99. From 2012 onward, the number of published studies increased, with the growth rate was 70.89% between 2012 and 2020. There was a peak in publications in 2020, of which 123 studies were published. A total of 73 studies were published between January and September in 2021.

**Figure 3:**
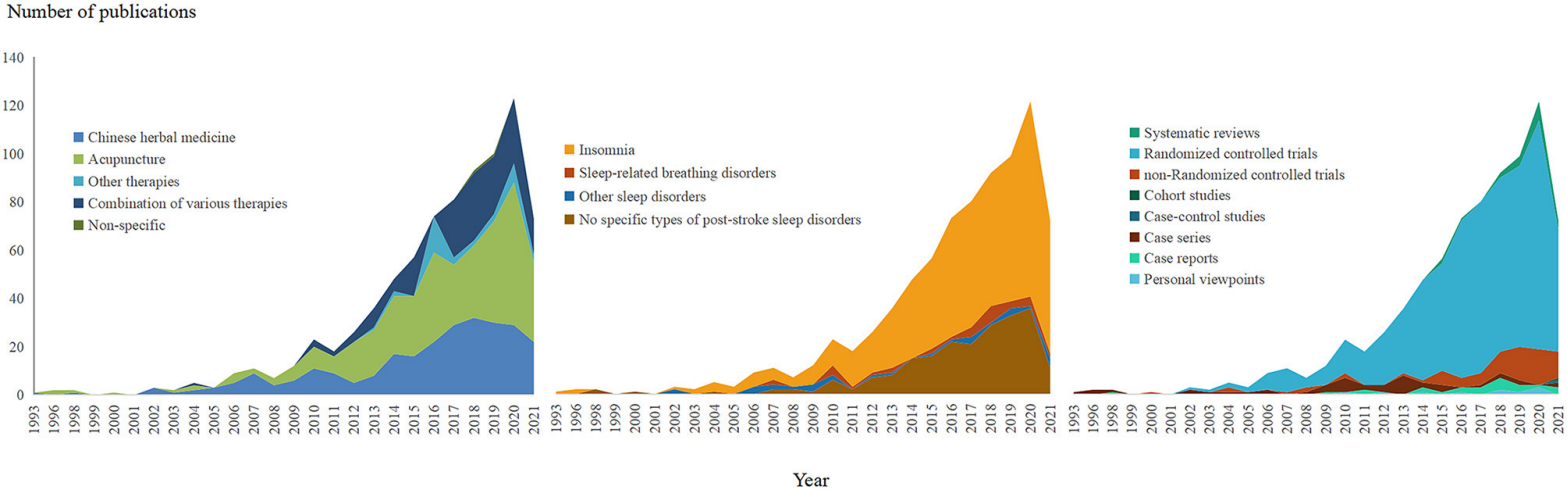
The time-dependent changes of publications on traditional Chinese medicine for post-stroke sleep disorders.

Regarding types of sleep disorders in stroke participants, insomnia was first studied in 1993, and publications of the same disease have increased steadily. The publication of SBDs began in 2007, which was about sleep apnea, and studies on other sleep disorders appeared in 2002, which was about sleep-wake disorder. Neither of these two types of sleep disorders evidently increased. Studies without specific types of sleep disorders were first published in 1998 and have been continuously published up to 2021. Regarding the types of intervention, CHM was first studied in 1993, followed by acupuncture in 1996, combination therapies in 2004, and other TCM therapies, such as massage, in 2013. Almost all of the studies on TCM therapies increased in number, and those for acupuncture had the greatest growth rate. Figure 3 shows the overall time-dependent changes in published studies on TCM for PSSDs in terms of study design, type of sleep disorder, and interventions.

### Study Designs and Types of Sleep Disorders and Interventions

#### Systematic Reviews

**Figure 4:**
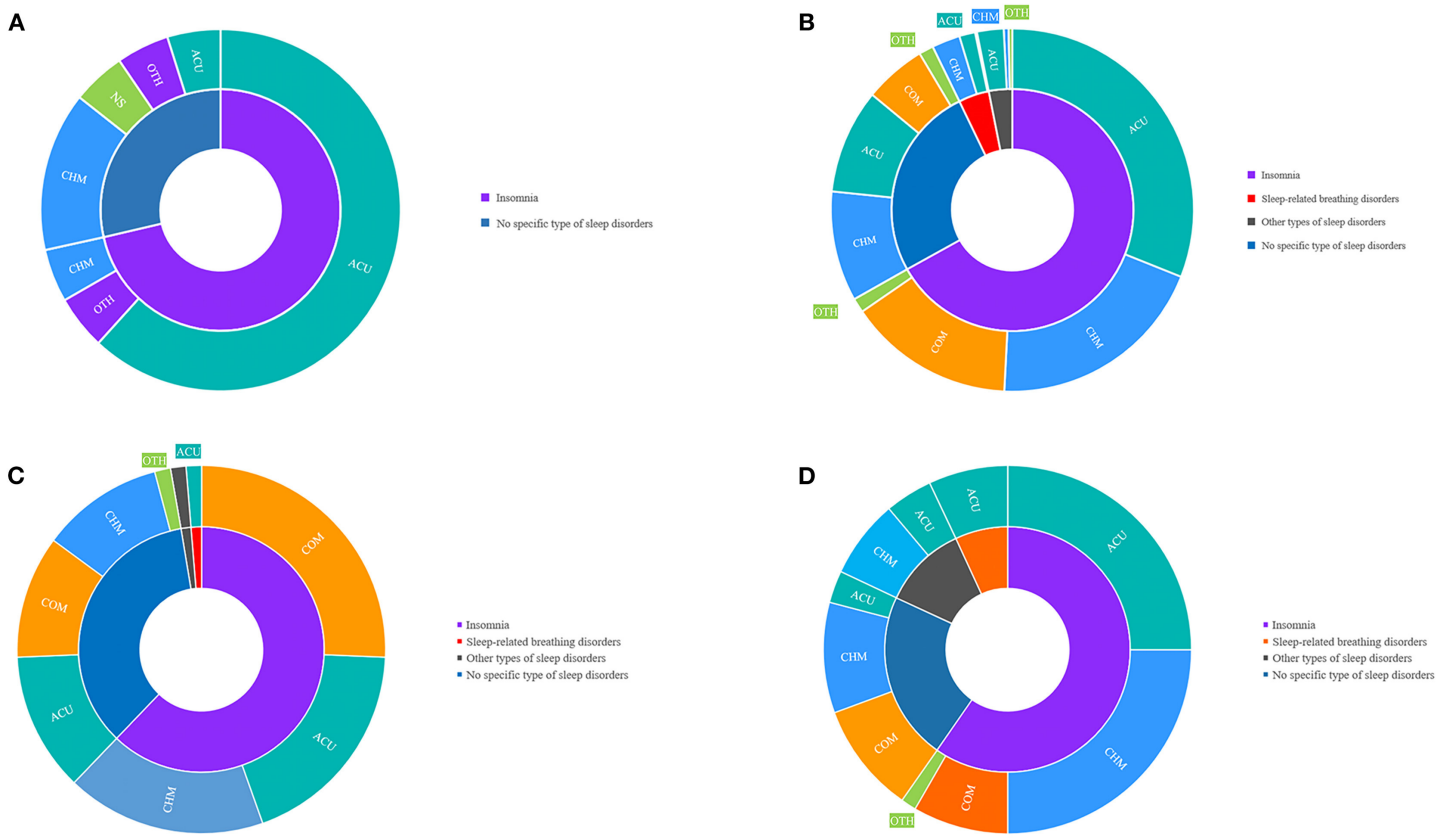
Evidence body per study designs and types of sleep disorders and interventions. **(A)** the composition of systematic reviews in terms of the type of sleep disorder and traditional Chinese medicine treatment; **(B)** the composition of the randomized controlled trials in terms of the type of sleep disorder and traditional Chinese medicine treatment; **(C)** the composition of non-randomized controlled trials in terms of type of sleep disorder and traditional Chinese medicine treatment; **(D)** the composition of the observational studies in terms of the type of sleep disorder and traditional Chinese medicine treatment. ACU, acupuncture therapies; CHM, Chinese herbal medicine; COM, a combination of multiple Traditional Chinese Medicine methods; OTH, other Traditional Chinese Medicine therapies.

Most SRs were related to post-stroke insomnia (*n* = 15, 71.43%), including one treated with CHM, 13 with acupuncture therapies, one with massage. The remaining SRs did not have specific types of sleep disorders in stroke patients (*n* = 6, 28.57%), including three for CHM and one lacking detailed TCM treatment. Almost all SRs considered TCM treatments as a whole, except for one study that focused on *Yangxue Qingnao* granule, a Chinese patent drug made of polyherbal ingredients. Figure 4A shows the composition of SRs in terms of the type of sleep disorder and TCM treatment.

#### Randomized Controlled Trials

Among the 633 RCTs, post-stroke insomnia was the most widely studied (*n* = 423, 66.82%), followed by the unspecified sleep disorder type (*n* = 164, 25.91%), SBDs (*n* = 26, 4.11%), and other sleep disorders (*n* = 20, 3.16%), including sleep-wake disorders (*n* = 11, 55%), daytime sleepiness (*n* = 6, 30%), and RLS (*n* = 3, 15%). Of the RCTs on insomnia, acupuncture therapies (*n* = 196, 46.34%) were investigated most frequently, such as needling acupuncture and electro-acupuncture (*n* = 107, *n* = 54.59%), auricular acupressure (*n* = 19, 9.69%), acupoint moxibustion (*n* = 9, 4.59%), and integration of multiple acupuncture therapies (*n* = 42, 21.43%). CHM was secondly investigated in RCTs for post-stroke insomnia (*n* = 125, 29.55%), of which Chinese herbal formulae, such as *Suanzaoren* decoction and *Chaihulonggumuli* decoction, and Chinese patent drugs, such as *Bailemian* capsule and *Yangxueqingnao* granule, were studied most (full ingredients are detailed in Supplementary Table 4). The combination of multiple TCM treatments was evaluated in 94 RCTs (22.22%), and other TCM therapies were assessed in eight RCTs (1.89%), for instance, four for massage (50%), three for TCM musical therapy (37.5%), and one for TCM exercise (12.5%). As for RCTs on SBDs, CHM was most commonly assessed (*n* = 16, 61.54%), followed by acupuncture therapies (*n* = 9, 34.62%), and combination therapy (*n* = 1, 3.85%). Figure 4B shows the composition of the RCTs in terms of the type of sleep disorder and TCM treatment.

#### Non-randomized Controlled Clinical Trials

Of the 74 non-RCTs, post-stroke insomnia continuously ranked at the top (*n* = 46, 62.16%), including 19 non-RCTs for combination therapies (41.30%), 13 for CHM (28.26%), and 14 for acupuncture therapies (30.43%), followed by unspecified sleep disorders (*n* = 26, 35.14%), and SBD (*n* = 1, 1.35%), which were treated with acupuncture therapies and sleep-wake disorders (*n* = 1, 1.35%). Figure 4C shows the composition of non-RCTs in terms of type of sleep disorder and TCM treatment.

#### Observational Studies

Among the 72 observational studies, post-stroke insomnia accounted for most of the type of disease (*n* = 43, 59.72%), followed by those without a specific type (*n* = 16, 22.22%); other types of sleep disorders (*n* = 8, 11.11%), such as sleep-wake disorders (*n* = 3), RLS (*n* = 3), and daytime sleepiness (*n* = 2); and SBDs (*n* = 5, 6.94%). Regarding the types of treatments, CHM was assessed most frequently in observational studies for post-stroke insomnia (*n* = 18, 41.86%), followed by acupuncture therapies (*n* = 18, 41.86%), combination therapies (*n* = 6, 13.95%), and other therapies, such as TCM exercise (*n* = 1, 2.33%). All five observational studies on SBD included acupuncture therapy. Figure 4D shows the composition of the observational studies in terms of the type of sleep disorder and TCM treatment.

#### Personal Viewpoints on Treatments

Of the 10 personal viewpoints on treatment, six were for insomnia, one for SBD, and three for sleep disorders of unspecific types. For types of interventions, CHM was discussed in four papers, and acupuncture were discussed in three papers, combination therapies were discussed in two papers, and TCM treatments without specific types were discussed in one paper.

### Evidence Synthesis for Therapeutic Effectiveness

**Figure 5:**
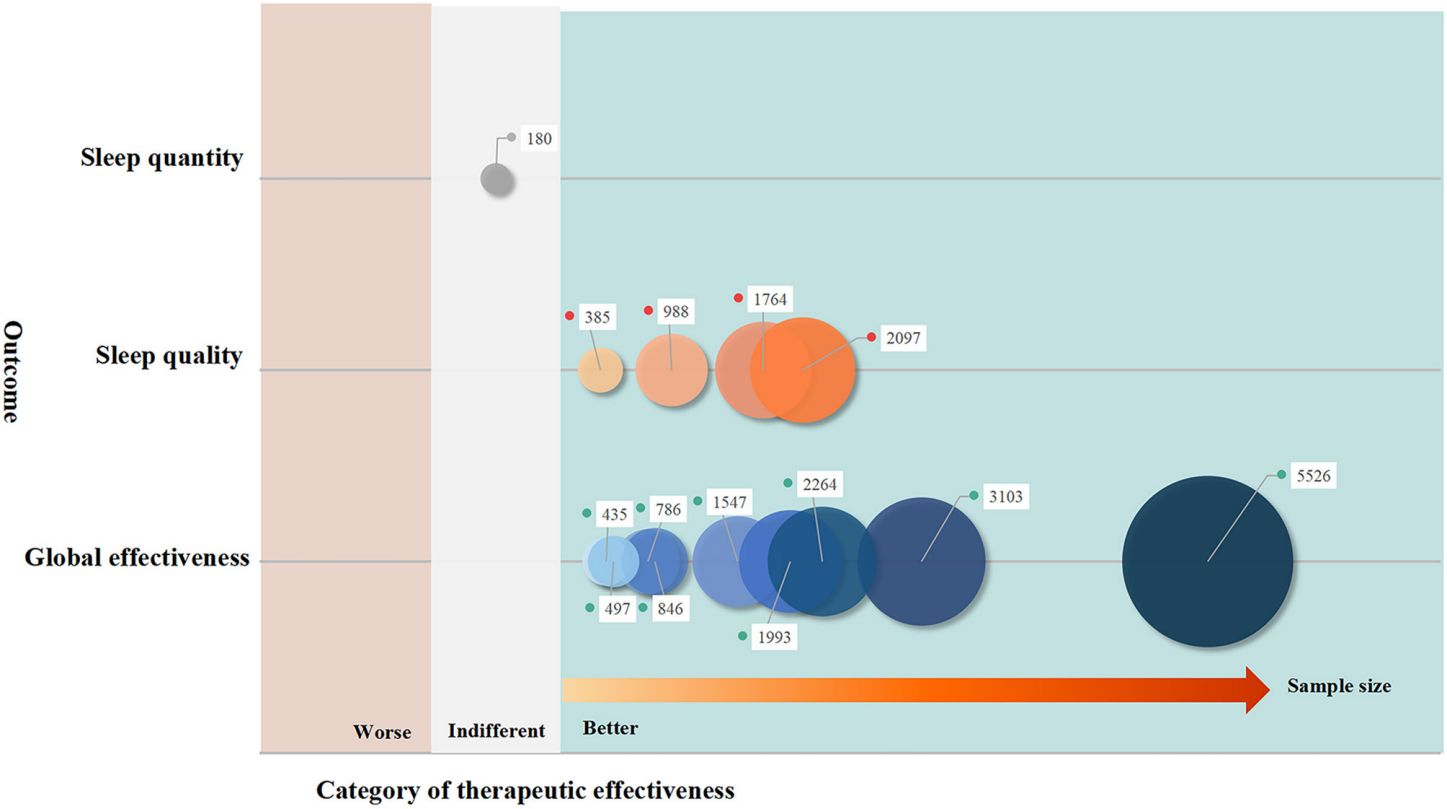
The effectiveness of traditional Chinese medicine on post-stroke sleep disorders: evidence synthesis of systematic reviews. The size of bubbles is decided by the sample size included to the pairwise comparison in terms of certain outcome measurements. Better: the meta-analysis favoring traditional Chinese medicine; worse: the meta-analysis favoring comparators; indifference: there is no statistical difference between traditional Chinese medicine and comparators in the meta-analysis.

Of the 21 SRs, 14 had reported therapeutic effectiveness. Three categories of results were reported, including TCM being potentially better than the control (*n* = 9, 64.29%), absence of statistical difference between TCM and the control (*n* = 2, 14.29%), and mixed results (*n* = 3, 21.43%). However, all SRs had low methodological quality in terms of the AMSTAR scores (Supplementary Table 5). After excluding five studies with unspecific types of PSSDs, the pool of nine SRs suggested that acupuncture showed potential benefits in improving subjective sleep quality, daytime functioning, and global effectiveness, whereas acupuncture did not improve sleep onset latency and total sleep duration (Figure 5).

**Figure 6:**
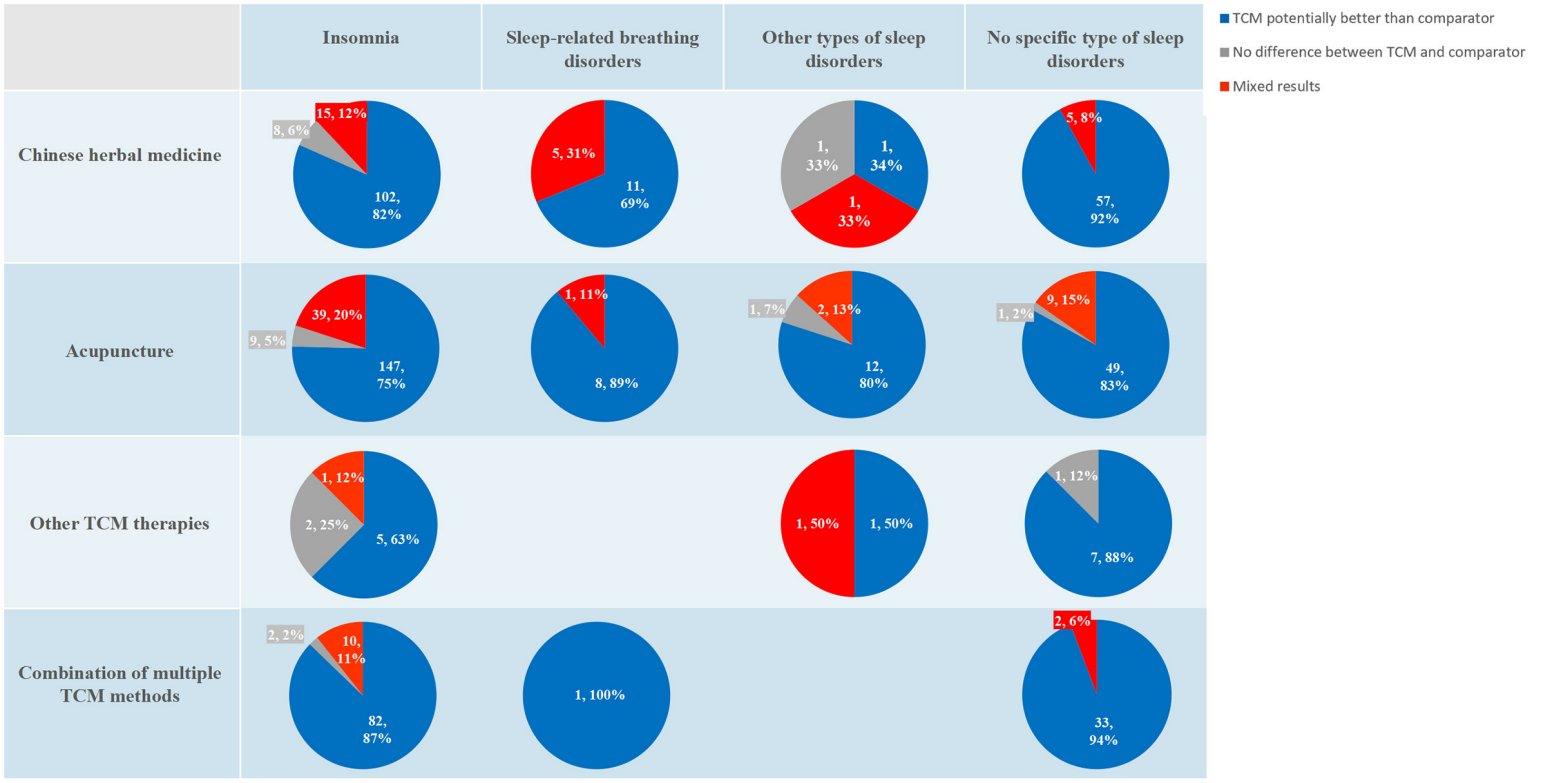
The effectiveness of traditional Chinese medicine on post-stroke sleep disorders: evidence synthesis of randomized controlled trials. The results are presented with the number of studies (%). TCM, traditional Chinese medicine.

As SRs were only for post-stroke insomnia and sleep disorders of unspecified types, the results from RCTs were further summarized to gain an overview of TCM effectiveness for all PSSDs. A total of 632 RCTs were included in the analysis after removing one without results. For post-stroke insomnia, most studies suggested that TCM performed potentially better than the control; however, a considerable number of RCTs on acupuncture therapies reported mixed results (*n* = 39). Regarding SBDs, the superiority of CHM or acupuncture therapies over the control was reported in most studies, and none reported indifference between TCM and the comparator. However, approximately one-third of CHM studies reported mixed results (*n* = 5). With respect to other sleep disorders, CHM was of varied effectiveness, whereas acupuncture therapies presented potential benefits compared to controls in most RCTs. For sleep disorders without specific types, the greatest number of RCTs with positive results was identified in those for CHM (*n* = 57). Figure 6 shows the overall therapeutic effectiveness of the synthesis of RCTs. In addition, we found that the mixed results were often due to various outcome measurements applied in RCTs, such as sleep quality, sleep quantity, neurological impairment, psychological wellbeing, quality of life, and TCM syndromes; therefore, we attached the Supplementary Table 6 to detail the pooled results of RCTs on TCM for PSSDs per outcome measurements.

## Discussion

### A Summary of Main Findings

This evidence map of 810 studies showed an overview of clinical evidence on TCM for PSSDs, and RCTs accounted for the majority. Evidence has been continuously growing, and sharp growth has been sustained in the past decade. Regarding the first appearance of different study designs on TCM for PSSDs, publishing the first study, a case series, was traced back to 1993; the first RCT was published in 2002, and the first SR was published in 2015. The major clinical evidence was the effectiveness assessment for CHM and acupuncture, and post-stroke insomnia was the type of participants in most studies. Although most RCTs supported the potential benefits of CHM and acupuncture therapies for PSSDs, mixed results of a few studies were observed for particular types of PSSDs and intervention. Most SRs suggested the superiority of TCM in improving PSSDs; however, most of them had low methodological quality. This study included various sources of clinical evidence, which was of strength in gaining a broad view of the current evidence and identifying the evidence gaps using simple descriptive statistics and the visualization of a large number of studies, compared with a classical SR and meta-analysis, which set rigorous eligibility criteria to answer a specific study question (34).

### Evidence Completeness and Explanations

This evidence map found that post-stroke insomnia was the most studied condition in clinical studies. This may be explained by the long history of TCM application in the treatment of insomnia (39, 40) and clinical evidence favoring the benefits of TCM in improving insomnia (41, 42). The interest and popularity of the public in seeking TCM to improve insomnia may be another reason (43). However, TCM for post-stroke SBDs has been studied in significantly fewer studies, which is inconsistent with the high prevalence of SBD in stroke patients (44). In addition, the negative impact of SBDs on the prognosis of stroke patients, such as neurological impairments, and long-term endpoints, such as recurrent stroke and mortality, have been identified, which is a priority in developing therapies to improving the outcome (45, 46). Thus, studies exploring the benefits of TCM for SBDs in patients with stroke are urgently required. The study also revealed that the evidence assessing TCM for other types of PSSDs, such as RLS, was limited. Further studies of this clinical question should be conducted, as it was historically documented that CHM was used for the treatment of RLS, which dated back to 1529 AD, approximately a century and a half earlier than the first report in England (47). In addition, high-quality studies supporting the benefits and safety of TCM for RLS have been continuously published (33, 47, 48).

This evidence map identified a large number of clinical studies on CHM and acupuncture therapies, whereas Chinese mind-body exercises, such as Taichi and Baduanjin, were rarely studied. The Chinese mind-body exercises promotes mind-body interaction by combining specific postures and movements with deep breathing and mental focus (49). Previous clinical evidence has shown that Taichi can improve sleep quality and reduce the severity of insomnia (31, 50). Clinical studies of Taichi could be extended to participants with post-stroke insomnia as its occurrence was also due to variable factors, possibly impaired sleep regulation after stroke, and environmental and psychological factors (2, 3).

Most evidence was from Chinese journals, which was not surprising as TCM originated from China, and it is widely accepted in modern China, particularly in elderly people (51). Almost all the evidence from English written journals was for acupuncture therapies, which could be partially explained by the greater interest in studying and applying acupuncture in the West compared to other TCM therapies (52). For example, the US National Center for Complementary and Alternative Medicine funded many acupuncture studies, which accounted for 64.52% of all CAM studies (53). In addition, the US Food and Drug Administration approved acupuncture needles as medical devices, and 1.4% of the US population used acupuncture early in 2008 (54). However, CHM is commonly considered a food supplement in Western countries, such as the USA, and the approval of its clinical use has been a great challenge (54). This may be the reason why clinical evidence from CHM, a major type of TCM therapy, is not commonly published in English.

Regarding the types of CHM used for post-stroke insomnia, the evidence map identified some Chinese patent drugs that were not commonly used for primary insomnia, such as *Yangxueqingnao* granule. Its main ingredients, *Radix Angelicae Sinensis, Rhizoma Chuanxiong*, and *Caulis Spatholobi*, have been shown to improve cerebral ischemia in laboratory experiments (55–57), which suggests that the selection of CHM for post-stroke insomnia is probably based on its pharmacological actions in ameliorating ischemic impairments. This differs greatly from the therapeutic principles of CHM for primary insomnia (43). Regarding the CHM formulae, both post-stroke insomnia and primary insomnia favored the *Suanzaoren* decoction. Although *the Suanzaoren* decoction has been assessed in many preclinical and clinical studies for primary insomnia (58–60), a large-scale RCT with a rigorous design is required to evaluate its efficacy in post-stroke insomnia.

Regarding the types of study design, the publication of SRs on TCM for PSSDs was more than 15 years after the first SR for TCM (61), and the study questions in most of the included SRs lacked focus and specificity. Although most SRs supported the positive effect of TCM on PSSDs, these results might overestimate the effect size due to the low quality of SRs (62). In addition, publication bias should be considered, as SRs with positive results are more likely published (63). Thus, more SRs with a specific study question and of high quality are required for PSSDs treated with TCM. This evidence map also found that an overwhelming number of RCTs on TCM for PSSD were published compared with observational studies of a significantly smaller quantity. Although the RCT was the golden standard for evaluating the treatment effect (64), it was at a high cost. A well-designed RCT should be conducted after developing a reliable study question by analyzing a sufficient number of observational studies. Overproduction of low-quality RCTs (65) has also been observed in the global evidence body of TCM (66).

### Evidence Gap and Implications for Future Studies

In summary, the overall evidence body was insufficient in the areas of TCM for post-stroke SBD and Chinese mind-body exercises for post-stroke insomnia. In addition, the current evidence is remarkably ambiguous to be translated into clinical practice, as few studies have focused on specific CHM interventions for post-stroke insomnia. Although the number of SRs on acupuncture for post-stroke insomnia was large, their quality was significantly low to reach a certain conclusion. In addition, we identified seven protocols of SRs and one protocol for RCT, most of which were assessing acupuncture therapies for post-stroke insomnia, and results of them were not published. Thus, future studies should be conducted in the following areas:

- Prioritizing high-quality clinical trials on specific CHM interventions and improving the quality of SRs on acupuncture for a better evidence translation to clinical practice of post-stroke insomnia
- Exploring the effect of TCM on SBDs after stroke and as the benefits of Chinese mind-body exercises for post-stroke insomnia for the generation of more clinical evidence of clinical significance and emergence.

## Limitations

This study has some limitations. We only electronically searched the English and Chinese literature databases, which may have omitted evidence from other sources.

## Data Availability Statement

The original contributions presented in the study are included in the article/Supplementary Material, further inquiries can be directed to the corresponding author/s.

## Author Contributions

QS conceived the study. QS, DZ, NG, and HL searched the literature and collected data. QS, DZ, and ZK analyzed the data. ZK and DZ contributed to the graphical presentation of the evidence map. XN designed the study, supervised the study implementation, and wrote the manuscript. All authors approved the final submission.

## Funding

This study was funded by the Scientific and Technological Research Project for Young Investigators from the Guangdong Provincial Hospital of Chinese Medicine, China (No. YN2019QL10, principal investigator: XN) and was additionally supported by the internal funding from Guangzhou University of Chinese Medicine (No. 2021xk26).

## Conflict of Interest

The authors declare that the research was conducted in the absence of any commercial or financial relationships that could be construed as a potential conflict of interest.

## Publisher's Note

All claims expressed in this article are solely those of the authors and do not necessarily represent those of their affiliated organizations, or those of the publisher, the editors and the reviewers. Any product that may be evaluated in this article, or claim that may be made by its manufacturer, is not guaranteed or endorsed by the publisher.

## Abbreviations

AMSTAR 2: *A MeaSurement Tool to Assess systematic Reviews-2*
CBT-i: cognitive-behavioral therapy for insomnia
CHM: Chinese herbal medicine
CPAP: continuous positive airway pressure
non-RCTs: non-randomized controlled clinical trials
PSI: post-stroke insomnia
PSSDs: post-stroke sleep disorders
RCTs: randomized controlled trials
RLS: restless leg syndrome
SBDs: sleep-related breathing disorders
SRs: systematic reviews
TCM: traditional Chinese medicine.
