# Supplementary material for: Traditional Chinese Medicine for Post-stroke Sleep Disorders: The Evidence Mapping of Clinical Studies
Source: Front Psychiatry. 2022 Jun 15;13:865630. doi: 10.3389/fpsyt.2022.865630 (PMC9240765; doi:10.3389/fpsyt.2022.865630)
Supplement: Supplementary file 1 [file Table_1.DOCX]

**Table S1. Search strategy**

| PubMed |
| --- |
| 1. “cerebrovascular disorders”[MeSH Terms] or “basal ganglia cerebrovascular disease”[MeSH Terms] or “carotid artery diseases”[MeSH Terms] or “cerebrovascular trauma”[MeSH Terms] or “intracranial arterial diseases”[MeSH Terms] or “stroke”[MeSH Terms] 2. “brain infarction”[MeSH Terms] or “brain ischemia”[MeSH Terms] or “intracranial embolism and thrombosis”[MeSH Terms] or “carotid artery thrombosis”[MeSH Terms] or “Ischemic Attack, Transient”[MeSH Terms] or “carotid artery, internal, dissection”[MeSH Terms] or “cerebral arterial diseases”[MeSH Terms] or “infarction, anterior cerebral artery”[MeSH Terms] or “infarction, middle cerebral artery”[MeSH Terms] or “infarction, posterior cerebral artery”[MeSH Terms] or "intracranial embolism and thrombosis”[MeSH Terms] or “vertebral artery dissection”[MeSH Terms] 3. “intracranial hemorrhages”[MeSH Terms] or “Cerebral Hemorrhage”[MeSH Terms] or “Intracranial Hemorrhage, Hypertensive”[MeSH Terms] or “Subarachnoid Hemorrhage”[MeSH Terms] 4. “Intracranial Aneurysm”[MeSH Terms] AND “Rupture, Spontaneous”[MeSH Terms] 5. (“aneurysm”[MeSH Terms] or “aneurysm, ruptured”[MeSH Terms] or “hematoma”[MeSH Terms]) and (“brain”[MeSH Terms] or “meninges”[MeSH Terms]) 6. “Vasospasm, Intracranial”[MeSH Terms] 7. #1 OR #2 OR #3 OR #5 OR #5 OR #6 8. ((ischaemi*[Title/Abstract] OR ischemi*[Title/Abstract]) AND (stroke*[Title/Abstract] or apoplexy*[Title/Abstract] or (cerebral vasc*[Title/Abstract]) or cerebrovasc*[Title/Abstract] or cva[Title/Abstract] or attack*[Title/Abstract])) 9. ((brain[Title/Abstract] or cerebr*[Title/Abstract] or cerebell*[Title/Abstract] or vertebrobasil*[Title/Abstract] or hemispher*[Title/Abstract] or intracran*[Title/Abstract] or intracerebral[Title/Abstract] or infratentorial[Title/Abstract] or supratentorial[Title/Abstract] or "middle cerebr*"[Title/Abstract] or mca*[Title/Abstract] or "anterior circulation"[Title/Abstract]) AND (ischaemi*[Title/Abstract] OR ischemi*[Title/Abstract] or infarct*[Title/Abstract] or thrombo*[Title/Abstract] or emboli*[Title/Abstract] or occlus*[Title/Abstract] or hypoxi*[Title/Abstract])) 10. ((brain*[Title/Abstract] or cerebr*[Title/Abstract] or cerebell*[Title/Abstract] or intracerebral[Title/Abstract] or "basal ganglia"[Title/Abstract] or intracranial[Title/Abstract] or subarachnoid[Title/Abstract] or arachnoid[Title/Abstract] or putaminal[Title/Abstract]) AND (haemorrhage*[Title/Abstract] or hemorrhage*[Title/Abstract] or haematoma*[Title/Abstract] or hematoma*[Title/Abstract] or bleed*[Title/Abstract] or blood*[Title/Abstract])) 11. (cerebral[Title/Abstract] or intracranial[Title/Abstract] or cerebrovascular[Title/Abstract]) AND (vasospasm[Title/Abstract] or spasm[Title/Abstract]) 12. sah[Title/Abstract] OR ich[Title/Abstract] OR sich[Title/Abstract] 13. ((transi*[Title/Abstract] and (ischaem*[Title/Abstract] or ischem*[Title/Abstract]) and attack*[Title/Abstract]) or TIA*[Title/Abstract]) 14. #7 or #8 or #9 or #10 or #11 or #12 or #13 15. "sleep"[MeSH Terms] OR "Sleep Bruxism"[MeSH Terms] OR "kleine-levin syndrome"[MeSH Terms] OR "Restless Legs Syndrome"[MeSH Terms] OR "Narcolepsy"[MeSH Terms] OR "cataplexy "[MeSH Terms] OR "sleep apnea syndromes"[MeSH Terms] OR "disorders of excessive somnolence "[MeSH Terms] OR "sleep Initiation and Maintenance Disorders"[MeSH Terms] OR "sleep arousal disorders"[MeSH Terms] OR "Sleep Paralysis"[MeSH Terms] OR "sleep-wake transition disorders"[MeSH Terms] OR "Night Terrors"[MeSH Terms] OR "sleep Wake Disorders"[MeSH Terms] 16. ((circadian[Title/Abstract] OR insomnia*[Title/Abstract] OR hypersomnia[Title/Abstract] OR parasomnia*[Title/Abstract] OR wakefulness[Title/Abstract] OR hypersomn*[Title/Abstract] OR somnolence*[Title/Abstract] OR somnambul*[Title/Abstract] OR "Chronobiology Disorders"[Title/Abstract]) OR "rest‐activity"[Title/Abstract] OR sundowning[Title/Abstract] OR insomnolence[Title/Abstract] OR ((sleep[Title/Abstract]) AND (disorder*[Title/Abstract] OR problem*[Title/Abstract] OR pattern*[Title/Abstract] OR onset[Title/Abstract] OR TIM*[Title/Abstract])) OR ("Jet-Lag Syndrome"[Title/Abstract])) 17. (insomn*[Title/Abstract] OR dyssomni*[Title/Abstract] OR ("sleep impact scale"[Title/Abstract] OR "sleep questionnaire"[Title/Abstract] OR "sleep scale"[Title/Abstract] OR "sleep evaluation questionnaire"[Title/Abstract] OR "sleep quality index"[Title/Abstract] OR PSQI[Title/Abstract] OR "sleep impairment index"[Title/Abstract] OR "sleepiness scale"[Title/Abstract] OR "sleep log"[Title/Abstract] OR "sleep diar*"[Title/Abstract] OR "sleep Deprivation"[Title/Abstract] OR "sleep Stage*"[Title/Abstract])) 18. (((sleep[Title/Abstract]) AND (initiation[Title/Abstract] OR maintenance[Title/Abstract])) OR ((nocturnal[Title/Abstract]) AND (wake*[Title/Abstract] OR awake*[Title/Abstract]))) 19. (((sleep[Title/Abstract]) AND (apnea[Title/Abstract] OR apnoea[Title/Abstract] OR hypopnea[Title/Abstract] OR hypopnoea[Title/Abstract])) OR OSA[Title/Abstract] OR OSAHS[Title/Abstract] OR "sleep disordered breathing"[Title/Abstract] OR snor*[Title/Abstract] OR narcole*[Title/Abstract] OR cataple*[Title/Abstract] OR gelineau*[Title/Abstract] OR anti?cataple*[Title/Abstract] OR anticataple*[Title/Abstract]) 20. (((restless[Title/Abstract]) AND (leg*[Title/Abstract])) OR ((ekbom*[Title/Abstract]) AND (syndrome[Title/Abstract])) OR "willis-ekbom"[Title/Abstract] OR ((periodic[Title/Abstract] OR nocturnal[Title/Abstract]) AND (leg[Title/Abstract] OR limb[Title/Abstract]) AND (movements*[Title/Abstract])) OR (PLM[Title/Abstract] OR PLMS[Title/Abstract] OR PLMD[Title/Abstract] OR RLS[Title/Abstract]) OR "psychomotor agitation"[Title/Abstract] OR ((peripheral[Title/Abstract]) AND (polyneuropath*[Title/Abstract])) OR ((nocturnal[Title/Abstract]) AND (movement*[Title/Abstract] OR myoclonus[Title/Abstract]))) 21. (((periodic[Title/Abstract]) AND (hypersomnia[Title/Abstract]) AND (sleep*[Title/Abstract])) OR KLS[Title/Abstract] OR bruxis*[Title/Abstract] OR bruxe*[Title/Abstract] OR bruxist*[Title/Abstract] OR "insufficient sleep"[Title/Abstract] OR "poor sleep quality"[Title/Abstract]) 22. #15 or #16 or #17 or #18 or #19 or #20 or #21 23. #14 and #22 |
| EMBASE |
| 1. cerebrovascular disease'/exp OR 'basal ganglia cerebrovascular disease'/exp OR 'carotid artery diseases'/exp OR 'cerebrovascular trauma'/exp OR 'intracranial arterial diseases'/exp OR 'stroke'/exp 2. brain infarction'/exp OR 'brain ischemia'/exp OR 'intracranial embolism and thrombosis'/exp OR 'carotid artery thrombosis'/exp OR 'Ischemic Attack, Transient'/exp OR 'carotid artery, internal, dissection'/exp OR 'cerebral arterial diseases'/exp OR 'infarction, anterior cerebral artery'/exp OR 'infarction, middle cerebral artery'/exp OR 'infarction, posterior cerebral artery'/exp OR 'intracranial embolism and thrombosis'/exp OR 'vertebral artery dissection'/exp 3. intracranial hemorrhages'/exp OR 'Cerebral Hemorrhage'/exp OR 'Intracranial Hemorrhage, Hypertensive'/exp OR 'Subarachnoid Hemorrhage'/exp 4. Intracranial Aneurysm'/exp AND 'Rupture, Spontaneous'/exp 5. aneurysm'/exp OR 'aneurysm, ruptured'/exp OR 'hematoma'/exp AND 'brain'/exp OR 'meninges'/exp 6. Vasospasm, Intracranial '/exp 7. #1 OR #2 OR #3 OR #4 OR #5 OR #6 8. ((ischaemi*:ti,ab,kw OR ischemi*:ti,ab,kw) AND (stroke*:ti,ab,kw or apoplexy*:ti,ab,kw or (cerebral vasc*:ti,ab,kw) or cerebrovasc*:ti,ab,kw or cva:ti,ab,kw or attack*:ti,ab,kw)) 9. ((brain:ti,ab,kw or cerebr*:ti,ab,kw or cerebell*:ti,ab,kw or vertebrobasil*:ti,ab,kw or hemispher*:ti,ab,kw or intracran*:ti,ab,kw or intracerebral:ti,ab,kw or infratentorial:ti,ab,kw or supratentorial:ti,ab,kw or 'middle cerebr*':ti,ab,kw or mca*:ti,ab,kw or 'anterior circulation':ti,ab,kw) AND (isch?emi*:ti,ab,kw or infarct*:ti,ab,kw or thrombo*:ti,ab,kw or emboli*:ti,ab,kw or occlus*:ti,ab,kw or hypoxi*:ti,ab,kw)) 10. (brain*:ti,ab,kw or cerebr*:ti,ab,kw or cerebell*:ti,ab,kw or intracerebral:ti,ab,kw or 'basal ganglia':ti,ab,kw or intracranial:ti,ab,kw or subarachnoid:ti,ab,kw or arachnoid:ti,ab,kw or putaminal:ti,ab,kw) AND (haemorrhage*:ti,ab,kw or hemorrhage*:ti,ab,kw or haematoma*:ti,ab,kw or hematoma*:ti,ab,kw or bleed*:ti,ab,kw or blood*:ti,ab,kw) 11. ((cerebral:ti,ab,kw or intracranial:ti,ab,kw or cerebrovascular:ti,ab,kw) AND (vasospasm:ti,ab,kw or spasm:ti,ab,kw)) 12. sah:ti,ab,kw OR ich:ti,ab,kw OR sich:ti,ab,kw 13. ((transi*:ti,ab,kw and (ischaem*:ti,ab,kw or ischem*:ti,ab,kw) and attack*:ti,ab,kw) or TIA$:ti,ab,kw) 14. #7 or #8 or #9 or #10 or #11 or #12 or #13 15. sleep'/exp OR 'sleep bruxism'/exp OR 'kleine‐levin syndrome' OR 'restless legs syndrome'/exp OR 'narcolepsy'/exp OR 'cataplexy'/exp OR 'sleep apnea'/exp OR 'disorders of excessive somnolence'/exp OR 'sleep disorders'/exp OR 'sleep initiation and maintenance disorders'/exp OR 'sleep arousal disorders'/exp OR 'sleep paralysis'/exp OR 'sleep-wake transition disorders'/exp OR 'night terrors'/exp 16. ((circadian OR insomnia* OR hypersomnia OR parasomnia* OR wakefulness OR hypersomn* OR somnolence* OR somnambul* OR "Chronobiology Disorders") OR "rest‐activity" OR sundowning OR insomnolence OR "sleep disorder*" OR "sleep problem*" OR (sleep TIM*) OR (Jet-Lag Syndrome)):ti,ab,kw 17. (insomn* OR dyssomni* OR ("sleep impact scale" OR "sleep questionnaire" OR "sleep scale" OR "sleep evaluation questionnaire" OR "sleep quality index" OR PSQI OR "sleep impairment index" OR "sleepiness scale" OR "sleep log" OR "sleep diar*" OR "sleep deprivation" OR "sleep Stage*")):ti,ab,kw 18. ((sleep and (initiation OR maintenance)) OR (nocturnal (wake* OR awake*))):ti,ab,kw 19. ((sleep AND (apnea OR apnoea OR hypopnea OR hypopnoea)) OR OSA OR OSAHS OR "sleep disordered breathing" OR snor$ OR narcole* OR cataple* OR gelineau* OR anti?cataple*):ti,ab,kw 20. ("restless leg$" OR (ekbom* AND syndrome) OR "willis-ekbom" OR ("periodic leg movement$" OR "periodic limb movement$" OR "nocturnal leg movement$" OR "nocturnal limb movement$") OR "ur?emic polyneuropath*" OR "ur?emic neuropath*" OR (PLM OR PLMS OR PLMD OR RLS) OR ((peripheral) AND (polyneuropath*)) OR "nocturnal movement$" OR "nocturnal myoclonus"):ti,ab,kw 21. ((periodic AND hypersomnia AND sleep*) OR KLS OR bruxis* OR bruxe* OR bruxist* OR "insufficient sleep" OR "poor sleep quality"):ti,ab,kw 22. #15 or #16 or #17 or #18 or #19 or #20 or #21 23. #14 and #22 |
| Cochrane Library |
| 1. [mh “cerebrovascular disorders”] or [mh “basal ganglia cerebrovascular disease”] or [mh “carotid artery diseases”] or [mh “cerebrovascular trauma”] or [mh “intracranial arterial diseases”] or [mh “stroke”] 2. [mh “brain infarction”] or [mh “brain ischemia”] or [mh “intracranial embolism and thrombosis”] or [mh “carotid artery thrombosis”] or [mh “Ischemic Attack, Transient”] or [mh “carotid artery, internal, dissection”] or [mh “cerebral arterial diseases”] or [mh “infarction, anterior cerebral artery”] or [mh “infarction, middle cerebral artery”] or [mh “infarction, posterior cerebral artery”] or [mh “intracranial embolism and thrombosis”] or [mh “vertebral artery dissection”] 3. [mh “intracranial hemorrhages”] or [mh “Cerebral Hemorrhage”] or [mh “Intracranial Hemorrhage, Hypertensive”] or [mh “Subarachnoid Hemorrhage”] 4. [mh “Intracranial Aneurysm”] AND [mh “Rupture, Spontaneous”] 5. ([mh “aneurysm”] or [mh “aneurysm, ruptured”] or [mh “hematoma”]) AND ([mh “brain”] or [mh “meninges”]) 6. [mh “Vasospasm, Intracranial”] 7. #1 OR #2 OR #3 OR #5 OR #5 OR #6 8. (isch?emi* AND (stroke* or apoplexy* or cerebral vasc* or cerebrovasc* or cva or attack*)).tw. 9. ((brain or cerebr* or cerebell* or vertebrobasil* or hemispher* or intracran* or intracerebral or infratentorial or supratentorial or middle cerebr* or mca* or anterior circulation) AND (isch?emi* or infarct* or thrombo* or emboli* or occlus* or hypoxi*)).tw. 10. ((brain* or cerebr* or cerebell* or intracerebral or “basal ganglia” or intracranial or subarachnoid or arachnoid or putaminal) AND (haemorrhage* or hemorrhage* or haematoma* or hematoma* or bleed* or blood*)).tw. 11. ((cerebral or intracranial or cerebrovascular) AND (vasospasm or spasm)).tw. 12. (sah OR ich OR sich).tw. 13. ((transi* and isch?em* and attack*) or TIA*).tw. 14. #7 or #8 or #9 or #10 or #11 or #12 or #13 15. [mh "sleep Disorders"] OR [mh "sleep"] OR [mh "Sleep Bruxism"] OR [mh "Kleine‐Levin Syndrome"] OR [mh "Restless Legs Syndrome"] OR [mh "Narcolepsy"] OR [mh "Cataplexy"] OR [mh "sleep Apnea"] OR [mh "Disorders of Excessive Somnolence"] OR [mh "sleep Initiation and Maintenance Disorders"] OR [mh "sleep arousal disorders"] OR [mh "Sleep Paralysis"] OR [mh "Sleep-Wake Transition Disorders"] OR [mh "Night Terrors"] 16. ((circadian OR insomnia* OR hypersomnia OR parasomnia* OR wakefulness OR hypersomn* OR somnolence* OR somnambul* OR Chronobiology Disorders) OR (“rest‐activity” OR insomnolence OR sundowning) OR (sleep near disorder*) OR (sleep near problem*) OR (sleep near pattern*) OR (sleep near onset) OR (sleep near TIM*) OR (Jet-Lag Syndrome)):ti,ab,kw 17. (insomn* OR dyssomni* OR ("sleep impact scale" OR "sleep questionnaire" OR "sleep scale" OR "sleep evaluation questionnaire" OR "sleep quality index" OR PSQI OR "sleep impairment index" OR "sleepiness scale" OR "sleep log" OR "sleep diar*" OR "sleep Deprivation" OR "sleep Stage*")):ti,ab,kw 18. ((sleep NEAR (initiation OR maintenance)) OR (nocturnal NEXT (wake* OR awake*))):ti,ab,kw 19. ((sleep and (apnea OR apnoea OR hypopnea OR hypopnoea)) OR (OSA OR SHS OR OSAHS) OR ("upper airway resistance" OR "sleep disordered breathing" OR snor*) OR (narcole* OR cataple*OR gelineau* OR anti‐cataple* OR anticataple*)):ti,ab,kw 20. ((restless next leg*) OR (ekbom* next syndrome) OR (willis-ekbom) OR ((periodic OR nocturnal) next (leg OR limb) next movements*) OR (PLM OR PLMS OR PLMD OR RLS) OR "psychomotor agitation" OR (ur*emic next (polyneuropath* OR neuropath*)) OR (peripheral next polyneuropath*) OR (nocturnal next (movement* OR myoclonus))):ti,ab,kw 21. ((periodic next hypersomnia next sleep*) OR (compulsive next eating*) OR (hyperphagia OR megaphagia OR polyphagia* OR hypersexuality OR KLS) OR (bruxis* OR bruxe*) OR (teeth adj3 grind*) OR (tooth adj3 grind*) OR (teeth adj3 clench*) OR (tooth adj3 clench*)):ti,ab,kw 22. sleep:ti 23. #15 or #16 or #17 or #18 or #19 or #20 or #21 or #22 24. #14 and #23 |
| CBM (Chinese database) |
| 1. (((("脑血管障碍"[不加权:扩展]) OR "颈动脉疾病"[不加权:扩展]) OR "颅内栓塞和血栓形成"[不加权:扩展]) OR "脑缺血"[不加权:扩展]) OR "脑缺血发作, 短暂性"[不加权:扩展] 2. (((("脑梗死"[不加权:扩展]) OR "脑血管基底神经节疾病"[不加权:扩展]) OR "脑血管基底神经节出血"[不加权:扩展]) OR "壳核出血"[不加权:扩展]) OR "脑血管损伤"[不加权:扩展] 3. ((((( "椎动脉破裂"[不加权:扩展]) OR "颅内出血"[不加权:扩展]) OR "脑出血"[不加权:扩展]) OR "蛛网膜下腔出血"[不加权:扩展]) OR "颅内出血, 高血压性"[不加权:扩展]) OR "颅内动脉疾病"[不加权:扩展] 4. ((((("颅内动脉瘤"[不加权:扩展]) OR "卒中"[不加权:扩展]) OR"血管痉挛, 颅内"[不加权:扩展])OR "中风"[不加权:扩展]) OR "中风先兆症"[不加权:扩展]) OR "中风后遗症"[不加权:扩展] 5. #1 or #2 or #3 or #4 6. ((((((((("颅内栓塞"[摘要:智能]) OR "颅内血栓形成"[摘要:智能]) OR "脑血栓形成"[摘要:智能]) OR "脑栓塞"[摘要:智能]) OR "脑缺血"[摘要:智能]) OR "短暂性脑缺血发作"[摘要:智能]) OR "脑梗死"[摘要:智能]) OR “脑梗塞”[摘要:智能]) OR "基底节梗死"[摘要:智能]) OR "基底节出血"[摘要:智能] 7. (((("高血压性脑出血"[摘要:智能]) OR "原发性脑出血"[摘要:智能]) OR "自发性脑出血"[摘要:智能]) OR "蛛网膜下腔出血"[摘要:智能]) OR "高血压性颅内出血"[摘要:智能] 8. (((((("颅内动脉瘤破裂"[摘要:智能]) OR "卒中"[摘要:智能]) OR "颅内血管痉挛"[摘要:智能]) OR “急性脑血管病”[摘要:智能])OR "中风"[摘要:智能]) OR "中风先兆症"[摘要:智能]) OR "中风后遗症"[摘要:智能] 9. ((((("壳核出血"[摘要:智能]) OR "脑血管损伤"[摘要:智能]) OR "椎动脉夹层"[摘要:智能]) OR "颈动脉夹层"[摘要:智能]) OR "颅内出血"[摘要:智能]) OR "脑出血"[摘要:智能] 10. #5 or #6 or #7 or #8 or #9 11. ((((((((((((((("睡眠觉醒障碍"[不加权:扩展]) OR "深眠状态"[不加权:扩展]) OR "不宁腿综合征"[不加权:扩展]) OR "夜间性突发性张力障碍"[不加权:扩展]) OR "夜磨牙症"[不加权:扩展]) OR "睡眠唤醒障碍"[不加权:扩展]) OR "梦行症"[不加权:扩展]) OR "夜惊"[不加权:扩展]) OR "夜间性肌阵挛综合征"[不加权:扩展]) OR "睡眠-觉醒过渡障碍"[不加权:扩展]) OR "REM深眠状态"[不加权:扩展]) OR "REM睡眠行为障碍"[不加权:扩展]) OR "睡眠麻痹"[不加权:扩展]) OR "睡眠剥夺"[不加权:扩展]) OR "睡眠障碍, 昼夜节律性"[不加权:扩展]) OR "飞行时差反应综合征"[不加权:扩展] 12. (((((((((((("睡眠障碍, 内源性"[不加权:扩展]) OR "过度嗜睡性障碍"[不加权:扩展]) OR "发作性睡病"[不加权:扩展]) OR "猝倒症"[不加权:扩展]) OR "克莱恩-莱文综合征"[不加权:扩展]) OR "嗜睡症, 特发性"[不加权:扩展]) OR "入睡和睡眠障碍"[不加权:扩展]) OR "失眠症, 致死性家族性"[不加权:扩展]) OR "不宁腿综合征"[不加权:扩展]) OR "睡眠呼吸暂停综合征"[不加权:扩展]) OR "睡眠呼吸暂停, 阻塞性"[不加权:扩展]) OR "肥胖低通气综合征"[不加权:扩展]) OR "睡眠呼吸暂停, 中枢性"[不加权:扩展] 13. ((((("睡眠障碍"[不加权:扩展]) OR "梦行症"[不加权:扩展]) OR "梦交"[不加权:扩展]) OR "睡眠过度"[不加权:扩展]) OR "失眠症"[不加权:扩展]) OR "多梦"[不加权:扩展] 14. ((((((((("睡眠倒错"[摘要:智能]) OR "梦呓症"[摘要:智能]) OR "梦游症"[摘要:智能]) OR "夜游"[摘要:智能]) OR "梦魇"[摘要:智能]) OR "入睡困难"[摘要:智能]) OR "夜惊"[摘要:智能]) OR "梦游"[摘要:智能]) OR "梦魇"[摘要:智能]) OR "夜啼"[摘要:智能] 15. "异态睡眠"[摘要:智能] OR "不寐"[摘要:智能] OR "睡眠增多"[摘要:智能] OR "睡行症"[摘要:智能] OR "发作性睡病"[摘要:智能] OR "昼夜节律失调"[摘要:智能] OR "睡眠减少"[摘要:智能] OR "睡眠过多"[摘要:智能] OR "梦行症"[摘要:智能] OR "觉醒异常"[摘要:智能] 16. #11 or #12 or #13 or #14 or #15 17. #10 and #16 |
| CNKI (Chinese database) |
| SU=('脑血管障碍'+'颅内栓塞'+'卒中'+'脑出血'+'颅内出血'+'壳核出血'+'蛛网膜下腔出血'+'脑缺血'+'颅内血栓形成'+'脑血栓形成'+'脑实质出血'+'短暂性脑缺血发作'+'壳核出血'+'蛛网膜下腔出血'+'中风'+'中风后遗症'+'脑栓塞'+'基底节梗死'+'基底节出血'+'脑血管损伤'+'椎动脉夹层'+'颈动脉夹层'+'颅内动脉瘤'+'颅内血管痉挛'+'急性脑血管病'+'中风先兆症'+'颈动脉疾病'+'脑血管基底神经节疾病'+'椎动脉破裂'+'颅内动脉疾病')  SU=('睡眠觉醒障碍'+'不宁腿综合征'+'夜间性突发性张力障碍'+'夜间性肌阵挛综合征'+'夜磨牙'+'睡眠唤醒障碍'+'睡眠障碍, 昼夜节律性'+'飞行时差反应综合征'+'梦行'+'夜惊'+'REM深眠状态'+'REM睡眠行为障碍'+'睡眠异常'+'睡眠剥夺'+'嗜睡'+'入睡障碍'+'睡眠呼吸暂停'+'猝倒症'+'发作性睡病'+'莱恩-莱文综合征'+'失眠'+'肥胖低通气综合征'+'睡眠-觉醒过渡障碍'+'睡眠倒错'+'梦游'+'梦呓'+'觉醒异常'+'夜游'+'梦魇'+'入睡'+'夜惊'+'夜啼'+'睡眠过多'+'睡眠减少'+'不寐'+'睡眠增多'+''+'异态睡眠'+'睡行'+'昼夜节律失调'+'发作性睡病'+'梦交'+'睡眠过度'+'多梦') |
| Wanfang (Chinese database) |
| (摘要: 脑血管障碍+颅内栓塞+卒中+脑出血+颅内出血+壳核出血+蛛网膜下腔出血+脑缺血+脑梗死+颅内血栓形成+脑血栓形成+脑实质出血+短暂性脑缺血发作+壳核出血+中风+脑栓塞+基底节梗死+基底节出血+脑血管损伤+颅内动脉瘤破裂+颅内血管痉挛+急性脑血管病)*(摘要: 睡眠觉醒障碍+不宁腿综合征+夜间性突发性张力障碍+夜间性肌阵挛综合征+夜磨牙+睡眠唤醒障碍+睡眠障碍, 昼夜节律性+飞行时差反应综合征+梦行+夜惊+REM深眠状态+REM睡眠行为障碍+睡眠异常+睡眠剥夺+嗜睡+入睡障碍+睡眠呼吸暂停+猝倒症+发作性睡病+莱恩-莱文综合征+失眠+肥胖低通气综合征+睡眠-觉醒过渡障碍+睡眠倒错+梦游+梦呓+觉醒异常+夜游+梦魇+入睡+夜惊+夜啼+睡眠过多+睡眠减少+不寐+睡眠增多+异态睡眠+睡行+昼夜节律失调+发作性睡病)  (摘要: "脑血管障碍"+"颅内栓塞"+"卒中"+"脑出血"+"颅内出血"+"壳核出血"+"蛛网膜下腔出血"+"脑缺血"+"脑梗死"+"颅内血栓形成"+"脑血栓形成"+"脑实质出血"+"短暂性脑缺血发作"+"壳核出血"+"中风"+"脑栓塞"+"基底节梗死"+"基底节出血"+"脑血管损伤"+"颅内动脉瘤破裂"+"颅内血管痉挛"+"急性脑血管病")*(摘要: "睡眠觉醒障碍"+"不宁腿综合征"+"夜间性突发性张力障碍"+"夜间性肌阵挛综合征"+"夜磨牙"+"睡眠唤醒障碍"+"睡眠障碍,昼夜节律性"+"飞行时差反应综合征"+"梦行"+"夜惊"+"REM深眠状态"+"REM睡眠行为障碍"+"睡眠异常"+"睡眠剥夺"+"嗜睡"+"入睡障碍"+"睡眠呼吸暂停"+"猝倒症"+"发作性睡病"+"莱恩-莱文综合征"+"失眠"+"肥胖低通气综合征"+"睡眠-觉醒过渡障碍"+"睡眠倒错"+"梦游"+"梦呓"+"觉醒异常"+"夜游"+"梦魇"+"入睡"+"夜惊"+"夜啼"+"睡眠过多"+"睡眠减少"+"不寐"+"睡眠增多"+"异态睡眠"+"睡行"+"昼夜节律失调"+"发作性睡病") |
