# Supplementary material for: Traditional Chinese Medicine for Post-stroke Sleep Disorders: The Evidence Mapping of Clinical Studies
Source: Front Psychiatry. 2022 Jun 15;13:865630. doi: 10.3389/fpsyt.2022.865630 (PMC9240765; doi:10.3389/fpsyt.2022.865630)
Supplement: Supplementary file 2 [file Table_2.DOCX]

**Table S2. The list of included studies**

| 1. 符世纯.大定风珠治疗中风后遗不寐36例[J].中医函授通讯,1993(05):43.DOI:10.13193/j.archtcm.1993.05.45.fushch.039. 2. 朱沁,宋德勇.水针治疗中风后失眠67例临床观察[J].针灸临床杂志,1996(Z1):76. 3. 蔡朗.调肝安神针刺法治疗中风不寐45例[J].中国针灸,1996(11):25. 4. 王玉龙.头面部穴位按摩治疗中风病人的睡眠障碍[J].现代康复,1998(02):104-105. 5. 袁怀同.升降阴阳法治疗中风病睡眠异常[J].中医杂志,1998(10):595.DOI:10.13288/j.11-2166/r.1998.10.014. 6. 阮经文,郑沛仪.针灸疗法对合并睡眠障碍的早期脑卒中患者运动功能的影响[J].中国康复理论与实践,2000(02):26-29. 7. 郭二霞.镇肝熄风汤加减治疗中风后睡眠颠倒36例[J].安徽中医临床杂志,2002(06):456-457.DOI:10.16448/j.cjtcm.2002.06.018. 8. 郭福新,李如奎,许红. 落花生枝叶制剂对缺血性中风后失眠烦躁不安疗效观察[C]//第四次全国中西医结合神经系统疾病学术研讨会论文集.[出版者不详],2002:100. 9. 邱锡采.冠心苏合胶囊治疗脑梗塞后嗜睡21例[J].浙江中医学院学报,2002(06):41.DOI:10.16466/j.issn1005-5509.2002.06.028. 10. 郭毅坚,李俐.穴位注射治疗中风后遗症患者失眠30例[J].福建中医药,2003(06):42-43. 11. 李秀霞,赵小丽,宋王浩.松龄血脉康胶囊治疗心血管疾病时对失眠症状的改善[J].华西药学杂志,2003(06):473-474.DOI:10.13375/j.cnki.wcjps.2003.06.035. 12. 王寅,赵志付,伍昱,赵阳,李以松,张维,赵宏,杨涛,刘元石,彭唯娜,张翠彦,刘晓清,刘军.针刺治疗脑卒中后抑郁失眠临床疗效评价[J].中国针灸,2004(09):13-16. 13. 江浩.推拿手法配合中药治疗老年脑卒中后失眠89例疗效观察[J].云南中医中药杂志,2004(04):26-27.DOI:10.16254/j.cnki.53-1120/r.2004.04.030. 14. 冯蓓蕾,王翘楚,李正元.落花安神合剂治疗脑卒中后失眠症的临床观察[J].江苏中医药,2004(07):21-22. 15. 王春霞,王小亮.黄连温胆汤加减治疗脑卒中急性期睡眠障碍32例[J].现代中西医结合杂志,2004(19):2569. 16. 齐宇,何春慧.耳针治疗中风后失眠36例疗效观察[J].中国冶金工业医学杂志,2004(03):74. 17. 刘翠玲,张颖.黄芪注射液治疗心脑血管病时失眠症状改善的临床观察[J].中国民间疗法,2005(12):36-37.DOI:10.19621/j.cnki.11-3555/r.2005.12.041. 18. 王杰超,宋霄,杨爽,孙文亮,吕晓红.黄芪注射液治疗心脑血管病合并失眠症的作用机理[J].中国全科医学,2005(02):146-147. 19. 赖真,蔡智刚,耿小茵,王耀邦.刺五加对椎-基底动脉供血不足患者失眠症状疗效观察[J].辽宁中医杂志,2005(12):1231-1232.DOI:10.13192/j.ljtcm.2005.12.10.laizh.005. 20. 邓国鹏,吴远华.自拟通脑清心汤治疗脑卒中后抑郁失眠28例[J].贵阳中医学院学报,2006(01):26-27.DOI:10.16588/j.cnki.issn1002-1108.2006.01.016. 21. 吴济建,俞亮,金淑玲.中风后不寐的中西医治疗[J].浙江中西医结合杂志,2006(08):520. 22. 刘健红,黄坚红,陈秀慧.针刺治疗中风后失眠32例疗效观察[J].国际医药卫生导报,2006(16):107-108. 23. 戴晓矞,李妍,宋秋珍,韩宝杰.温针髀关治疗中风后不宁腿综合征疗效观察[J].上海针灸杂志,2006(01):23-24.DOI:10.13460/j.issn.1005-0957.2006.01.011. 24. 罗试计.通窍活血汤治疗脑梗死后失眠23例[J].广西中医药,2006(05):35. 25. 高震,胡志诚.活血养血安神汤治疗脑梗死致倒错性嗜眠45例[J].陕西中医,2006(02):171-172. 26. 冯冬泽.电针、紫外钱光量子透氧并西药内服治疗脑卒中后抑郁失眠症52例[J].国医论坛,2006(02):36-37. 27. 龚道恺,李云文. 百乐眠胶囊治疗卒中后失眠的临床观察[C]. 2006. 28. 戴晓矞,李妍,宋秋珍,韩宝杰,肖元春.Clinical Observation of Warm Acupuncture at Biguan(ST 31)in Treating Post-apoplectic Restless Legs Syndrome[J].Journal of Acupuncture and Tuina Science,2006,4(03):174-175. 29. 包红伟,郭燕,孟永生,李彦琴.滋补肝肾、活血安神法治疗卒中后睡眠障碍58例临床观察[J].北京中医,2007(05):296-297. 30. 任海英,尹毓梅. 重用主药酸枣仁汤对脑血栓患者失眠的临床疗效观察[J]. 中国当代医学,2007(10). 31. 周生花.中西医结合治疗脑卒中合并睡眠呼吸暂停综合征临床观察[J].中国误诊学杂志,2007(21):4995-4996. 32. 李泰标. 针刺治疗脑卒中后失眠疗效观察[J]. 中国康复理论与实践,2007(7). DOI:10.3969/j.issn.1006-9771.2007.07.026. 33. 刘红霞,殷春萍.阴阳分时法治疗脑卒中后睡眠觉醒节律紊乱40例疗效观察[J].新中医,2007(10):31-32.DOI:10.13457/j.cnki.jncm.2007.10.014. 34. 卢琰琰. 益肾调督养心针法治疗脑卒中后失眠症的临床研究[D]. 2007. 35. 郭湘芳,粟俊,吕晶.通塞消栓汤治疗脑梗死兼阻塞性睡眠呼吸暂停低通气综合征30例[J].中国中医药信息杂志,2007(06):13-14. 36. 陈威,万于军,姚灿坤,王鑫.芪棱汤对脑梗死后失眠及神经功能康复的影响[J].中国中医急症,2007(06):643-644. 37. 刘占涛,曹东升,赵建利.加用增液除烦汤治疗脑卒中后日间过度倦睡症临床观察[J].广西中医药,2007(04):20-21. 38. 王蕾,陈琼,范超平.参松养心胶囊治疗脑卒中后失眠症的疗效观察[J].中国实用神经疾病杂志,2007(05):108. 39. 杨梅. 安神定志中药干预治疗脑卒中恢复期睡眠障碍39例[J]. 健康大视野,2013(10). 40. 刘宏雅,薛辉.天智颗粒治疗脑卒中后失眠症的疗效观察[J].中国实用神经疾病杂志,2008(10):117-118. 41. 韩玉林. 脑卒中患者失眠的中西医结合治疗[J]. 吉林医学,2008(23). DOI:10.3969/j.issn.1004-0412.2008.23.073. 42. 高震,曹利民,叶维霞.豁痰解毒安神汤治疗脑卒中后睡眠障碍38例观察[J].实用中医药杂志,2008(03):142. 43. 梁保民.耳穴压丸治疗脑梗死恢复期睡眠障碍47例[J].河南中医,2008(11):77-78.DOI:10.16367/j.issn.1003-5028.2008.11.034. 44. 马涛.电针治疗中风后不宁腿综合征19例[J].江苏中医药,2008(01):62. 45. 郑修广,彭念寅.刺五加注射液治疗心脑血管病伴失眠患者疗效观察[J].临床心身疾病杂志,2008(02):163-164. 46. 易世宏,陈宝田.安眠方对脑卒中恢复期失眠和神经功能康复的影响[J].中国康复医学杂志,2008(05):408-409. 47. 邓小兰,赵红.针刺治疗脑卒中后日间过度倦睡症临床观察[J].湖北中医杂志,2009,31(11):65-66. 48. 曹铁民.针刺治疗脑卒中伴睡眠倒错的临床观察[J].辽宁中医药大学学报,2009,11(05):147-148.DOI:10.13194/j.jlunivtcm.2009.05.149.caotm.029. 49. 艾诗奇,吴波.针刺太冲治疗脑卒中后丑时失眠烦躁[J].中国针灸,2009,29(07):546. 50. 高宇飞,万裕萍,徐骁.针刺十三鬼穴治疗中风后失眠的临床观察[J].湖北中医杂志,2009,31(05):24-25. 51. 王欣.益心舒胶囊治疗脑卒中后失眠症的疗效观察[J].中华中医药学刊,2009,27(06):1343-1344.DOI:10.13193/j.archtcm.2009.06.224.wangx.038. 52. 张俊杰. 穴位埋线治疗脑卒中恢复期患者失眠临床疗效观察[D].广州中医药大学,2009. 53. 米建平,余焯燊,赵晓红.醒脑调脏法治疗中风后失眠40例疗效观察[J].辽宁中医杂志,2009,36(10):1723-1724.DOI:10.13192/j.ljtcm.2009.10.95.mijp.027. 54. 刘红霞,吴中秋,殷春萍.脑卒中后睡眠觉醒障碍的阴阳分时治疗分析[J].河北中医,2009,31(03):385-386. 55. 杨姗杉,夏圣梅,刘江华.黄芪酸枣仁为主治疗脑卒中后睡眠障碍86例临床观察[J].中医杂志,2009,50(S1):140-141. 56. 刘耀东,王敬华,孙丽萍,段海平,姬广伟.归脾汤加减治疗中风后失眠[J].中国民间疗法,2009,17(04):34.DOI:10.19621/j.cnki.11-3555/r.2009.04.035. 57. 刘芳.大定风珠加味治疗中风后失眠30例[J].新中医,2009,41(12):70-71. 58. 杨孝东,齐国豪,林斌.百乐眠胶囊治疗脑卒中后失眠38例疗效观察[J].临床医药实践,2009,18(26):2047-2048. 59. 杨明胜.自拟豁痰通瘀泻火汤治疗中风后失眠症36例[J].中医杂志,2010,51(S2):191.DOI:10.13288/j.11-2166/r.2010.s2.072. 60. 李宁,李佳,赵雨,王成伟.“枕中丸”治疗中风后睡眠呼吸暂停综合征的临床疗效观察[J].中医药学报,2010,38(01):63-66.DOI:10.19664/j.cnki.1002-2392.2010.01.026. 61. 彭金维. 针药结合治疗中风后失眠的临床观察[D].广州中医药大学,2010. 62. 葛宝芬,禚丽梅,李敏.针刺治疗脑卒中患者并发睡眠倒错[J].山东中医杂志,2010,29(02):114.DOI:10.16295/j.cnki.0257-358x.2010.02.020. 63. 曹金明.针刺治疗脑卒中后遗症期并睡眠呼吸暂停综合征[J].中国当代医药,2010,17(22):133+136. 64. 陈兴奎.针刺治疗脑卒中后日间过度倦睡症疗效观察[J].上海针灸杂志,2010,29(09):565-566. 65. 叶永铭,李佳,王寅,等. 针刺治疗脑卒中合并睡眠呼吸暂停综合征的研究[J]. 现代中西医结合杂志,2010(1). DOI:10.3969/j.issn.1008-8849.2010.01.002. 66. 李琛. 针刺结合耳穴治疗脑梗死后失眠36例临床疗效观察[J]. 中医临床研究,2010(21). DOI:10.3969/j.issn.1674-7860.2010.21.024. 67. 贾瑞芝.针刺改善中风后患者失眠状态的临床研究[J].光明中医,2010,25(11):2070-2071. 68. 叶仿武,徐亚林,陈俊伟,谢柱明,吴月意.针刺百会穴治疗脑卒中后失眠症30例临床观察[J].实用中西医结合临床,2010,10(05):21-22. 69. 李泽锋,梁俊迪,何艳,刘秀萍,曾瑛.养血清脑颗粒对慢性脑供血不足患者失眠和认知功能改善作用的临床评价[J].中国医药指南,2010,8(22):30-32.DOI:10.15912/j.cnki.gocm.2010.22.006. 70. 桑丽清.心包经拍打结合耳穴压豆治疗脑卒中后睡眠障碍40例[J].浙江中医杂志,2010,45(05):365. 71. 王震华,黄合,金兰英.稳心颗粒治疗脑卒中后睡眠障碍66例临床观察[J].中国民族民间医药,2010,19(02):96-98. 72. 江素芹. 稳心颗粒治疗脑卒中后睡眠障碍58例临床观察[J]. 中国中医药咨讯,2010(12). 73. 赵莹雪.王松龄教授治疗脑梗死并存睡眠呼吸暂停综合征的经验[J].中医学报,2010,25(01):46-47.DOI:10.16368/j.issn.1674-8999.2010.01.013. 74. 胡丹丽.腕踝针配合艾灸涌泉穴治疗中风后失眠48例[J].白求恩军医学院学报,2010,8(06):427-428.DOI:10.16485/j.issn.2095-7858.2010.06.008. 75. 王俊,宣兵,王建,向阳.天眩清治疗脑卒中后睡眠障碍患者80例疗效评估[J].西南军医,2010,12(04):724-725. 76. 李健.黄芪注射液治疗心脑血管病合并失眠症的疗效观察[J].中国实用医药,2010,5(13):155-156.DOI:10.14163/j.cnki.11-5547/r.2010.13.011. 77. 王翠玲,徐凌忠.共鸣火花穴位刺激配合护理干预对老年脑卒中患者睡眠质量的影响[J].中华物理医学与康复杂志,2010(10):758-759. 78. 王东霞,孙丽萍,刘耀东. 丁苯酞胶囊和天麻素注射液治疗中风后失眠疗效观察[J]. 中外健康文摘,2010(22). DOI:10.3969/j.issn.1672-5085.2010.22.003. 79. 高振忠,宋立公.柴胡龙牡胶囊治疗急性中风后失眠[J].中医临床研究,2020,12(03):61. 80. 黄李双,老膺荣,黄红.辨证调护治疗脑卒中后睡眠障碍139例疗效观察[J].新中医,2010,42(08):89-90.DOI:10.13457/j.cnki.jncm.2010.08.052. 81. 陈克知.百乐眠胶囊治疗卒中后失眠42例[J].湖南中医杂志,2010,26(01):45-46.DOI:10.16808/j.cnki.issn1003-7705.2010.01.026. 82. 刘健红.自拟滋阴安神汤治疗脑卒中后失眠的临床观察[J].四川中医,2011,29(07):81-82. 83. 郭铁,郭洪明. 中医药治疗中风后失眠[J]. 中国中医急症,2011(12). DOI:10.3969/j.issn.1004-745X.2011.12.047. 84. 黄建民,王凯华.针药合用治疗脑卒中后失眠30例[J].陕西中医,2011,32(09):1227-1228. 85. 吕颖. 针罐并用治疗脑卒中后睡眠障碍的临床研究[D].成都中医药大学,2011. 86. 王寅,耿慧瑶,叶永铭,李佳.针刺治疗脑卒中合并睡眠呼吸暂停低通气综合征随访分析[J].上海针灸杂志,2011,30(04):220-222. 87. 孙远征,夏昆鹏.针刺配合耳穴贴压治疗中风后失眠临床观察[J].上海针灸杂志,2011,30(06):363-365. 88. 夏昆鹏. 针刺配合耳穴贴压治疗中风后失眠的临床研究[D]. 2011. 89. 徐琰. 益肾活血法治疗缺血性脑卒中后失眠（肾阴虚兼血瘀型）的临床观察[D].黑龙江中医药大学,2011. 90. 董学锋,畅亦杰.养血清脑颗粒治疗脑卒中后睡眠障碍患者71例疗效评估[J].中国中药杂志,2011,36(07):949-950. 91. 刘福兴. 血府逐瘀汤加减治疗血瘀型中风后不寐30例的临床观察[D]. 2011. 92. 韩永强,顾莉君,刘锦.天王补心丸治疗脑卒中后失眠症60例疗效观察[J].中国实用医药,2011,6(21):185.DOI:10.14163/j.cnki.11-5547/r.2011.21.018. 93. 莫颖敏,韩敏,韦春英,杨彭,张翼.松龄血脉康对急性脑梗死患者神经功能康复和睡眠障碍的影响[J].中西医结合心脑血管病杂志,2011,9(04):431-432. 94. 李东晓,曹晓岚. 浅析温胆汤加减治疗中风后失眠[J]. 健康必读（中旬刊）,2011(11). 95. 张朝贵.固本健脑液治疗脑卒中伴失眠症临床观察[J].四川中医,2011,29(02):82-83. 96. 王利洪. 耳穴治疗脑卒中后失眠的疗效观察[J]. 实用心脑肺血管病杂志,2011(7). DOI:10.3969/j.issn.1008-5971.2011.07.105. 97. 王海霞.耳穴压豆治疗脑卒中患者失眠90例临床观察[J].中医药临床杂志,2011,23(12):1072-1073.DOI:10.16448/j.cjtcm.2011.12.018. 98. 王瑜.“督原同调”法针刺治疗中风后失眠的临床观察[J].广西中医药,2011,34(03):22-23. 99. 叶仿武,徐亚林,陈俊伟,谢柱明,吴月意.调督针法对匹茨堡睡眠质量指数的影响[J].甘肃中医,2011,24(01):36-38. 100. 张晓燕,王升旭.子午流注纳甲法治疗脑卒中后睡眠倒错的临床观察[J].针灸临床杂志,2012,28(01):7-9. 101. 王梅,季寒梅,王淑霞. 中医整体护理对中风后失眠症状的干预[J]. 中外健康文摘,2012(30). DOI:10.3969/j.issn.1672-5085.2012.30.344. 102. 许佳平,李锐朋.中药新乐康治疗脑卒中后失眠症的临床观察[J].内蒙古中医药,2012,31(22):23-24.DOI:10.16040/j.cnki.cn15-1101.2012.22.195. 103. 黄梅,高淑红.针灸治疗对脑卒中后睡眠障碍患者的效果观察[J].针灸临床杂志,2012,28(10):15-16. 104. 丁友英. 针灸配合耳穴压豆治疗卒中后不寐的疗效观察[J]. 中国医药实践杂志,2012(5). 105. 王贯民,赵会山.针刺治疗中风后失眠32例临床观察[J].山西中医,2012,28(05):33. 106. 张林华.针刺治疗脑卒中合并睡眠呼吸暂停综合征的疗效分析[J].中国实用医药,2012,7(01):83-84.DOI:10.14163/j.cnki.11-5547/r.2012.01.018. 107. 张为民.针刺治疗对脑卒中后失眠的影响[J].中国保健营养（中旬刊）,2012,(z2):239-240. 108. 李刚,张义,罗亨勤.针刺联合艾司唑仑治疗脑卒中后失眠的疗效观察[J].西南国防医药,2012,22(06):64 109. 李琛.针刺结合耳穴贴压治疗脑梗死后失眠42例[J].河南中医,2012,32(08):1065-1066.DOI:10.16367/j.iss李琛.针刺结合耳穴贴压治疗脑梗死后失眠42例[J].河南中医,2012,32(08):1065-1066.DOI:10.16367/j.issn.1003-5028.2012.08.002.n.1003-5028.2012.08.002.1-642. 110. 徐亚林,陈俊伟,叶仿武. 针刺百会穴为主的调督法治疗脑卒中后失眠症的临床研究[J]. 按摩与康复医学（中旬刊）,2012(11). 111. 陈晓军,方针,陈利芳,杨丹红,李霞.针刺“不寐四穴”为主治疗中风后失眠临床疗效评价[J].上海针灸杂志,2012,31(12):864-866. 112. 周峻,俞桔.养心方治疗脑卒中后睡眠障碍60例[J].山西中医,2012,28(05):20-21. 113. 郎显兵,谭曦,杜丽红,杜渐,孔军辉.心理疏导配合针刺治疗脑卒中患者失眠临床研究[J].吉林中医药,2012,32(10):1064-1065.DOI:10.13463/j.cnki.jlzyy.2012.10.032. 114. 程云帆,蒋小玲,毛琳.天麻钩藤饮加味配合艾司唑仑治疗肝阳上亢型脑卒中后睡眠障碍的临床观察[J].中西医结合心脑血管病杂志,2012,10(02):178-179. 115. 关运祥.酸枣仁汤加减治疗脑卒中后失眠30例[J].中医临床研究,2012,4(19):97-98. 116. 龙芙蓉,王芳,包尚懿,罗珊,刘影.情志护理对脑梗死急性期患者焦虑和睡眠障碍的影响[J].四川中医,2012,30(06):121-123. 117. 魏正林. “靳三针”治疗缺血性脑卒中急性期睡眠障碍的临床研究[D]. 2012. 118. 卢婉敏,廖少容. 活血散穴位贴敷疗法治疗中风伴失眠患者的护理体会[J]. 健康必读（中旬刊）,2012(5). 119. 褚爱华.黄连阿胶汤加减辅以耳穴贴敷治疗卒中后失眠症84例临床观察研究[J].中医临床研究,2012,4(07):70-71. 120. 单红梅,金华锋,王凤英.化痰解郁安神汤治疗脑卒中睡眠障碍临床观察[J].山东中医杂志,2012,31(03):164-165.DOI:10.16295/j.cnki.0257-358x.2012.03.029. 121. 蔡萍,潘乃林,万瑜晔.放松训练及足部穴位按摩在脑卒中睡眠障碍病人护理的应用[J].护理研究,2012,26(04):329-331. 122. 吴雪兰,陈琴,刘从秀.耳针辨证治疗脑卒中后失眠40例[J].安徽中医学院学报,2012,31(05):45-46. 123. 吴国英,方凌云.耳穴埋豆改善中风患者失眠的效果分析[J].现代医药卫生,2012,28(22):3485-3486. 124. 邹生燕.辨证护理结合耳穴贴压治疗中风恢复期心脾两虚型失眠临床观察[J].中国临床医生,2012,40(08):55-57. 125. 张伟.艾灸结合放松疗法在中风后失眠患者中的应用研究[J].黑龙江中医药,2012,41(04):38-40. 126. 蔡军红,彭海燕,吴碧梅,卢艳芳,张宇霞.自拟沐足方改善中风后失眠患者睡眠质量与舒乐安定等效性随机平行对照研究[J].实用中医内科杂志,2013,27(08):52-54. 127. 李玉杭,秦俊岭,李娜,王民升,齐凤燕,贺心良.逐瘀安神方治疗脑卒中后失眠68例[J].中西医结合心脑血管病杂志,2013,11(07):828-829. 128. 林红霞,陈汝文,钟志国.中药足浴联合穴位按摩治疗中风后失眠的效果观察[J].中华现代护理杂志,2013(07):805-807. 129. 蔡军红,张宇霞,吴碧梅,彭海燕,卢艳芳.中药沐足对改善中风后失眠患者睡眠质量疗效观察[J].广州医学院学报,2013,41(03):115-117. 130. 冉晓.中西医结合治疗脑卒中后失眠症的疗效观察[J].航空航天医学杂志,2013,24(08):899-901. 131. 叶翠河,欧彩娣,徐祖静,等. 针刺治疗脑卒中后失眠的临床观察[J]. 中国临床新医学,2013(7). DOI:10.3969/j.issn.1674-3806.2013.07.18. 132. 邢航. 针刺十二原穴治疗中风后失眠症的临床疗效观察[D].广州中医药大学,2013. 133. 王云,丁兆生.针刺结合音乐疗法治疗脑卒中后失眠的疗效观察[J].世界中西医结合杂志,2013,8(11):1136-1138.DOI:10.13935/j.cnki.sjzx.2013.11.012. 134. 孙远征,于佳妮.原络配穴埋线治疗中风后心肾不交型失眠40例[J].针灸临床杂志,2013,29(01):33-35. 135. 赵曙光,彭瑞君,王爱珍.浴足加按摩对老年脑卒中后睡眠障碍患者的影响[J].光明中医,2013,28(07):1405-1406. 136. 孔显坤. 涌泉敷贴配合耳穴贴压治疗卒中后失眠的疗效观察[D]. 2013. 137. 庄宝平,史梅.循证护理在脑卒中患者睡眠障碍中的应用[J].内蒙古中医药,2013,32(33):158.DOI:10.16040/j.cnki.cn15-1101.2013.33.116. 138. 郭金颖.血府逐瘀汤合腹针治疗中风后失眠30例[J].浙江中医杂志,2013,48(09):650. 139. 张新玉. 穴位综合疗法治疗中风后不寐的随机对照研究[J]. 健康必读（下旬刊）,2013(2). 140. 许幸仪,陈秀慧,刘健红,黄坚红.穴位贴敷治疗风火上扰型脑梗死后失眠30例[J].河南中医,2013,33(10):1657-1658.DOI:10.16367/j.issn.1003-5028.2013.10.026. 141. 孙远征,杨圆圆.穴位埋线与耳穴贴压治疗中风后心脾两虚型失眠临床对照研究[J].中国针灸,2013,33(S1):1-4.DOI:10.13703/j.0255-2930.2013.s1.001. 142. 李霞,卫星.穴位按压合松弛疗法治疗脑卒中睡眠障碍31例[J].安徽中医学院学报,2013,32(04):58-60. 143. 王瑞娇,王玲.醒脑开窍针刺法治疗中风后睡眠倒错46例[J].河南中医,2013,33(02):260-261.DOI:10.16367/j.issn.1003-5028.2013.02.041. 144. 伍秀珍.心理干预联合针刺百会穴治疗脑卒中后失眠症[J].护理实践与研究,2013,10(07):119-120. 145. 王毅蓉,赵永刚.乌灵胶囊联合中药沐足治疗中风后失眠30例疗效观察[J].湖南中医杂志,2013,29(12):53-54.DOI:10.16808/j.cnki.issn1003-7705.2013.12.026. 146. 孙利华. 王不留行籽耳穴压豆改善脑血管意外患者睡眠障碍的临床观察[D].浙江大学,2013. 147. 王乃梅.帕罗西汀联合百乐眠治疗脑卒中睡眠障碍的临床观察[J].中国医药导刊,2013,15(06):992+994. 148. 孙利华,李艳娟,付根莲,金静芬,付文娟,袁小红.脑血管意外睡眠障碍患者的中医辨证施护[J].护理与康复,2013,12(05):467-468. 149. 毕钰桢,何绪屏,张大创,燕竹青.“眠三针”治疗中风后睡眠障碍20例[J].广西中医药,2013,36(06):25-26. 150. 贾志明. 加味归脾汤加穴位敷贴治疗缺血性中风后失眠[D].湖北中医药大学,2013. 151. 姜美华.活血散穴位贴敷联合常规护理治疗中风伴失眠随机平行对照研究[J].实用中医内科杂志,2013,27(19):21-22. 152. 赵莹雪.化痰熄风逐瘀汤治疗脑梗死合并阻塞性睡眠呼吸暂停低通气综合征临床研究[J].中医学报,2013,28(11):1729-1730.DOI:10.16368/j.issn.1674-8999.2013.11.028. 153. 陈志慧.耳穴压豆治疗中风患者失眠疗效的观察与护理[J].内蒙古中医药,2013,32(36):84.DOI:10.16040/j.cnki.cn15-1101.2013.36.041. 154. 王超,刘湖水. 耳穴压豆在老年中风失眠患者中的应用体会[J]. 中国保健营养（中旬刊）,2013(8). 155. 李蕾蕾,刘芝修,刘静,陈桂兰,伍梅梅,甄淑敏,黄秀娟.耳穴埋豆疗法对卒中后抑郁患者失眠的影响[J].中医药临床杂志,2013,25(11):1001-1002.DOI:10.16448/j.cjtcm.2013.11.013. 156. 吴毅军,于冬冬,陈好远.电针配耳穴治疗中风后失眠临床研究[J].中医学报,2013,28(06):913-914.DOI:10.16368/j.issn.1674-8999.2013.06.006. 157. 宿成君,任瑞梅,宋立公.柴胡龙牡丸治疗缺血性中风后失眠[J].光明中医,2013,28(05):943-944. 158. 白晓花,马凤. 辩证护理结合耳穴贴压在中风恢复期失眠治疗中的临床价值[J]. 医学信息,2013(11). DOI:10.3969/j.issn.1006-1959.2013.11.367. 159. 程淑贤.砭石足部反射区按摩治疗中风后失眠的临床观察[J].光明中医,2013,28(01):127-128. 160. Wang Yin,Geng Hui-yao,Ye Yong-ming,Li Jia,Huang Guo-qi.Observation on Clinical Effects of Electroacupuncture Therapy for Apoplexy with Obstructive Sleep Apnea Syndrome[J].Journal of Acupuncture and Tuina Science,2013,11(06):342-345. 161. 唐旭丽.56例脑卒中睡眠障碍的中医护理体会[J].中国民族民间医药,2013,22(15):145+147. 162. 康群,储小红,虞华,戴其军.足部反射区按摩对脑卒中后失眠患者睡眠质量的影响[J].中华现代护理杂志,2014,20(08):872-874. 163. 秦小怡,戴晓英,方婷霞,洪涛.自拟中草药汤剂改善脑卒中后失眠的临床观察[J].中国中医药科技,2014,21(05):573-574. 164. 邵子杰.中医辨证施治结合耳穴压豆治疗中风后失眠临床疗效观察[J].亚太传统医药,2014,10(12):85-86. 165. 东梅.中药足浴配合穴位按摩在中风睡眠障碍患者中的应用研究[J].当代护士(下旬刊),2014(03):109-111. 166. 黄丽晴,葛芳.中药足浴联合康复护理干预对中风后抑郁性失眠患者日常生活能力的临床观察[J].辽宁中医杂志,2014,41(06):1197-1198.DOI:10.13192/j.issn.1000-1719.2014.06.055. 167. 陈明菊,米莉,杨金华.中药足浴联合康复护理干预对中风后抑郁性失眠患者ADL能力的临床观察[J].辽宁中医杂志,2014,41(08):1729-1730.DOI:10.13192/j.issn.1000-1719.2014.08.074. 168. 程耀南,吴洁.中药药氧疗法治疗脑卒中后失眠症的临床疗效评价分析[J].成都中医药大学学报,2014,37(02):72-75.DOI:10.13593/j.cnki.51-1501/r.2014.02.072. 169. 张俊杰,曾科学,龚雪. 中药熏洗配合针刺治疗脑卒中恢复期失眠疗效观察[J]. 按摩与康复医学,2014(7). 170. 孙学平.中西医结合治疗脑卒中睡眠障碍疗效观察60例[J].中国社区医师,2014,30(03):72+74. 171. 董松巧.中西医结合治疗脑卒中患者睡眠障碍的对照研究[J].中国当代医药,2014,21(07):121-123. 172. 傅俊杰,杨洪波.中西医结合治疗脑卒中后睡眠障碍临床观察[J].医药论坛杂志,2014,35(05):168-169. 173. 赵宇,王顺. 针灸治疗脑中风后失眠[J]. 世界最新医学信息文摘（连续型电子期刊）,2014(30). DOI:10.3969/j.issn.1671-3141.2014.30.236. 174. 朱晨. 针刺治疗中风后失眠临床观察[J]. 广西中医药,2014(6). 175. 吕晓华,张海军. 针刺治疗脑卒中后抑郁失眠[J]. 医学美学美容（中旬刊）,2014(4). 176. 尤璐. 针刺治疗脑梗死后失眠的临床观察[D]. 北京中医药大学, 2014. 177. 郑健刚,杨红玲. 针刺治疗急性脑卒中后睡眠障碍100例临床疗效观察[C]//中国针灸学会针灸临床服务模式经验研讨暨第十一届全国中青年针灸推拿学术交流会论文集.[出版者不详],2014:212-218. 178. 王岫兰,杭燕,康群.涌泉穴按摩对中风患者睡眠障碍的负性情绪及睡眠质量影响[J].国际护理学杂志,2014,33(12):3621-3622. 179. 范寒院,周梅君,梁伟容.音乐电针疗法在脑卒中后睡眠障碍患者中的应用效果分析[J].临床医学工程,2014,21(10):1309-1310. 180. 陈坤,许彩梅,陈萍. 养血清脑颗粒治疗CADASIL的疗效观察[J]. 内蒙古医学杂志,2014(11). 181. 张素华.养心方对脑卒中后睡眠障碍的治疗效果分析[J].中国医药指南,2014,12(08):164-165.DOI:10.15912/j.cnki.gocm.2014.08.014. 182. 林云志. 穴位敷贴对中风后失眠的临床观察[D]. 2014. 183. 杨学军,袁盈,胡鹏,等. 穴位按压改善脑梗死后并发失眠的护理研究[J]. 中医学报,2014(0). 184. 丘艳红,曾科学. 五音特色护理干预脑卒中后睡眠障碍疗效观察[J]. 按摩与康复医学,2014(8). 185. 李文武.乌灵胶囊治疗脑梗死伴焦虑及睡眠障碍76例疗效观察[J].云南中医中药杂志,2014,35(12):28-29.DOI:10.16254/j.cnki.53-1120/r.2014.12.009. 186. 刘俊琼.乌灵胶囊改善卒中后抑郁患者睡眠障碍临床疗效观察[J].中医临床研究,2014,6(36):37-38. 187. 丁翔.温胆汤加味治疗脑卒中后抑郁失眠30例[J].云南中医中药杂志,2014,35(02):45.DOI:10.16254/j.cnki.53-1120/r.2014.02.017. 188. 张鸽. 熟眠贴对缺血性中风后恢复期失眠病人疗效的临床研究[D].长春中医药大学,2014. 189. 陈曦光. 失眠方贴敷涌泉穴对脑卒中后失眠症的影响[D].广州中医药大学,2014. 190. 焦久存.三黄安神汤对脑梗塞后痰热内扰型失眠症疗效的影响[J].河北中医药学报,2014,29(04):20-22.DOI:10.16370/j.cnki.13-1214/r.2014.04.007. 191. 卢慧清.脑功能治疗仪联合耳穴压豆治疗老年失眠患者的临床观察[J].中医临床研究,2014,6(04):63-64. 192. 刘芳,李中元,陈焰南,陈谷兰.六字诀对中风后睡眠障碍的影响[J].河北联合大学学报(医学版),2014,16(01):77-78.DOI:10.19539/j.cnki.2095-2694.2014.01.054. 193. 潘微.李智杰教授治疗脑梗死后失眠的经验[J].光明中医,2014,29(01):40-41. 194. 庄礼兴,江烨.靳三针疗法治疗脑卒中后睡眠障碍的临床疗效研究[J].广州中医药大学学报,2014,31(04):553-557.DOI:10.13359/j.cnki.gzxbtcm.2014.04.014. 195. 江烨. 靳三针疗法治疗脑卒中后失眠的临床研究[D].广州中医药大学,2014. 196. 李红,韦秋连.金匮肾气丸联合艾司唑仑治疗脑卒中后失眠随机平行对照研究[J].实用中医内科杂志,2014,28(06):120-122.DOI:10.13729/j.issn.1671-7813.2014.06.58. 197. 张梓岗.加味四物颗粒治疗脑梗死后睡眠障碍[J].长春中医药大学学报,2014,30(05):886-887.DOI:10.13463/j.cnki.cczyy.2014.05.048. 198. 王友静.耳穴压籽对中风患者睡眠状况的影响[J].当代护士(下旬刊),2014(08):100-101. 199. 丁红梅.耳穴压豆配合隔姜灸心俞穴治护脑卒中后睡眠障碍临床观察[J].中医药临床杂志,2014,26(03):227-229.DOI:10.16448/j.cjtcm.2014.03.028. 200. 邢雨胜.耳穴贴压治疗中风后失眠32例[J].保健医学研究与实践,2014,11(02):41-43. 201. 张伟.耳穴配合体针治疗中风后失眠[J].内蒙古中医药,2014,33(34):73.DOI:10.16040/j.cnki.cn15-1101.2014.34.074. 202. 陈晓娜,黄小丽,廖莹莹,李家华,曾科学.耳穴埋豆配合五音疗法治疗中风后失眠33例疗效观察[J].云南中医中药杂志,2014,35(01):55-56.DOI:10.16254/j.cnki.53-1120/r.2014.01.026. 203. 刘婉玲. 耳尖放血结合电针治疗中风后失眠疗效观察[D].广州中医药大学,2014. 204. 缑燕华,杨卓欣. 调任通督针法治疗脑卒中后失眠症60例的临床观察[J]. 医药前沿,2014(16). DOI:10.3969/j.issn.2095-1752.2014.16.389. 205. 唐雷,游菲,马朝阳.低频电穴位刺激治疗脑卒中后失眠疗效观察[J].中国针灸,2014,34(08):747-750.DOI:10.13703/j.0255-2930.2014.08.010. 206. 宋芷珩,孟季红,孟丽丹.川芎清脑颗粒治疗脑梗死后头痛、头晕伴失眠的研究[J].中国综合临床,2014,30(03):251-252. 207. 奎瑜,劳玲珠,李漾,郑德采,杨伟毅,杨仁轩,何羿婷,郭程湘.畅气通络针法治疗卒中后睡眠障碍的疗效评价[J].广东医学,2014,35(04):601-603.DOI:10.13820/j.cnki.gdyx.2014.04.048. 208. 袁映梅,丘伟忠.艾灸治疗老年性卒中后失眠的有效护理模式分析[J].内蒙古中医药,2014,33(22):167-169.DOI:10.16040/j.cnki.cn15-1101.2014.22.124. 209. 仲跻凤.TCD联合颈动脉彩色超声检测脑卒中失眠患者加用足部反射区按摩后的疗效观察[J].南通大学学报(医学版),2014,34(05):400-402. 210. 洪秀瑜,张卫.卒中后睡眠障碍应用畅气通络针法治疗的效果观察[J].中国医药指南,2015,13(30):190-191.DOI:10.15912/j.cnki.gocm.2015.30.152. 211. 陈婷婷,葛芳,楚佳梅.足疗联合心理疗法治疗脑卒中后抑郁性失眠30例临床研究[J].江苏中医药,2015,47(01):37-38. 212. 储小红,康群,杭燕.足部穴位按摩在脑卒中后病人睡眠障碍中的应用[J].护理研究,2015,29(32):4041-4043. 213. 韩旭翠. 子午流注纳子法针刺治疗脑卒中后睡眠障碍的临床研究[D].广州中医药大学,2015. 214. 王泽华,杨其华,邱菊. 中医综合护理干预对脑卒中患者睡眠障碍的影响[J]. 四川中医,2015(8). 215. 曾令烽,邹元平,黄小桃,宓穗卿,孔令朔,王奇,王宁生.中医药干预对改善脑卒中后睡眠障碍疗效与安全性的系统评价[J].中华中医药杂志,2015,30(05):1488-1498. 216. 田信,田琼.中医护理干预对脑血管意外患者睡眠障碍的影响[J].世界最新医学信息文摘,2019,19(36):275+284.DOI:10.19613/j.cnki.1671-3141.2019.36.192. 217. 靳国民,郭静.中医辨证施护配合耳穴贴压对缺血性卒中后失眠的影响[J].河北中医,2015,37(04):600-602. 218. 唐旭丽,邓旭,钟毅,覃秋菊,韦兰英.中药浴足联合隔姜灸对脑卒中睡眠障碍的护理干预研究[J].中医外治杂志,2015,24(04):8-10. 219. 连小勤. 中药药枕对缺血性脑卒中失眠患者睡眠质量的影响[D].福建中医药大学,2015. 220. 吴洁,程耀南.中药药氧疗法佐治脑卒中恢复期患者失眠40例[J].中医外治杂志,2015,24(04):26-27. 221. 葛云霞,陶玉华.中药熏洗配合耳穴埋籽干预脑梗死后失眠50例效果观察[J].湖南中医杂志,2015,31(10):107-108.DOI:10.16808/j.cnki.issn1003-7705.2015.10.064. 222. 刘志远.中药三黄安神汤治疗脑梗死后痰热内扰型失眠症疗效观察[J].实用中西医结合临床,2015,15(12):66-67.DOI:10.13638/j.issn.1671-4040.2015.12.036. 223. 王俊霞.中西医护理结合穴位中频脉冲对脑卒中失眠的效果[J].中国民间疗法,2015,23(02):87.DOI:10.19621/j.cnki.11-3555/r.2015.02.073. 224. 梁晓雯. 针剌治疗中风后失眠的Meta分析及选穴规律研究[D].黑龙江中医药大学,2015. 225. 阮班魁.针灸和药物治疗脑卒中后睡眠障碍的效果对比[J].中医临床研究,2015,7(19):31-32. 226. 王赟芝.针刺治疗急性脑梗死合并阻塞性睡眠呼吸暂停低通气综合征疗效观察[J].上海针灸杂志,2015,34(11):1027-1030.DOI:10.13460/j.issn.1005-0957.2015.11.1027. 227. 张怀艺. 针刺配合耳穴压籽治疗中风后失眠的临床研究[D].成都中医药大学,2015. 228. 杨静. 针刺配合耳穴压籽治疗中风后失眠的临床研究[D].成都中医药大学,2015. 229. 李文颢. 针刺结合择时选穴灸法干预中风后失眠的临床观察[D].广州中医药大学,2015. 230. 汤宇,张松兴.针刺“三神穴”为主治疗脑卒中后失眠的临床观察[J].中西医结合心脑血管病杂志,2015,13(16):1885-1887. 231. 汪雪菁.右佐匹克隆联合九味镇心颗粒治疗脑卒中后焦虑、失眠的临床观察[J].中国处方药,2015,13(07):43-44. 232. 岳冬敏.养血清脑颗粒联合舍曲林治疗脑卒中睡眠障碍的临床疗效[J].湖北中医药大学学报,2015,17(06):62-64. 233. 刘红. 养血清脑颗粒联合氟桂利嗪胶囊对慢性脑供血不足患者认知功能障碍和睡眠障碍的影响[J]. 检验医学与临床,2015(z2). DOI:10.3969/j.issn.1672-9455.2015.26.009. 234. 周媛,秦远文.养心安神汤治疗脑卒中后失眠临床观察[J].辽宁中医药大学学报,2015,17(08):187-188.DOI:10.13194/j.issn.1673-842x.2015.08.064. 235. 张志山.穴位埋线治疗脑卒中恢复期失眠症临床研究[J].亚太传统医药,2015,11(18):80-81. 236. 王文熠,王锋.醒脑开窍针刺法结合灵龟八法治疗脑卒中后昼间嗜睡[J].吉林中医药,2015,35(11):1176-1179.DOI:10.13463/j.cnki.jlzyy.2015.11.029. 237. 郭佳莹,徐燕,欧秀娟,何敏玲.新型家庭康复计划对中经络型中风病失眠患者睡眠质量及生存质量的影响[J].广州中医药大学学报,2015,32(05):808-812.DOI:10.13359/j.cnki.gzxbtcm.2015.05.006. 238. 杨金亮,张蓉,杨云霜,瞿彬,杜磊.心脑宁胶囊治疗脑卒中后失眠的临床疗效观察[J].北京医学,2015,37(01):100-102.DOI:10.15932/j.0253-9713.2015.1.040. 239. 何洁,何安琪,王筱蓊.温泉浸浴配合耳穴贴压治疗缺血性脑卒中患者失眠的护理体会[J].中西医结合护理(中英文),2015,1(02):4-6. 240. 周荣. 天王补心汤治疗缺血性脑卒中后失眠（阴虚火旺型）的临床疗效评价[D].浙江中医药大学,2015. 241. 焦久存.清热安神汤联合失眠三针治疗脑梗死后痰热内扰型失眠症80例临床观察[J].河北中医,2015,37(01):44-45. 242. 蔡南哨,于海波,李志峰,詹晓惠.脑卒中后睡眠障碍的中西医护理体会[J].当代护士(中旬刊),2015(01):85-87. 243. 刘慧.脑血管意外患者睡眠障碍的中医护理观察[J].内蒙古中医药,2015,34(03):121-122.DOI:10.16040/j.cnki.cn15-1101.2015.03.127. 244. 吴洁,徐勇.健脾化痰活血针法配合认知行为疗法治疗脑卒中后失眠临床观察[J].新中医,2015,47(07):238-240.DOI:10.13457/j.cnki.jncm.2015.07.106. 245. 王伟民,张明明.化痰通窍熄风方联合西药治疗动脉硬化性脑梗死合并阻塞性睡眠呼吸暂停低通气综合征30例[J].中医研究,2015,28(11):22-24. 246. 李超华. 腹针治疗中风后失眠的临床观察[D].辽宁中医药大学,2015. 247. 张霞,张新生,谢燕. 耳穴压丸结合足底按摩/穴位贴敷治疗卒中后睡眠障碍的临床观察[J]. 中国中医急症,2015(2). DOI:10.3969/j.issn.1004-745X.2015.02.041. 248. 梁冰莲,谭锦秀.耳穴压豆配合贴敷涌泉穴治疗脑卒中病人失眠的效果观察[J].循证护理,2015,1(02):86-88. 249. 姜正荣,蒋玲.耳穴压豆联合中药足浴治疗脑卒中失眠的效果观察[J].内蒙古中医药,2015,34(09):63.DOI:10.16040/j.cnki.cn15-1101.2015.09.076. 250. 杜何欣,李毓娇.耳穴压豆结合情志护理对脑卒中后失眠的临床观察[J].光明中医,2015,30(12):2611-2612. 251. 蔡晓敏,章旭萍,唐杏,黄国琪.耳穴贴压结合音乐疗法对脑卒中后失眠的疗效观察(英文)[J].Journal of Acupuncture and Tuina Science,2015,13(04):227-231. 252. 尹春月,任毅,张勇.耳穴贴压改善脑卒中后失眠患者睡眠状况的临床观察[J].现代中西医结合杂志,2015,24(07):774-775. 253. 陈俊玲,吴晗芳,邵一萍,泮洁.耳穴埋豆联合中药改善脑卒中后抑郁患者失眠的疗效观察[J].辽宁中医杂志,2015,42(05):1025-1027.DOI:10.13192/j.issn.1000-1719.2015.05.046. 254. 杨华琴. 督脉隔姜蒜灸对脑梗死后失眠疗效及神经功能康复的影响[D].湖南中医药大学,2015. 255. 吴钊.定痫丸加减治疗脑梗死失眠36例[J].光明中医,2015,30(01):74-75. 256. 李陈渝,杨健.“调阴阳五脏”针法治疗卒中后睡眠障碍的临床研究[J].中西医结合心脑血管病杂志,2015,13(01):41-43. 257. 邹治宏,于海波,杨卓欣.调任通督法与传统针法治疗中风后失眠的临床疗效比较[J].广州医科大学学报,2015,43(03):110-113. 258. 缑燕华,杨卓欣.调任通督法治疗中风后失眠症临床观察[J].上海针灸杂志,2015,34(06):505-507.DOI:10.13460/j.issn.1005-0957.2015.06.0505. 259. 李筱媛,郝媌.电针联合推拿治疗卒中后睡眠障碍160例疗效观察[J].中西医结合心脑血管病杂志,2015,13(14):1681-1683. 260. 冉玲丽. 低频电穴位刺激影响脑卒中后失眠患者血浆5-HT、DA浓度的临床研究[D].湖北中医药大学,2015. 261. 唐雷,马朝阳,游菲,丁琳.低频电穴位刺激对脑卒中后失眠患者血浆5-HT及NE的影响[J].中国针灸,2015,35(08):763-767.DOI:10.13703/j.0255-2930.2015.08.004. 262. 庞芳,杨志宏,问莉娜.从痰论治中风后失眠[J].山东中医杂志,2015,34(09):716-717.DOI:10.16295/j.cnki.0257-358x.2015.09.030. 263. 郑丽.柴胡加龙骨牡蛎汤加味治疗缺血性中风后失眠36例临床观察[J].中医临床研究,2015,7(36):50. 264. 郑喜英,姚淑文,张群威,程志忠.参芪五味子片联合黛力新对脑卒中后失眠的影响[J].中国实用神经疾病杂志,2015,18(06):122-123. 265. 杨红玲,郑健刚.“病证结合”针刺治疗急性脑梗死后肝肾阴虚型睡眠障碍疗效观察[J].辽宁中医药大学学报,2015,17(04):182-185.DOI:10.13194/j.issn.1673-842x.2015.04.064. 266. 焦久存.艾灸联合三黄安神汤治疗痰热内扰型失眠症的疗效分析[J].陕西中医,2015,36(02):220-221. 267. 侯志涛,孙忠人,孙申田.经颅重复针刺激疗法对卒中后失眠患者血清食欲素A水平的影响[J].中国针灸,2018,38(10):1039-1042+1052.DOI:10.13703/j.0255-2930.2018.10.003. 268. 徐丰. 中医针灸治疗中风患者失眠症的临床分析[J]. 心理医生,2016(24). 269. 陈丽丽,徐玉梅,欧子杨,翁雪云.中医护理临床路径在中风后失眠患者中的应用[J].护理实践与研究,2016,13(20):141-143. 270. 马香丽. 中药足浴联合失眠贴对脑卒中睡眠障碍护理干预研究[J]. 临床研究,2016(11). 271. 陈惠玲,林红霞,陈汝文,黄益军,徐燕,钟志国.中药足浴对中风后失眠患者睡眠质量的影响[J].齐鲁护理杂志,2016,22(13):67-69. 272. 郝文文.中药沐足对脑梗死后遗失眠的护理效果[J].按摩与康复医学,2016,7(23):54-55. 273. 周美红.中药干预改善脑卒中后睡眠障碍临床观察[J].新中医,2016,48(09):16-17.DOI:10.13457/j.cnki.jncm.2016.09.009. 274. 闻涛.中西医结合治疗卒中后睡眠障碍的临床效果观察[J].河南医学研究,2016,25(07):1256-1257. 275. 王宁.中西医结合护理对脑卒中后睡眠障碍患者睡眠质量及生命质量的影响[J].中国药物经济学,2016,11(01):170-171. 276. 雷艳.中西药结合治疗脑卒中后失眠患者的疗效[J].医疗装备,2016,29(12):144-145. 277. 范小会,刘华,姬昌,范军铭.针药并用治疗卒中后失眠的临床体会[J].中医临床研究,2016,8(06):116-118. 278. 靳发万.针灸治疗脑卒中后痰热扰心型失眠30例临床疗效观察[J].世界最新医学信息文摘,2016,16(38):71-72. 279. 卢家春,白新刚,雷行华,熊兴娟,陈燕,温权,陆必波,何敏,王文春,张安仁.针灸结合运动疗法治疗老年脑卒中睡眠障碍40例疗效观察[J].康复学报,2016,26(02):17-20. 280. 任彩虹. 针刺五脏俞配合催眠疗法对卒中后睡眠障碍的疗效观察[D]. 2016. DOI:10.7666/d.Y3249919. 281. 赵京媛. 针刺配合耳穴压籽治疗中风后失眠的临床研究[D].成都中医药大学,2016. 282. 刘永锋,马晓明,闫兵,杨颖,黄杏贤,于海波.针刺配合艾灸百会穴治疗中风后失眠疗效观察[J].上海针灸杂志,2016,35(03):270-272.DOI:10.13460/j.issn.1005-0957.2016.03.0270. 283. 卢颖.针刺联合心理干预对中风后睡眠障碍患者睡眠质量的影响[J].社区医学杂志,2016,14(20):81-82. 284. 赖锋.针刺联合健康教育-心理干预治疗中风后失眠随机平行对照研究[J].实用中医内科杂志,2016,30(09):98-100.DOI:10.13729/j.issn.1671-7813.2016.09.36. 285. 宋晶晶. 针刺联合加味黄连温胆汤治疗脑梗死后痰热内扰型失眠的临床研究[D].南京中医药大学,2016. 286. 丁柏翠,赵艳玲,罗景,魏艳蓉,秦思佳.针刺董氏奇穴治疗中风后失眠30例临床观察[J].湖南中医杂志,2016,32(03):98-99.DOI:10.16808/j.cnki.issn1003-7705.2016.03.049. 287. 宁式颖,张博,于婷婷,杨玉赫,郑璐,李超.针刺“心俞三神”穴配合枣仁归脾二花汤治疗中风后失眠[J].中医药学报,2016,44(01):89-90.DOI:10.19664/j.cnki.1002-2392.2016.01.029. 288. 胡桢. 择时针刺治疗中风后睡眠倒错的临床观察[D].南京中医药大学,2016. 289. 高潇,张浩,柴剑波,赵玉萍,于明,赵永厚.愈癫汤治疗痰热型缺血性中风后不寐20例临床疗效观察[J].中华中医药杂志,2016,31(02):729-730. 290. 古柱亮. 俞募配穴针刺治疗脑卒中后阴虚火旺型失眠的临床研究[D].广州中医药大学,2016. 291. 周梅君,范寒院,李浅峰,黄智勇,梁伟容.音乐电针治疗与磁疗对脑卒中后睡眠障碍的影响[J].中国实用神经疾病杂志,2016,19(13):22-24. 292. 毛继先,方习红,杨青.穴位贴敷配合耳穴压豆对中风后抑郁患者失眠的影响[J].光明中医,2016,31(06):829-830. 293. 马湘玉,胡慧.穴位按摩配合脑电仿生刺激仪治疗脑卒中后睡眠障碍的护理干预研究[J].全科护理,2016,14(10):1005-1006. 294. 葛芳,曾友华,黄丽晴,毛美琴.温经散寒洗剂浴足联合心理疗法治疗脑卒中后睡眠障碍临床观察[J].中草药,2016,47(11):1926-1930. 295. 郑颖力.腕踝针配合艾灸涌泉穴治疗中风后失眠的临床效果观察[J].世界最新医学信息文摘,2016,16(63):178. 296. 许倩,占大权,黄飞霞.头针配合背俞穴埋线治疗中风恢复期失眠症疗效观察[J].上海针灸杂志,2016,35(02):157-159.DOI:10.13460/j.issn.1005-0957.2016.02.0157. 297. 李明月,邹伟,孙晓伟.通络安神针法治疗中风后睡眠障碍的疗效观察[J].针灸临床杂志,2016,32(04):31-33. 298. 祁霞珍,秦松杰,胡赟.通督调神针法配合中医护理对脑卒中后睡眠障碍的影响[J].西部中医药,2016,29(05):113-116. 299. 谭玉洁. 通督调神针刺治疗卒中后失眠的临床研究[D].安徽中医药大学,2016. 300. 肖东芳.甜梦口服液联合右佐匹克隆治疗脑梗死后失眠症的疗效观察[J].现代药物与临床,2016,31(10):1612-1615. 301. 李娟.舒脑欣滴丸治疗脑缺血引起失眠的疗效观察[J].中国城乡企业卫生,2016,31(11):142-143.DOI:10.16286/j.1003-5052.2016.11.059. 302. 吕昕,郭韶韶.舒肝解郁胶囊联合右佐匹克隆治疗卒中后睡眠障碍的临床研究[J].中西医结合心脑血管病杂志,2016,14(13):1543-1544+1568. 303. 闫永钇,张晓乐,韩祖成.失眠颗粒联合经颅磁刺激治疗缺血性卒中后不寐疗效观察[J].陕西中医,2016,37(08):993-994. 304. 郑聪,王清泉.祛痰安神汤治疗脑卒中后失眠的疗效观察[J].内蒙古中医药,2016,35(04):18.DOI:10.16040/j.cnki.cn15-1101.2016.04.018. 305. 胡红涛,艾艳萍.帕罗西汀联合百乐眠对脑卒中睡眠障碍患者的治疗效果及对睡眠质量的影响[J].医学综述,2016,22(20):4123-4125+4129. 306. 付丽,马朝阳,唐雷,王颖.偶刺治疗脑卒中后失眠伴抑郁临床观察[J].上海针灸杂志,2016,35(10):1184-1186.DOI:10.13460/j.issn.1005-0957.2016.10.1184. 307. 马乐.脑卒中睡眠障碍中西医结合护理效果观察[J].中国实用医药,2016,11(18):252-253.DOI:10.14163/j.cnki.11-5547/r.2016.18.171. 308. 周雅然,高旸. 论针刺治疗脑卒中后睡眠障碍-高旸教授针刺经验[J]. 心理医生,2016(32). 309. 刘强. 留针时间对针刺治疗脑梗死后失眠疗效的影响[D]. 2016. 310. 李杰萍,姜翠红,吕丽萍. 经颅磁疗联合耳穴压豆对脑卒中患者睡眠质量的影响[J]. 中外医学研究,2016(8). DOI:10.14033/j.cnki.cfmr.2016.8.052. 311. 张丽娜.加味温胆汤配合耳穴贴敷治疗脑梗塞后痰热内扰型失眠的临床观察[J].中国中医基础医学杂志,2016,22(09):1241-1242+1280. 312. 黄延超,曹晓.加味酸枣仁汤治疗脑卒中后失眠的临床观察[J].中医临床研究,2016,8(17):97-98. 313. 王敏. 加味菖蒲郁金汤治疗脑卒中后睡眠障碍（痰瘀阻窍证）的临床观察[D].湖北中医药大学,2016. 314. 张爱萍.活血安神方治疗缺血性脑卒中后失眠的临床观察[J].中医临床研究,2016,8(28):63-64+66. 315. 赵慧新,李晓芳,王楚涵,张泽明,张杏红.荷丹片联合阿托伐他汀对伴有睡眠呼吸暂停的脑梗死患者血脂及炎症因子的影响[J].中国医药导报,2016,13(03):134-136+141. 316. 赵娜,胡万华,吴志敏,吴旭杰,周荣,张慧和.归脾汤结合耳穴埋豆治疗卒中后失眠的疗效及对血清TNF-α水平的影响[J].中华中医药学刊,2016,34(12):3038-3040.DOI:10.13193/j.issn.1673-7717.2016.12.062. 317. 曹洪涛.归脾汤合中药足浴治疗中风后失眠43例[J].中国中医药现代远程教育,2016,14(24):83-84. 318. 王玮. 归脾安神汤治疗脑梗死后睡眠障碍的临床观察[J]. 心理医生,2016(24). 319. 章亚娣,周小娟.耳穴贴压治疗脑卒中恢复期患者失眠疗效分析[J].浙江中西医结合杂志,2016,26(02):133-135. 320. 梁秀莉.耳穴贴压疗法对卒中后睡眠障碍患者睡眠质量和神经功能缺损的影响[J].河北中医药学报,2016,31(02):37-39.DOI:10.16370/j.cnki.13-1214/r.2016.02.014. 321. 李贝,白姣姣,贺佩青,陈申旭.耳穴贴压结合腹式呼吸治疗老年卒中后睡眠障碍疗效观察[J].上海中医药杂志,2016,50(06):67-69.DOI:10.16305/j.1007-1334.2016.06.022. 322. 钱铃铃.耳穴埋籽对于急性脑梗死患者失眠的疗效观察[J].中国社区医师,2016,32(22):181+183. 323. 赵娜,李玮,胡万华.多导睡眠图评价归脾汤结合耳穴埋豆治疗卒中后失眠的疗效[J].浙江中医杂志,2016,51(09):625-627.DOI:10.13633/j.cnki.zjtcm.2016.09.001. 324. 刘雪影. 定神针结合腹针治疗中风后失眠症的疗效观察[D].广州中医药大学,2016. 325. 王晓华,周震,王颖.调神利眠针法联合艾司唑仑对脑梗死后失眠的影响[J].河北中医,2016,38(01):102-105. 326. 马晓明,杨卓欣,于海波,李晶晶,张少芸.调任通督针法治疗卒中后失眠的临床效果[J].中国医药导报,2016,13(11):150-154. 327. 马晓明,杨卓欣,于海波,李晶晶,闫兵,刘永锋,周鹏,张少芸.调任通督针法对卒中后失眠患者睡眠脑电图各参数的影响[J].陕西中医,2016,37(07):913-915. 328. 杨雪捷,于海波,黄杏贤,罗晓舟,黄昭志.低频电穴位刺激治疗脑卒中后失眠的临床疗效及作用机制[J].中国老年学杂志,2016,36(22):5560-5562. 329. 许芳,李侠,曲淼,吕凯,李凛,黄丽贤.从清热化痰法论治急性脑梗死后睡眠障碍[J].北京中医药,2016,35(12):1161-1163.DOI:10.16025/j.1674-1307.2016.12.017. 330. 梅绍游. 柴胡加龙骨牡蛎汤加味治疗缺血性中风后失眠的临床研究[J]. 医药前沿,2016(8). 331. 孙静.不同中医护理脑梗死后遗症患者失眠的干预效果观察[J].内蒙古中医药,2016,35(11):172.DOI:10.16040/j.cnki.cn15-1101.2016.11.161. 332. 黄阳,邱莲娜,陈海芬.不同艾灸时间对脑卒中后睡眠功能障碍影响的随机对照研究[J].山西中医,2016,32(10):19-22. 333. 吴红,施贝德.补阳还五汤加减联合耳穴贴压改善中风后睡眠障碍临床研究[J].新中医,2016,48(07):40-42.DOI:10.13457/j.cnki.jncm.2016.07.018. 334. 孟长君,孟国玮.补阳还五汤合安神定志丸治疗脑卒中后失眠20例[J].光明中医,2016,31(14):2046-2047. 335. 冯健峰. 补泻针法治疗中风后失眠的临床观察及神经递质机理研究[D].广州中医药大学,2016. 336. 郝建波,周焕娇,王云翠.“标本配穴”温针灸联合头背部推拿治疗脑梗死后失眠的疗效观察[J].中华中医药学刊,2016,34(07):1723-1726.DOI:10.13193/j.issn.1673-7717.2016.07.054. 337. 高惠艳.辨证施护对缺血性中风后失眠的疗效观察[J].光明中医,2016,31(20):3016-3018. 338. 张莺,李星凌,房连强,韩德雄,章旭萍.PSQI量表评价五行音乐结合耳穴贴压治疗脑卒中后失眠疗效[J].中华中医药杂志,2016,31(08):3063-3065. 339. Cao Y, Yin X, Soto-Aguilar F, Liu Y, Yin P, Wu J, Zhu B, Li W, Lao L, Xu S. Effect of acupuncture on insomnia following stroke: study protocol for a randomized controlled trial. Trials. 2016 Nov 16;17(1):546. doi: 10.1186/s13063-016-1670-0. PMID: 27852282; PMCID: PMC5112621. 340. Lee SH, Lim SM. Acupuncture for insomnia after stroke: a systematic review and meta-analysis. BMC Complement Altern Med. 2016 Jul 19;16:228. doi: 10.1186/s12906-016-1220-z. PMID: 27430619; PMCID: PMC4950252. 341. 王俊力. 卒中单元中西医结合治疗对卒中后抑郁合并睡眠障碍的临床研究[D].湖北中医药大学,2017. 342. 王俊力,张忠文,邵卫,魏丹,陈国华,梅俊华.卒中单元针药治疗对脑卒中后抑郁合并睡眠障碍影响的研究[J].中医药导报,2017,23(04):70-73.DOI:10.13862/j.cnki.cn43-1446/r.2017.04.022. 343. 白三晋.足疗联合心理疗法治疗脑卒中后抑郁性失眠的临床观察[J].双足与保健,2017,26(11):3-5.DOI:10.19589/j.cnki.issn1004-6569.2017.11.002. 344. 林红霞,陈惠玲,黄益军,徐燕,陈汝文.中医护理干预对中风患者负性情绪及睡眠障碍的影响[J].中医临床研究,2017,9(19):138-140. 345. 陈群梅,黄益军,陈汝文,林红霞,陈惠玲.中药足浴联合耳贴治疗脑动脉硬化症睡眠障碍的临床观察[J].中医临床研究,2017,9(01):25-27. 346. 徐燕,陈惠玲,黄益军,林红霞,陈汝文,钟志国.中药足浴结合辨证施膳对中风后失眠病人睡眠质量的影响[J].中西医结合心脑血管病杂志,2017,15(12):1504-1505. 347. 申斌,于川,刘福奇,徐寅平,邹忆怀.中药药氧疗法治疗缺血性脑卒中后失眠临床疗效观察[J].亚太传统医药,2017,13(13):95-97. 348. 姚新,孟晴,张红石.中药药膳配合耳穴压籽对脑卒中患者睡眠障碍的影响[J].长春中医药大学学报,2017,33(01):92-94.DOI:10.13463/j.cczyy.2017.01.032. 349. 谭玉婷.中药离子导入法在老年脑卒中后失眠病人中的应用效果观察[J].护理研究,2017,31(22):2737-2740. 350. 沈斌,姜文萍,刘龙彪,严晓铭,陈梅,沈艳,陈号.中药复方改善痰热扰心型中风后睡眠障碍临床疗效观察[J].中医药临床杂志,2017,29(10):1709-1711.DOI:10.16448/j.cjtcm.2017.0569. 351. 王建军,孙玫.中西医结合治疗缺血性脑卒中失眠症疗效观察[J].实用中医药杂志,2017,33(06):700-701. 352. 汪国爱.中西医结合治疗急性脑梗死伴失眠焦虑40例观察[J].浙江中医杂志,2017,52(07):534.DOI:10.13633/j.cnki.zjtcm.2017.07.045. 353. 崔丽娟,焦富英.正压通气联合银杏叶提取物对急性脑梗死合并阻塞性睡眠呼吸暂停综合征患者脑血流动力学及血液流变学的影响[J].现代中西医结合杂志,2017,26(32):3538-3541+3628. 354. 李敏,阿不都克尤木·阿不都热依木,曾科学.针药调神法治疗卒中后睡眠障碍52例临床观察[J].湖南中医杂志,2017,33(02):70-71.DOI:10.16808/j.cnki.issn1003-7705.2017.02.033. 355. 王楠.针灸治疗卒中后睡眠障碍的临床效果观察[J].内蒙古中医药,2017,36(Z1):197.DOI:10.16040/j.cnki.cn15-1101.2017.z1.155. 356. 吕丽.针刺治疗脑卒中并睡眠呼吸暂停综合征疗效观察[J].中国实用神经疾病杂志,2017,20(18):88-89. 357. 宋晶,王东岩,何雷,杨海永. 针刺四神聪增加觉醒脑电活动并改善卒中后阻塞性睡眠呼吸暂停患者日间嗜睡的临床观察[C]//2017世界针灸学术大会暨2017中国针灸学会年会论文集.[出版者不详],2017:537-539. 358. 任云锋,胡雨华.针刺十三鬼穴治疗中风后失眠的临床研究[J].陕西中医药大学学报,2017,40(03):32-34.DOI:10.13424/j.cnki.jsctcm.2017.03.013. 359. 蔺伟. 针刺十三鬼穴治疗中风后失眠的临床观察[D].安徽中医药大学,2017. 360. 吴佳苹,于海波.针刺任督二脉联合皮内针治疗缺血性脑梗死睡眠-觉醒节律紊乱随机平行对照研究[J].实用中医内科杂志,2017,31(10):76-78.DOI:10.13729/j.issn.1671-7813.2017.10.28. 361. 毛旺.针刺联合艾灸治疗脑中风后失眠疗效观察[J].内蒙古中医药,2017,36(20):105.DOI:10.16040/j.cnki.cn15-1101.2017.20.110. 362. 孙华,肖洒,黄庆丽.针刺结合加味黄连温胆汤治疗卒中后睡眠障碍临床观察[J].实用中医药杂志,2017,33(03):236-237. 363. 刘军兴.针刺对脑卒中后失眠患者觉醒状态调节作用临床研究[J].中医学报,2017,32(12):2544-2548.DOI:10.16368/j.issn.1674-8999.2017.12.661. 364. 孟彦.针刺“心俞三神”穴配合枣仁归脾二花汤治疗中风后失眠疗效分析[J].实用中医药杂志,2017,33(02):124-125. 365. 雷亚玲,刘飞向,杨海侠,韩祖成.引阳入阴配合气息导引法干预卒中后抑郁伴失眠病人的疗效观察[J].中西医结合心脑血管病杂志,2017,15(23):3053-3055. 366. 尹晟.益肾活血法治疗肾阴虚兼血瘀型缺血性脑卒中后失眠30例[J].中医研究,2017,30(09):28-30. 367. 郭宇宙,于子浇,刘红爱,李少华.养血清脑颗粒对脑卒中后睡眠障碍患者症状改善及血清5-HT、BDNF水平的影响[J].疑难病杂志,2017,16(05):457-460. 368. 李艳,刘敏琦.养肝益肾安神颗粒配合耳穴贴压治疗中风后失眠的临床研究[J].中医临床研究,2017,9(33):46-48. 369. 刘露阳,王鹏琴.眼针治疗卒中后失眠的随机对照研究[J].针刺研究,2017,42(01):67-71.DOI:10.13702/j.1000-0607.2017.01.012. 370. 陈晓东,孙俊.研究“调阴阳五脏”针法治疗卒中后睡眠障碍的临床疗效[J].中医临床研究,2017,9(08):89-90. 371. 李成英,朱海平,王国香.压灸百会穴对脑卒中后失眠患者护理干预的临床观察[J].临床护理杂志,2017,16(04):39-41. 372. 倪斐琳,陈改平,单霄雁.穴位拍打改善缺血性脑卒中后失眠的临床观察[J].浙江中医杂志,2017,52(06):412.DOI:10.13633/j.cnki.zjtcm.2017.06.012. 373. 杨金锁,杨雨果,李金岑.醒脑开窍针刺法结合灵龟八法治疗脑卒中后昼间嗜睡的临床分析[J].智慧健康,2017,3(05):166-167.DOI:10.19335/j.cnki.2096-1219.2017.05.74. 374. 黎东,黎帅.五志过极护理结合体外反搏改善缺血性中风后睡眠障碍的疗效对比观察[J].现代医药卫生,2017,33(16):2536-2538. 375. 蒋菁菁,张新玉.五行音乐联合耳穴压丸治疗脑卒中不寐患者的效果观察[J].当代护士(中旬刊),2017(10):87-88. 376. 谢宇锋,陈赟,王曙辉,吴云天,刘牧军.围剿推拿疗法对脑卒中后睡眠倒错患者多导睡眠图的影响[J].中医药导报,2017,23(09):69-72.DOI:10.13862/j.cnki.cn43-1446/r.2017.09.023. 377. 陈前,李丽.推拿配合经颅磁刺激治疗脑卒中后失眠临床研究[J].山东中医杂志,2017,36(05):387-389.DOI:10.16295/j.cnki.0257-358x.2017.05.012. 378. 龙凌. 头枕部推拿治疗脑梗死后轻度认知障碍合并睡眠障碍的临床观察[D].湖北中医药大学,2017. 379. 闵婕.头针联合高压氧治疗中风后失眠[J].世界最新医学信息文摘,2017,17(A3):228-229.DOI:10.19613/j.cnki.1671-3141.2017.103.161. 380. 王智琴.头部按摩加经穴梳理治疗脑卒中患者失眠的疗效观察[J].中国现代医生,2017,55(18):123-125. 381. 张菁华.通络益髓法对缺血性卒中后失眠的应用体会[J].内蒙古中医药,2017,36(01):29.DOI:10.16040/j.cnki.cn15-1101.2017.01.028. 382. 程英龙. 通督补肾针刺法治疗中风后失眠疗效观察[D].黑龙江中医药大学,2017. 383. 吴王芳,张永华.酸枣仁汤结合电子灸治疗脑梗死后失眠的临床疗效观察[J].黑龙江中医药,2017,46(04):45-47. 384. 吴辉丽.睡眠限制疗法结合通督调神针法在脑卒中睡眠障碍中的应用效果分析[J].亚太传统医药,2017,13(07):110-111. 385. 张亚云. 失眠颗粒治疗脑卒中后失眠的（肝火扰心型）的临床观察[D]. 陕西中医药大学,2017. DOI:10.7666/d.D01327326. 386. 吴小丽. 神阙雷火灸对脑卒中后失眠患者睡眠功能的影响[D].福建中医药大学,2017. 387. 贾盛豪,吴颖华,王生力.三黄安神汤治疗脑梗塞后痰热内扰型失眠临床疗效[J].亚太传统医药,2017,13(11):138-139. 388. 孙建光.祛瘀化痰开窍方治疗急性脑梗死合并阻塞性睡眠呼吸暂停低通气综合征临床观察[J].中国中医急症,2017,26(09):1633-1635. 389. 林悦佳,马朝晖,沈虹,江敏青.祛风化痰、活血通络法联合耳穴压豆治疗风痰上扰夹瘀型脑卒中后失眠症疗效观察[J].陕西中医,2017,38(07):852-853. 390. 颜光华.清热安神汤联合失眠三针治疗脑梗死后痰热内扰型失眠症98例临床观察[J].海峡药学,2017,29(09):166-167. 391. 崔青松.清热安神汤联合失眠三针治疗脑梗死后痰热内扰型失眠症[J].世界最新医学信息文摘,2017,17(A0):175.DOI:10.19613/j.cnki.1671-3141.2017.100.134. 392. 龚阳陵,万裕萍,龚墩.清宁片联合针刺十三鬼穴治疗中风后失眠疗效观察[J].陕西中医,2017,38(12):1630-1632. 393. 钱海彦.帕罗西汀联合百乐眠对脑卒中睡眠障碍患者的治疗效果及对睡眠质量的影响[J].中国继续医学教育,2017,9(11):198-199. 394. 蔡利颖,丁金环.帕罗西汀联合百乐眠对脑卒中睡眠障碍患者的治疗效果分析[J].中国卫生标准管理,2017,8(16):100-101. 395. 杨雪,林萍,郑英梅,郑亚兰.脑卒中睡眠障碍的中医护理干预探讨[J].中国当代医药,2017,24(27):154-156+159. 396. 倪斐琳,桑丽清,单霄雁. 脑卒中后失眠中医护理临床路径初探[J]. 浙江临床医学,2017(12). 397. 吴楚燕.脑卒中后失眠症的中医辨证施护[J].护理研究,2017,31(02):210-211. 398. 张小健,刘晶晶,王迎昌,焦雪蕾,周琦.脑心同治法对老年卒中后失眠患者总体睡眠状态及失眠药物剂量的影响[J].世界中医药,2017,12(02):269-271. 399. 李从刚. 加味丹栀逍遥散治疗缺血性中风病恢复期失眠的临床研究[D].云南中医学院,2017. 400. 张小健,刘晶晶,王迎昌,焦雪蕾.黄连阿胶汤辨证加减对阴虚火旺型老年卒中后失眠患者总体睡眠状态及失眠药物剂量的影响[J].空军医学杂志,2017,33(02):110-112+117. 401. 胡世荣,刘燕娜,王雪送,范媛媛.耳穴压豆联合穴位贴敷与两项技术独立应用对中风后失眠的效果[J].系统医学,2017,2(15):124-126.DOI:10.19368/j.cnki.2096-1782.2017.15.124. 402. 谢珊,吴华,徐秋霞,傅荣.耳穴贴压法对于脑卒中后抑郁伴失眠的临床效果与分析[J].国际精神病学杂志,2017,44(06):1057-1059.DOI:10.13479/j.cnki.jip.2017.06.029. 403. 刘娜,张子丽.耳穴揿针联合西药治疗脑卒中后失眠临床观察[J].浙江中医药大学学报,2017,41(11):907-910.DOI:10.16466/j.issn1005-5509.2017.11.016. 404. 刘素芳,张远玲,李霞,张慧慧,沈薇.耳穴埋豆配合放松训练对中风后失眠的护理疗效观察[J].安徽医药,2017,21(11):2132-2135. 405. 蔡春霞.耳穴埋豆联合酸枣仁汤加减治疗卒中后失眠的疗效及对血清TNF-α的影响[J].现代中西医结合杂志,2017,26(18):2033-2035. 406. 王杰. “额三针”为主治疗中风后失眠的临床研究[D].安徽中医药大学,2017. 407. 马增明. 调督安神针法治疗脑卒中后睡眠障碍的临床观察[D].黑龙江中医药大学,2017. 408. 皮巧红,于雪飞,刘宏伟.点穴疗法及中药足浴护理改善脑卒中后失眠的临床观察[J].湖南中医药大学学报,2017,37(04):406-408. 409. 游菲,唐雷,马朝阳,丁琳.低频电穴位刺激治疗脑卒中后失眠患者的疗效及对血浆多巴胺含量的影响[J].中华物理医学与康复杂志,2017,39(07):507-510. 410. 张美景.地黄饮子加减联合穴位贴敷法治疗中风后失眠的疗效观察[J].中西医结合心血管病电子杂志,2017,5(30):168-169.DOI:10.16282/j.cnki.cn11-9336/r.2017.30.136. 411. 李乔.黛力新联合舒肝解郁胶囊对脑卒中睡眠障碍患者的治疗效果及对睡眠质量的影响[J].中国疗养医学,2017,26(05):529-530.DOI:10.13517/j.cnki.ccm.2017.05.035. 412. 郭莎莎,徐锦平.从肝脾两脏论治卒中后睡眠障碍[J].亚太传统医药,2017,13(22):36-37. 413. 刘春甦.从肝论治应用中药联合奥氮平治疗脑卒中后睡眠障碍的研究[J].甘肃医药,2017,36(12):1029-1031.DOI:10.15975/j.cnki.gsyy.2017.12.012. 414. 郭昊睿. 柴芩温胆汤加减治疗缺血性卒中后失眠（肝郁痰热型）的临床研究[D].河南中医药大学,2017. 415. 李科学,刘敏杰.柴胡与龙骨牡蛎汤治疗缺血性中风后失眠的效果[J].临床医药文献电子杂志,2017,4(85):16767-16768.DOI:10.16281/j.cnki.jocml.2017.85.123. 416. 汤瑞珠.柴胡加龙骨牡蛎汤治疗中风后失眠随机平行对照研究[J].实用中医内科杂志,2017,31(04):17-20.DOI:10.13729/j.issn.1671-7813.2017.04.07. 417. 崔译心,王京军,阎晓悦.补心养肝方结合西药治疗脑梗塞后失眠的临床观察[J].内蒙古中医药,2017,36(17):39.DOI:10.16040/j.cnki.cn15-1101.2017.17.038. 418. 黄宇. 百乐眠胶囊治疗老年脑卒中后失眠的效果[J]. 神经疾病与精神卫生,2017(3). DOI:10.3969/j.issn.1009-6574.2017.03.015. 419. 王界成.百乐眠胶囊联合劳拉西泮治疗脑卒中睡眠障碍的临床观察[J].中西医结合心脑血管病杂志,2017,15(20):2626-2629. 420. 王冬梅. 百乐眠胶囊联合黛力新治疗脑梗死后焦虑抑郁失眠症状的疗效观察[J]. 中国急救医学,2017(z1). DOI:10.3969/j.issn.1002-1949.2017.z1.170. 421. 彭川.安神补脑液联合舍曲林治疗脑梗死后睡眠障碍的疗效观察[J].现代药物与临床,2017,32(03):407-410. 422. 曹亚芬,李丽娟,肖利亚.卒中后睡眠障碍患者中医延续护理的效果评价[J].护理学杂志,2018,33(18):90-93. 423. 何丹丹,钟雯,朱旗霞,陈雪莲.子午流注循经按摩改善脑卒中后失眠的疗效观察[J].中外医学研究,2018,16(24):164-165.DOI:10.14033/j.cnki.cfmr.2018.24.081. 424. 盖利利. “滋水涵木针刺法”治疗阴虚火旺型中风后失眠临床疗效观察[D].黑龙江中医药大学,2018. 425. 马新军.逐瘀安神方联合益肾调督养心针法治疗卒中后失眠的临床研究[J].中西医结合心脑血管病杂志,2018,16(20):2937-2940. 426. 张晓乐,狄灵,韩祖成,杨海侠,李小波.中医综合疗法治疗卒中后失眠35例临床观察[J].湖南中医杂志,2018,34(01):15-17.DOI:10.16808/j.cnki.issn1003-7705.2018.01.005. 427. 李岩,漆璐,冯兴中,王兴河.中医非药物治疗卒中后睡眠障碍疗效的系统性评价[J].北京中医药,2018,37(12):1172-1177.DOI:10.16025/j.1674-1307.2018.12.020. 428. 季静,蔡玉芬,董娟.中药药枕联合西药对老年脑梗死伴发焦虑失眠患者的效果分析[J].山西医药杂志,2018,47(19):2266-2268. 429. 黄清霞,钟应虎.中药药氧疗法治疗缺血性脑卒中后失眠临床观察[J].实用中医药杂志,2018,34(07):777-778. 430. 刘晶晶,张文娟. 中药熏洗联合耳穴压豆对中风后失眠患者的护理疗效观察[J]. 心理医生,2018(32). 431. 牛雁.中西医结合治疗脑卒中后失眠临床观察[J].光明中医,2018,33(18):2744-2745. 432. 栾丽平,赵立岩.中西医结合护理方案对缺血性脑梗死合并睡眠障碍患者的作用研究[J].世界睡眠医学杂志,2018,5(04):475-477. 433. 刘玉洁,周林芳,周凌云,赖华寿,刘晓冰,金远林.中风后失眠从“瘀”论治心得[J].江苏中医药,2018,50(10):22-24. 434. 谢晓娟.针灸治疗中风后睡眠障碍临床疗效及对神经功能的影响[J].现代中西医结合杂志,2018,27(29):3271-3273. 435. 刘驰,郑自龙,罗丽芳,周舰.针灸治疗脑卒中合并睡眠呼吸暂停综合征40例[J].环球中医药,2018,11(07):1095-1097. 436. 李福强.针灸联合中药治疗对高血压脑出血偏瘫合并睡眠障碍患者康复效果的影响[J].世界睡眠医学杂志,2018,5(01):75-79. 437. 马允浩,高旸.针刺治疗中风后失眠伴夜尿频疗效观察[J].实用中医药杂志,2018,34(01):109-111. 438. 宋裕如. 针刺五心穴治疗卒中后失眠的中医症状临床疗效观察[D]. 2018. 439. 李福强.针刺五腧穴对脑梗死后患者睡眠质量的影响[J].世界睡眠医学杂志,2018,5(10):1124-1126. 440. 宋秀娟,王银宁,李国徽,赵芳.针刺十三鬼穴治疗缺血性中风后气虚血瘀型不寐的临床研究[J].双足与保健,2018,27(02):182-183.DOI:10.19589/j.cnki.issn1004-6569.2018.02.182. 441. 王焱平,张钦昌,王振焕.针刺配合药物对脑梗死后失眠症患者血清5-HT、BDNF及Orexin-A水平的影响[J].上海针灸杂志,2018,37(11):1254-1258.DOI:10.13460/j.issn.1005-0957.2018.11.1254. 442. 宋剑英.针刺配合心理调护治疗中风后失眠临床观察[J].实用中医药杂志,2018,34(07):832-833. 443. 劳祥婷,彭柳莹,廉永红,卢栋明,廖薇,李羚,吴腊梅,杨进.针刺配合耳穴压籽治疗中风后难治性失眠[J].河南中医,2018,38(09):1367-1369.DOI:10.16367/j.issn.1003-5028.2018.09.0365. 444. 付梦雪,张先庚,陈雨萍,周海燕,刘林峰.针刺配合耳穴贴压治疗脑中风后失眠疗效的系统评价[J].中国疗养医学,2018,27(07):691-695.DOI:10.13517/j.cnki.ccm.2018.07.006. 445. 向君.针刺配合耳穴按压基础上联合康复治疗对于改善脑梗死后并发失眠的临床疗效探析[J].中国农村卫生,2018(23):64-65. 446. 陈婉珉,冯婉霞,潘红珊.针刺联合重复经颅磁刺激治疗脑卒中后睡眠障碍临床观察[J].光明中医,2018,33(03):400-402. 447. 朱金妹,何俊,焦素芹,韩乐园.针刺联合康复治疗对脑卒中后阻塞性睡眠呼吸暂停并吞咽障碍临床观察[J].上海针灸杂志,2018,37(12):1368-1372.DOI:10.13460/j.issn.1005-0957.2018.12.1368. 448. 廖群好.针刺联合艾灸百会穴治疗中风后失眠40例[J].中医外治杂志,2018,27(03):39-40. 449. 邢晓彤,张智龙.张智龙运用养血柔肝针法治疗卒中后睡眠障碍经验[J].湖南中医杂志,2018,34(07):30-32.DOI:10.16808/j.cnki.issn1003-7705.2018.07.011. 450. 陈子. 张亚平汇阳聚气针法治疗脑病学术观点及临床经验探究[D].华北理工大学,2018. 451. 梁丹丹,方琼,刘照勇.运动结合刺血治疗卒中后睡眠障碍的临床研究[J].泰山医学院学报,2018,39(07):753-755. 452. 张清奇,常耀辉.养血清脑颗粒治疗对脑卒中后睡眠障碍患者的影响[J].四川解剖学杂志,2018,26(02):74-75+78. 453. 朱作权,桂心,杨叔英,刘露.养血清脑颗粒联合右佐匹隆片治疗急性脑卒中后失眠疗效观察[J].中国药业,2018,27(11):69-72. 454. 何敏,刘志强,刘艳龙,朱晓钢.养血清脑颗粒联合右佐匹克隆片治疗卒中后睡眠障碍的疗效[J].实用临床医学,2018,19(04):10-13+22.DOI:10.13764/j.cnki.lcsy.2018.04.004. 455. 刘丹,李健,陈薇,程亮,刘树利,户文娟,刘文芳.养血清脑颗粒联合米氮平治疗脑卒中后抑郁伴失眠的临床观察[J].湖南中医药大学学报,2018,38(05):582-585. 456. 郭莎莎. 眼针疗法治疗中风后失眠的随机对照研究[D].辽宁中医药大学,2018. 457. 胡楠,于睿.眼针联合保神汤治疗中风后不寐临床观察[J].辽宁中医药大学学报,2018,20(02):108-110.DOI:10.13194/j.issn.1673-842x.2018.02.031. 458. 刘燕妮,张婷,黄国燊,闫咏梅.闫咏梅教授运用醒脑解郁胶囊“异病同治”临床经验探析[J].陕西中医药大学学报,2018,41(04):26-29.DOI:10.13424/j.cnki.jsctcm.2018.04.009. 459. 周锐钧,周光辉.血府逐瘀汤加减联合针灸治疗中风后不寐患者的临床研究[J].光明中医,2018,33(16):2367-2369. 460. 郑萍红,李洁,郝宗霞,等. 血府安神汤治疗中风后遗症期痰瘀互结型不寐的临床疗效观察[J]. 中国全科医学,2018(z1). DOI:10.3969/j.issn.1007-9572.2018.z1.203. 461. 杨华军.穴位贴敷结合经颅磁刺激改善卒中后睡眠障碍的临床观察[J].世界睡眠医学杂志,2018,5(08):896-899. 462. 王锁,潘洪萍,程红亮,朱才丰.穴位按摩配合中药外敷对脑卒中后睡眠障碍护理效果观察[J].中医药临床杂志,2018,30(05):955-958.DOI:10.16448/j.cjtcm.2018.0291. 463. 罗玲.穴位按摩联合护理干预对中风恢复期心脾两虚型失眠患者生活质量的影响[J].当代护士(下旬刊),2018,25(01):105-107. 464. 王磊,徐寅平.逍遥散加减方联合揿针治疗肝郁脾虚型卒中后失眠的疗效观察[J].环球中医药,2018,11(04):596-599. 465. 董莉莉,马丽虹,王建玲,马帅.五行音乐结合重复低频经颅磁刺激治疗中风后失眠的临床疗效观察[J].中西医结合心脑血管病杂志,2018,16(11):1494-1496. 466. 杨坤,蔡圣朝,费爱华,秦晓凤,吴萌萌,贺成功,王丽,代飞.温阳补肾灸治疗脑卒中后失眠疗效观察[J].现代中西医结合杂志,2018,27(09):954-956. 467. 杨秀斌,李文杰.温胆汤加味联合耳穴贴压治疗脑梗塞后痰热内扰型失眠症疗效观察[J].海南医学,2018,29(05):697-699. 468. 刘夏.通络安神方足浴治疗中风后失眠患者的临床观察[J].中国民间疗法,2018,26(08):22-23.DOI:10.19621/j.cnki.11-3555/r.2018.0813. 469. 郎显兵,谭曦.通督调神针法对脑卒中后睡眠障碍患者睡眠质量和神经功能的影响[J].河北中医药学报,2018,33(03):46-49.DOI:10.16370/j.cnki.13-1214/r.2018.03.014. 470. 黄香龙,栾运慧,李林.天王补心丹联合经颅磁刺激在脑卒中后睡眠障碍治疗观察[J].内蒙古中医药,2018,37(07):22-23.DOI:10.16040/j.cnki.cn15-1101.2018.07.014. 471. 孙巧杰.酸枣仁汤加减治疗中风后失眠的临床研究[J].中医临床研究,2018,10(06):57-58. 472. 黄伟,滕海英,毛媛媛.疏肝解郁胶囊联合艾司唑仑治疗卒中后睡眠障碍的疗效观察[J].中西医结合心脑血管病杂志,2018,16(08):1105-1107. 473. 杨洋,汪志云,郭洁,姚扬,拱忠影.舒脑欣滴丸治疗脑卒中合并睡眠障碍的临床疗效观察[J].中西医结合心脑血管病杂志,2018,16(11):1608-1610. 474. 宋秀娟,王银宁,李国徽,等. 缺血性中风后气虚血瘀型不寐的十三鬼穴应用研究[J]. 大家健康（下旬版）,2018(2). DOI:10.3969/j.issn.1009-6019(x).2018.02.038. 475. 姜玉辉.清热安神汤联合失眠三针治疗脑梗死后痰热内扰型失眠症临床观察[J].中国处方药,2018,16(12):114-115. 476. 王磊,徐寅平.揿针配合中药为主治疗肝郁脾虚型卒中后失眠疗效观察[J].上海针灸杂志,2018,37(01):6-10.DOI:10.13460/j.issn.1005-0957.2018.01.0006. 477. 黄栌仪,贺军.浅析“通任顺气”针刺法的临床应用[J].中华针灸电子杂志,2018,7(01):34-36. 478. 武凌锋.浅谈卒中后睡眠障碍中医治疗的临床效果观察[J].世界睡眠医学杂志,2018,5(05):567-568. 479. 熊良淦.帕罗西汀联合百乐眠对脑卒中睡眠障碍患者睡眠质量的影响[J].基层医学论坛,2018,22(23):3236-3237.DOI:10.19435/j.1672-1721.2018.23.024. 480. 赵国勇,张琼.帕罗西汀联合百乐眠对脑卒中睡眠障碍患者的治疗研究[J].内蒙古医学杂志,2018,50(09):1098-1099.DOI:10.16096/J.cnki.nmgyxzz.2018.50.09.045. 481. 陈延玲. 宁心安神汤联合针刺治疗中风后心肾不交型不寐的临床研究[D]. 2018. 482. 王彩荣,曹静.宁神补心丸、氟西汀联合心理康复治疗青年脑卒中伴抑郁及睡眠障碍临床研究[J].现代中西医结合杂志,2018,27(24):2705-2707. 483. 徐立萍.脑卒中睡眠障碍中西医结合护理效果观察[J].世界睡眠医学杂志,2018,5(02):229-232. 484. 杨芳.脑卒中后睡眠障碍等常见长期症状中西医特色护理技术干预有效性探讨[J].中西医结合心血管病电子杂志,2018,6(23):12-14.DOI:10.16282/j.cnki.cn11-9336/r.2018.23.006. 485. 吉婷婷. 脑梗死后逮症患者失眠的中医护理及施行效果探究[J]. 医药前沿,2018(29). DOI:10.3969/j.issn.2095-1752.2018.29.228. 486. 刘丹,李健,陈薇,程亮,刘树利,卢文娟.米氮平联合养血清脑颗粒治疗脑卒中抑郁失眠临床研究[J].中国药业,2018,27(23):53-55. 487. 姚财文.落花生枝叶制剂治疗中风后失眠的临床疗效观察[J].内蒙古中医药,2018,37(01):24-25.DOI:10.16040/j.cnki.cn15-1101.2018.01.019. 488. 任正意,李红亮,齐运卫.灵龟八法穴位敷贴治疗缺血中风后丑寅时不寐疗效观察[J].福建中医药,2018,49(05):76-78.DOI:10.13260/j.cnki.jfjtcm.011713. 489. 侯志涛,孙忠人,孙申田.经颅重复针刺激疗法对卒中后失眠患者血清食欲素A水平的影响[J].中国针灸,2018,38(10):1039-1042+1052.DOI:10.13703/j.0255-2930.2018.10.003. 490. 张东升.加味菖蒲郁金汤治疗脑卒中后睡眠障碍痰瘀阻窍证临床研究[J].亚太传统医药,2018,14(06):198-200. 491. 陈香帆. 加味补阳还五汤治疗老年中风后失眠气虚血瘀证临床疗效的观察[D].湖南中医药大学,2018. 492. 周红霞,王彦华,刘向哲,王伟民,张燕平,路永坤,杨国防.黄连阿胶汤加减治疗脑卒中后失眠阴虚火旺证的疗效观察[J].中国实验方剂学杂志,2018,24(10):187-192.DOI:10.13422/j.cnki.syfjx.20181028. 493. 史春林,陈建权,张海生,张国良,刘大永,刘冬敏,王玉超.化浊毒中药对2型糖尿病并发急性脑梗死失眠患者的影响[J].现代养生,2018(16):149-150. 494. 朱金妹,周沁,王瑾,焦素芹.化痰清瘀汤联合口咽部康复治疗缺血性脑卒中后阻塞性睡眠呼吸暂停并吞咽障碍(痰瘀阻窍)随机平行对照研究[J].实用中医内科杂志,2018,32(07):4-8.DOI:10.13729/j.issn.1671-7813.z20180076. 495. 刘家峰,杨帆.复式补泻针刺法结合乌梅丸治疗脑卒中后睡眠障碍临床研究[J].国际中医中药杂志,2018,40(09):805-809. 496. 孙建光,郑彩莲,孙爱红,艾媛媛,陈媛媛,王亚静,李坤,郑向荣.复健通脉饮对急性脑梗死合并阻塞性睡眠呼吸暂停低通气综合征病人远期疗效及复发率的影响[J].中西医结合心脑血管病杂志,2018,16(23):3415-3418. 497. 王瑶瑶,陈紫君,王燕飞,张震中.芳香药枕治疗脑梗死后肝郁化火型失眠症34例[J].浙江中医杂志,2018,53(02):107.DOI:10.13633/j.cnki.zjtcm.2018.02.017. 498. 李文莉,戴娜.芳香药枕联合头部穴位按摩治疗中风后失眠的护理效果观察[J].光明中医,2018,33(22):3418-3420. 499. 沈芝琴,姚强.耳穴压丸结合足底按摩/穴位贴敷治疗卒中后睡眠障碍的临床价值[J].航空航天医学杂志,2018,29(04):435-436. 500. 沈燕菊,徐佳,张颖顺. 耳穴埋籽联合辩证施护在改善中风患者失眠中的应用[J]. 健康必读,2018(25). 501. 方向华.耳穴埋籽联合百乐眠治疗中风后失眠的效果[J].实用临床医药杂志,2018,22(23):111-112. 502. 王鑫.耳穴埋豆联合中药湿敷对中风后失眠的效果观察[J].天津护理,2018,26(03):341-343. 503. 马振宇.耳穴埋豆联合中药改善脑卒中后抑郁患者失眠的疗效[J].中国现代医生,2018,56(14):82-85. 504. 沈芝琴,汤春红,姚强,李慧,赵海音.耳穴埋豆联合酸枣仁汤加减治疗卒中后失眠的疗效及对血清TNF-α的影响[J].中国医药导刊,2018,20(04):212-215. 505. 李春颖,高俊虹.多种针法联合治疗卒中后睡眠障碍的临床疗效观察[J].中国中医基础医学杂志,2018,24(05):656-657+687. 506. 何俊,黄亮,朱金妹,钱雪,言丽香,焦素芹,张丽霞,王彤.定穴熏蒸联合下颏抗阻力训练对脑卒中后阻塞性睡眠呼吸暂停患者口咽部形态及功能的影响研究[J].实用心脑肺血管病杂志,2018,26(05):52-56. 507. 郝瑞民,韩舰华.调神温经养血针法治疗中风后不安腿综合征验案1则[J].湖南中医杂志,2018,34(09):107-108.DOI:10.16808/j.cnki.issn1003-7705.2018.09.049. 508. 黄亮,何俊,章金蓉,丁渝权,言丽香,王彤,张丽霞.电针联合口肌生物反馈系统训练治疗脑卒中后重度阻塞性睡眠呼吸暂停的临床疗效[J].实用心脑肺血管病杂志,2018,26(05):56-59. 509. 王彦华,路永坤,张燕平,刘向哲,周红霞,杨国防,姜秀云.涤痰汤加减治疗中青年急性缺血性脑卒中合并OSAHS痰瘀互结证的疗效观察[J].中国实验方剂学杂志,2018,24(15):204-209.DOI:10.13422/j.cnki.syfjx.20180926. 510. 徐秀梅,曾学波.从脾胃论治中风后失眠[J].时珍国医国药,2018,29(02):393-394. 511. 刘静,强锋,洪登攀.从肝论治脑梗塞后失眠体会[J].临床医学研究与实践,2018,3(04):94-95.DOI:10.19347/j.cnki.2096-1413.201804046. 512. 肖文,边娜,杨丽英,蒲晓龙.百乐眠胶囊联合双重抗血小板治疗进展性脑梗死伴睡眠障碍患者的临床效果及其作用机制分析[J].四川医学,2018,39(12):1400-1404.DOI:10.16252/j.cnki.issn1004-0501-2018.12.022. 513. 李粉霞,刘丽萍.百乐眠胶囊联合护理干预治疗脑梗死并睡眠障碍102例临床观察[J].中国药物与临床,2018,18(10):1866-1867. 514. 王鑫栋. 艾灸调控卒中后睡眠障碍患者5-HT、NA、Ach等脑内神经递质的研究[D].安徽中医药大学,2018. 515. 万慧玲.足疗联合心理疗法治疗脑卒中后抑郁性失眠的临床分析[J].双足与保健,2019,28(08):7-8.DOI:10.19589/j.cnki.issn1004-6569.2019.08.007. 516. 王国丰.足底穴位按摩治疗脑卒中后睡眠障碍的临床疗效观察[J].双足与保健,2019,28(20):45-46.DOI:10.19589/j.cnki.issn1004-6569.2019.20.045. 517. 曹江,张岩,沈文婧.自拟涤痰汤辅助阿普唑仑治疗中青年急性脑梗死伴OSAHS疗效及对炎性细胞因子、氧化应激指标的影响[J].中国中医急症,2019,28(02):343-345. 518. 张璐.中医综合护理干预对脑卒中患者睡眠障碍的影响[J].中西医结合心血管病电子杂志,2019,7(31):155-156.DOI:10.16282/j.cnki.cn11-9336/r.2019.31.122. 519. 刘喜会.中医药干预对改善脑卒中后睡眠障碍疗效与安全性的系统评价探讨[J].世界睡眠医学杂志,2019,6(06):740-741. 520. 刘围.中医健康管理平台对脑卒中睡眠障碍患者预后的影响[J].西部中医药,2019,32(08):130-132. 521. 李小娟,李颖,曹高凡,张霞.中医护理技术联合CBT干预对中风后失眠患者睡眠质量及心理健康影响[J].中西医结合心血管病电子杂志,2019,7(01):11-13.DOI:10.16282/j.cnki.cn11-9336/r.2019.01.008. 522. 田信,田琼.中医护理干预对脑血管意外患者睡眠障碍的影响[J].世界最新医学信息文摘,2019,19(36):275+284.DOI:10.19613/j.cnki.1671-3141.2019.36.192. 523. 曹玲玲,徐明红,马先军,王惠.中药药枕联合rTMS治疗脑卒中失眠的效果观察[J].中西医结合护理(中英文),2019,5(05):55-57. 524. 梁玲,王文在.中西医结合治疗脑卒中合并睡眠呼吸暂停综合征临床效果[J].世界睡眠医学杂志,2019,6(04):499-500. 525. 左国平.中西医结合治疗急性脑梗死伴失眠焦虑20例观察[J].世界最新医学信息文摘,2019,19(41):180+184.DOI:10.19613/j.cnki.1671-3141.2019.41.113. 526. 蔡丹,刘存勇,李军.中西医结合治疗方案对缺血性脑梗死合并失眠患者的疗效[J].世界睡眠医学杂志,2019,6(08):1032-1034. 527. 张雁,张凤俠,杨孝菊,耿晓静,孙艳军,赵亮,付慧霄,代丽红.中西医结合治疗对脑梗死后睡眠障碍患者情绪状态与睡眠质量的影响研究[J].河北医学,2019,25(04):655-658. 528. 朱小燕.针灸治疗中风后睡眠障碍患者的临床疗效及对患者神经功能的影响[J].中医临床研究,2019,11(33):104-105+118. 529. 周彩虹.针灸配合心理调护治疗脑卒中失眠的临床观察[J].中国医药指南,2019,17(18):181-182.DOI:10.15912/j.cnki.gocm.2019.18.147. 530. 田苗利. 针灸腹部穴位配合脑康液对脑卒中后气虚血瘀型患者失眠的疗效观察[J]. 健康必读,2019(29). 531. 孙千贺. 针刺头部督脉和足太阳经穴治疗卒中后认知障碍伴失眠症的临床观察[D].黑龙江中医药大学,2019.DOI:10.27127/d.cnki.ghlzu.2019.000427. 532. 杨强,王东,王瑞辉.针刺十三鬼穴结合补阳还五汤治疗中风后气虚血瘀型失眠临床研究[J].针灸临床杂志,2019,35(08):13-15. 533. 李俭.针刺配合耳穴压籽治疗中风后失眠的临床疗效评价[J].中国医药指南,2019,17(03):167-168.DOI:10.15912/j.cnki.gocm.2019.03.139. 534. 刘牧军,王建莉,叶美霞.针刺联合心理干预对中风后睡眠障碍患者睡眠质量的影响[J].实用临床护理学电子杂志,2019,4(04):89+91. 535. 游毅,陈卉,李土明,谢安劼,王长德.针刺联合低频疗法治疗缺血性脑卒中后失眠[J].吉林中医药,2019,39(11):1476-1480.DOI:10.13463/j.cnki.jlzyy.2019.11.022. 536. 张东,焦富英,甘雨,乔敏.针刺联合安神脐贴对脑梗死后伴有失眠患者促眠作用及血浆5-HT、NE、DA影响[J].辽宁中医药大学学报,2019,21(03):82-85.DOI:10.13194/j.issn.1673-842x.2019.03.023. 537. 杨云涛,刘明,吴毅明,左刚.针刺结合刘茂林经验方治疗脑卒中后失眠症临床研究[J].新中医,2019,51(09):238-240.DOI:10.13457/j.cnki.jncm.2019.09.071. 538. 范玉英.针刺结合耳穴治疗脑梗死后失眠的症状转归分析[J].实用中医内科杂志,2019,33(11):83-85.DOI:10.13729/j.issn.1671-7813.z20190402. 539. 黄伟柱,冼土生,罗志东,庞家亮.右佐匹克隆联合甜梦口服液治疗轻度脑卒中患者睡眠障碍的临床疗效[J].齐齐哈尔医学院学报,2019,40(12):1471-1472. 540. 王双双,朱青霞.益气调血安神汤联合针刺治疗中风后失眠的临床观察[J].中医临床研究,2019,11(36):100-102. 541. 柴冰燕,颜克松,高福临,吴瑞鹏,张毅.养血清脑颗粒治疗卒中后睡眠障碍的Meta分析[J].世界最新医学信息文摘,2019,19(86):13-16+21.DOI:10.19613/j.cnki.1671-3141.2019.86.006. 542. 王璞,周佩洋,张贵斌.养血清脑颗粒联合艾司唑仑治疗脑卒中后睡眠障碍疗效及对血清5-HT、BDNF水平的影响[J].现代中西医结合杂志,2019,28(03):318-321. 543. 刘国卿.养肝益肾安神颗粒配合耳穴贴压治疗中风后失眠的临床研究[J].中医临床研究,2019,11(33):27-28. 544. 郭燕芹. 穴位贴敷疗法对中风后失眠患者的护理疗效观察[D].河北医科大学,2019. 545. 罗昕.穴位埋针治疗中风后失眠的临床疗效观察[D].辽宁中医药大学,2019.DOI:10.27213/d.cnki.glnzc.2019.000246. 546. 罗昕,王恩龙.穴位埋针法治疗中风后失眠30例的临床观察[J].名医,2019(03):117. 547. 李景莉.穴位按摩联合五行音乐疗法对脑卒中后抑郁伴睡眠障碍患者临床症状和生活质量的影响[J].四川中医,2019,37(05):190-192. 548. 刘晨阳,路明.醒脑开窍针法结合百会穴灸法治疗中风后失眠验案1则[J].湖南中医杂志,2019,35(10):85-86.DOI:10.16808/j.cnki.issn1003-7705.2019.10.034. 549. 万福铭,王文熠.醒脑开窍针刺法配合灵龟八法治疗脑卒中后昼间嗜睡的随机对照及随访研究[J].辽宁中医杂志,2019,46(07):1501-1504.DOI:10.13192/j.issn.1000-1719.2019.07.045. 550. 郭秋芳.醒脑开窍针刺法结合养血柔肝针法治疗中风后失眠的临床研究[D]. 2019. 551. 张瑜. “醒脑调腑”针刺法治疗中风后失眠的临床疗效评价研究[D].宁夏医科大学,2019. 552. 呼延静,田申,雷正权,郭成莲.小脑顶核电刺激结合针刺治疗卒中后失眠疗效观察[J].现代中医药,2019,39(02):23-26.DOI:10.13424/j.cnki.mtcm.2019.02.007. 553. 黄臻颖,邓芳平.小剂量多塞平联合五行音乐疗法在脑卒中后睡眠障碍患者中的应用效果[J].长治医学院学报,2019,33(01):65-67+76. 554. 李琳青.五行音乐联合耳穴压豆治疗脑卒中后失眠患者的效果分析[J].中国实用医药,2019,14(16):149-150.DOI:10.14163/j.cnki.11-5547/r.2019.16.081. 555. 申淑侠.乌灵胶囊联合曲唑酮治疗脑梗塞伴发睡眠障碍的临床效果[J].临床医学研究与实践,2019,4(26):41-42.DOI:10.19347/j.cnki.2096-1413.201926018. 556. 姜明静.文拉法辛联合疏肝解郁胶囊治疗缺血性脑卒中后睡眠障碍的疗效观察[J].世界睡眠医学杂志,2019,6(07):879-880. 557. 刘牧军,刘中迪,李振南,王建莉.温灸脐疗法对中风恢复期患者睡眠倒错的护理效果[J].临床医药文献电子杂志,2019,6(07):104.DOI:10.16281/j.cnki.jocml.2019.07.094. 558. 许文杰,蒋瑞冲,周一心,秦勇.王翘楚教授经验方治疗肝郁瘀阻型脑卒中后失眠临床研究[J].陕西中医,2019,40(07):867-870. 559. 钱雪峰.推拿联合电针治疗卒中后睡眠障碍[J].世界睡眠医学杂志,2019,6(05):580-582. 560. 胡孔翠,胡谢.酸枣仁汤加减方治疗脑卒中后失眠的效果观察[J].当代医药论丛,2019,17(08):200-201. 561. 俞帼英,张雯静.疏肝安神汤联合针刺、穴位按摩治疗脑梗死后痰热内扰型失眠症的效果观察[J].中国初级卫生保健,2019,33(07):87-88. 562. 刘顺美,卢宁,刘丽,付相利,朱晓燕.舒肝解郁胶囊联合中药浴足辅助治疗脑卒中患者睡眠障碍效果观察[J].长治医学院学报,2019,33(03):213-216. 563. 赵甫刚,邢军,王亚辉,张立庄,邸晓敏,常丽静,王静.舌针配合百笑灸治疗脑卒中后瘀血内阻型失眠40例临床观察[J].中国误诊学杂志,2019,14(01):15-17. 564. 李庆兵,罗才贵.三部益智调神推拿法治疗脑卒中后睡眠倒错疗效观察[J].北京中医药,2019,38(02):143-145.DOI:10.16025/j.1674-1307.2019.02.014. 565. 赖华寿. 祛瘀生新针法联合大艾条灸治疗中风后不寐的临床疗效观察[D].广州中医药大学,2019.DOI:10.27044/d.cnki.ggzzu.2019.000879. 566. 姚旭洁. 揿针埋针对中风后失眠干预效果的临床研究[D]. 2019. 567. 陈韻,陈鑫金,徐月萍.宁神补心丸对脑卒中伴抑郁及睡眠障碍患者神经功能 血清C反应蛋白及神经营养因子-3水平的影响[J].中国药物与临床,2019,19(08):1241-1244. 568. 宋智慧,连玲霞.内外治结合治疗中风后失眠临床观察[J].世界最新医学信息文摘,2019,19(25):141.DOI:10.19613/j.cnki.1671-3141.2019.25.103. 569. 张相锋.脑卒中后睡眠障碍应用中医针灸疗法的效果观察及评价[J].中西医结合心血管病电子杂志,2019,7(27):171.DOI:10.16282/j.cnki.cn11-9336/r.2019.27.135. 570. 赵媛,王耀辉,王翠,袁捷,陈杰,韩祖成.脑卒中后睡眠障碍研究进展及韩祖成临证经验总结[J].辽宁中医药大学学报,2019,21(12):119-122.DOI:10.13194/j.issn.1673-842x.2019.12.030. 571. 吕小笑,周文军,李美川.脑循环功能障碍治疗仪穴位治疗联合安眠汤对中风后失眠的改善作用[J].中国中医药科技,2019,26(02):273-275. 572. 梁箭,王储蓄,陈幸生.芒针透刺督脉治疗脑卒中后睡眠倒错的临床疗效观察[J].湖北中医杂志,2018,40(12):34-36. 573. 韦振英,严群.经颅磁低频电治疗联合音乐疗法改善急性脑梗塞肝阳上亢型不寐症的效果观察[J].世界最新医学信息文摘,2019,19(28):174.DOI:10.19613/j.cnki.1671-3141.2019.28.082. 574. 盛威,吕凌,杨雪,等. 经颅磁刺激结合耳压治疗中风后睡眠障碍的疗效观察[J]. 医学食疗与健康,2019(3). 575. 黄霞,粟胜勇,陈舒,黄小珍.健脾调神法针灸治疗中风后失眠的临床疗效观察[J].辽宁中医杂志,2019,46(09):1943-1946.DOI:10.13192/j.issn.1000-1719.2019.09.046. 576. 汪雨欣. 加味交泰丸治疗心肾不交型中风后失眠的临床研究[D].湖北中医药大学,2019. 577. 刘晶晶,焦雪蕾,张小建,刘宏伟,周艳明.活血化瘀解郁方联合通督益脑安神针法治疗卒中后失眠气虚血瘀证的疗效观察[J].世界中医药,2019,14(06):1548-1551. 578. 刘红娟,王慧萍,徐丽平.当归六黄汤治疗失眠的临床运用[J].世界最新医学信息文摘,2019,19(34):154+156.DOI:10.19613/j.cnki.1671-3141.2019.34.112. 579. 王献.黄连温胆汤联合酸枣仁汤对卒中后睡眠障碍患者的疗效及睡眠质量的影响[J].中国冶金工业医学杂志,2019,36(02):206-207.DOI:10.13586/j.cnki.yjyx1984.2019.02.072. 580. 朱金妹,何俊,朱海颖,王欢,季盼盼.黄连温胆汤辅以脑电仿生电刺激仪治疗卒中后痰热扰心型睡眠障碍疗效观察[J].现代中西医结合杂志,2019,28(15):1606-1610. 581. 张瑞,王敏,陈红霞.海马益智散对缺血性脑卒中伴阻塞性睡眠呼吸暂停综合征患者认知功能与睡眠结构的影响[J].新中医,2019,51(11):99-102.DOI:10.13457/j.cnki.jncm.2019.11.029. 582. 罗彩容,张朝霞,赵斌斌.桂枝加龙骨牡蛎汤联合“靳三针”治疗中风后睡眠障碍的临床疗效[J].内蒙古中医药,2019,38(10):131-132.DOI:10.16040/j.cnki.cn15-1101.2019.10.081. 583. 王天龙. 刮痧联合中药治疗缺血性中风后失眠（肝郁化火证）的临床研究[D].长春中医药大学,2019.DOI:10.26980/d.cnki.gcczc.2019.000309. 584. 宋福云.耳穴贴压治疗卒中后睡眠障碍的疗效[J].心电图杂志(电子版),2019,8(01):46-47. 585. 李霞,李壮苗,林文颖,李荣清,王诗莹.耳穴贴磁对卒中后睡眠障碍患者的疗效观察[J].贵阳中医学院学报,2019,41(06):49-54.DOI:10.16588/j.cnki.issn1002-1108.2019.06.011. 586. 鲍颂杨,黄莹.耳穴埋豆配合放松训练对中风后失眠的护理疗效评价[J].中西医结合心血管病电子杂志,2019,7(17):84.DOI:10.16282/j.cnki.cn11-9336/r.2019.17.068. 587. 赵洋.耳穴埋豆联合中药治疗缺血性中风后失眠（肝郁化火证）的临床疗效观察[D].长春中医药大学,2019.DOI:10.26980/d.cnki.gcczc.2019.000400. 588. 汤海燕,温春娣,刘向力,温馨,陈晓娜,陈希姬.耳穴埋豆干预中风后失眠的护理体会[J].按摩与康复医学,2019,10(08):73-74.DOI:10.19787/j.issn.1008-1879.2019.08.035. 589. 张秀敏. 耳穴埋豆+酸枣仁汤加减治疗卒中后失眠的临床疗效观察及应用价值分析[J]. 健康必读,2019(5). 590. 张霜梅,吉晶,胡丽竹,刘红宇,邱朝阳,祝维峰.耳穴疗法治疗卒中后失眠的随机对照试验Meta分析[J].上海中医药大学学报,2019,33(03):10-18+23.DOI:10.16306/j.1008-861x.2019.03.003. 591. 江玲玲,邓翠鸣,李雅青,李秀兰.耳穴联合坎离砂外敷治疗脑梗死患者睡眠障碍的疗效观察[J].中医临床研究,2019,11(05):87-89. 592. 盛威,杨雪,吕凌,王晓明,段佰龙.多导睡眠图观察经颅磁刺激联合音乐电针治疗脑卒中后睡眠障碍的临床研究[J].针灸临床杂志,2019,35(04):7-12. 593. 杨明玉.对接受耳穴埋籽治疗的脑梗死后失眠患者进行综合护理的效果研讨[J].当代医药论丛,2019,17(23):235-236. 594. 李珊珊,曹燕,徐世芬. 电针治疗卒中后失眠的随机对照试验[C]//新时代 新思维 新跨越 新发展——2019中国针灸学会年会暨40周年回顾论文集.[出版者不详],2019:433-437.DOI:10.26914/c.cnkihy.2019.003947. 595. 王东岩,宋晶,何雷,袁小涵,李颖. 电针四神聪改善卒中相关失眠患者认知功能的临床观察[C]//新时代 新思维 新跨越 新发展——2019中国针灸学会年会暨40周年回顾论文集.[出版者不详],2019:596-602.DOI:10.26914/c.cnkihy.2019.003986. 596. 李颖. 电针四神聪改善脑梗死相关失眠患者认知功能的临床观察[D].黑龙江中医药大学,2019.DOI:10.27127/d.cnki.ghlzu.2019.000179. 597. 伦智勇,高尚.点穴疗法及中药足浴护理改善脑卒中后失眠的临床观察[J].双足与保健,2019,28(20):1-2+5.DOI:10.19589/j.cnki.issn1004-6569.2019.20.001. 598. 张霜梅,吉晶,邱朝阳,祝维峰,李衍滨.单纯针灸对比西药治疗卒中后失眠随机对照试验的Meta分析[J].中华中医药学刊,2019,37(12):2843-2852.DOI:10.13193/j.issn.1673-7717.2019.12.007. 599. 翟艳红.丹参川芎嗪辅助治疗缺血性脑卒中伴睡眠障碍的效果观察[J].世界睡眠医学杂志,2019,6(07):885-886. 600. 王岳青.从清热化痰法论治急性脑梗死后睡眠障碍的临床研究[D].北京中医药大学,2019. 601. 武润梅,李佩芳,郝丽霞,许敏,陈改花.陈改花运用自拟调和肝脾方治疗脑卒中后不寐[J].中国民间疗法,2019,27(14):8-9.DOI:10.19621/j.cnki.11-3555/r.2019.1404. 602. 霍间甜,张婉容,杨慧贤,危娟.常规护理配合耳穴压豆联合低频电刺激法在中风后失眠病人中的应用[J].全科护理,2019,17(19):2367-2369. 603. 邱文君. 菖志黄连温胆汤治疗缺血性脑卒中后失眠痰热内扰证的临床研究[D].山东中医药大学,2019.DOI:10.27282/d.cnki.gsdzu.2019.000706. 604. 付晓波.柴胡加龙骨牡蛎汤治疗脑卒中后失眠患者的临床疗效[J].医疗装备,2019,32(20):115-116. 605. 魏雪琴.参芪五味子胶囊联合阿普唑仑治疗脑卒中后合并失眠多梦的临床研究[J].世界睡眠医学杂志,2019,6(02):155-156. 606. 秦海军,张倩,陈艺钦,周爽.补肾通督针刺法对中风后失眠患者睡眠质量及生活质量的影响[J].现代中西医结合杂志,2019,28(30):3378-3381. 607. 陈朋.“标本配穴”温针灸联合头背部推拿治疗脑梗死后失眠的疗效观察[J].中国社区医师,2019,35(12):94-95. 608. 罗美凤.辨证护理结合耳穴贴压治疗中风恢复期心脾两虚型失眠的效果评价[J].世界睡眠医学杂志,2019,6(01):90-91. 609. 黄海霞,李可臣.辨证护理结合耳穴埋豆治疗中风恢复期心脾两虚型失眠临床观察[J].实用临床护理学电子杂志,2019,4(50):46+57. 610. 刘卫林,杨耀峰,马利利.百乐眠胶囊联合舍曲林治疗脑卒中睡眠障碍的效果观察[J].贵州医药,2019,43(10):1600-1602. 611. 杜婧. 百乐眠胶囊联合黛力新治疗脑梗死后焦虑抑郁失眠症状的效果分析[J]. 神州,2019(4). DOI:10.3969/j.issn.1009-5071.2019.04.202. 612. 徐树平,孙福. 百乐眠胶囊联合黛力新缓解脑梗死后焦虑抑郁 失眠症状的治疗效果分析[J]. 心理医生,2019(6). 613. 张景涵,周海芳,吴静.艾盐穴位热熨对脑卒中后睡眠障碍的作用观察[J].中国中医药科技,2019,26(06):915-917. 614. 于文琦,吴少霞,刘娟,朱乐英,潘锐焕,许钦玲.艾灸神门、足三里穴改善卒中后睡眠障碍的临床研究[J].中国继续医学教育,2019,11(15):146-148. 615. 刘伟清,詹德利.养血清脑颗粒联合艾司唑仑治疗脑卒中后睡眠障碍疗效及对患者血清5-羟色胺水平的影响[J].中国处方药,2020,18(05):46-47. 616. 刘艳,裴胜利,杜谢琴,姚瑶,刘英,高芳芳.基于脑卒中后睡眠障碍患者中医体质的个体化护理[J].护理学杂志,2020,35(22):48-51. 617. 刘岳.针灸联合中药对高血压脑出血偏瘫合并睡眠障碍患者康复效果的影响[J].世界睡眠医学杂志,2020,7(05):807-809. 618. 罗媛媛,杨森.眼针结合体针治疗卒中后失眠的临床观察[J].辽宁中医杂志,2020,47(05):171-173.DOI:10.13192/j.issn.1000-1719.2020.05.051. 619. 马菲,张建宾,马永利,王晓宇,李华南.血府逐瘀汤加减配合喉三针对缺血性卒中合并睡眠呼吸暂停低通气综合征患者呼吸紊乱及血氧调节的影响[J].辽宁中医杂志,2020,47(09):79-82.DOI:10.13192/j.issn.1000-1719.2020.09.026. 620. 蒙兰英,李靖杰. 艾灸护理干预对卒中后睡眠障碍患者影响的研究[J]. 世界最新医学信息文摘（连续型电子期刊）,2020(58). DOI:10.3969/j.issn.1671-3141.2020.58.117. 621. 苗国印.灵胶囊合并生物电刺激治疗脑卒中后睡眠障碍[J].罕少疾病杂志,2020,27(01):35-36+65. 622. 宁建平. 中药内服联合rTMS在脑卒中失眠治疗中的效果观察[J]. 特别健康,2020(28). 623. 彭莉,甘琴,吴英,潘礼寿,肖蓉,丁丹.血府逐瘀汤治疗缺血性脑卒中患者睡眠障碍的临床疗效[J].实用临床医药杂志,2020,24(21):46-49. 624. 权东海. 柴胡加龙骨牡蛎汤治疗中风后失眠的效果评价[J]. 东方药膳,2020(22). 625. 石少红,石蕾.经颅超声联合头皮针在急性缺血性脑卒中后睡眠障碍患者中的应用[J].医疗装备,2020,33(20):55-56. 626. 眭淑彦,张震中,朱明锦,金鑫.调任通督针联合活血解郁汤辅助治疗对脑卒中后认知障碍并发失眠患者的影响[J].现代实用医学,2020,32(11):1357-1358. 627. 眭淑彦,朱明锦,张震中.加味酸枣仁汤治疗卒中后认知障碍伴失眠对患者HPA轴功能、睡眠质量及认知功能的影响[J].中国现代医生,2020,58(30):14-17+21. 628. 孙芳玉. 针灸联合药物治疗对缺血性脑卒中偏瘫合并睡眠障碍患者的康复效果观察[J]. 反射疗法与康复医学,2020(21). 629. 孙瑶,李柏霖,胡楠.耳穴揿针联合体针对脑卒中后失眠患者血清细胞因子及神经递质表达的影响[J].辽宁中医药大学学报,2020,22(12):160-163.DOI:10.13194/j.issn.1673-842x.2020.12.036. 630. 唐琳芳,陈荣群,梁喜春. 中药特色香囊治疗缺血性中风后失眠症的临床效果观察[J]. 临床医药文献电子杂志,2020(77). 631. 屠娜飞,倪斐琳,桑丽清. 耳穴揿针治疗脑卒中后抑郁失眠的疗效观察[J]. 浙江临床医学,2020(4). 632. 汪敏,谢勤,孙芸,王兴荣,陆伟兰,熊漫.中医羽调音乐在脑卒中后睡眠障碍患者中的应用[J].国际护理学杂志,2020,39(16):2970-2972. 633. 王彩娇,丁芳,金瑛.耳穴压豆联合“引阳入阴”按摩导引法对缺血性卒中后睡眠障碍的临床疗效观察[J].中国现代医生,2020,58(08):82-84. 634. 王枫. 耳穴压豆疗法对中风后失眠患者的护理疗效观察[J]. 东方药膳,2020(8). 635. 王海涛,王颖,吴海洋.针刺联合艾灸涌泉穴治疗中风后失眠的临床观察[J].中医药临床杂志,2020,32(06):1106-1109.DOI:10.16448/j.cjtcm.2020.0630. 636. 王倩.针灸治疗中风后睡眠障碍临床疗效及对神经功能的影响评价[J]. 医药前沿,2020(32). 637. 王文芳,马卓君.中药穴位贴敷联合耳穴贴压法对脑卒中后失眠的治疗效果观察[J].中国乡村医药,2020,27(24):30-31.DOI:10.19542/j.cnki.1006-5180.004674. 638. 王晓聪,刘江,夏洪涛,翟永政,吴喜华.黄连温胆汤联合艾司唑仑片治疗卒中后睡眠障碍痰热内扰证临床研究[J].国际中医中药杂志,2020(04):319-320-321-322-323. 639. 王旭峰. 穴位按摩配合中药外敷涌泉穴在中风后失眠患者中的应用[J]. 首都食品与医药,2020(18). DOI:10.3969/j.issn.1005-8257.2020.18.132. 640. 王雅娟,段红莉,周华,等. 归脾汤加穴位敷贴治疗缺血性中风后失眠的有效性分析[J]. 自我保健,2020(20). 641. 魏善卫,苗文霞.情志护理对脑梗死急性期患者焦虑和睡眠障碍的影响[J].世界睡眠医学杂志,2020,7(09):1641-1642. 642. 吴迪,曾群. 中医针灸治疗中风患者失眠症的疗效观察[J]. 健康必读,2020(30). 643. 吴裕成. 常规针刺联合揿针对中风后失眠患者的疗效观察[J]. 世界最新医学信息文摘（连续型电子期刊, 2020(96). DOI:10.3969/j.issn.1671-3141.2020.96.089. 644. 吴政,金晓晓,仲劼怡,等. 补肾助阳针刺法治疗中风后失眠的疗效及对患者血清SP、NPY水平的影响[J]. 河南中医,2020(7). DOI:10.16367/j.issn.1003-5028.2020.07.0278. 645. 许宏霞. 清热安神汤联合失眠三针治疗脑梗死后痰热内扰型失眠症的临床研究[J]. 医学食疗与健康,2020(7). 646. 许军. 枣仁归脾二花汤联合耳穴埋豆对卒中后失眠患者血清5-HT、DA水平及睡眠质量的影响[J]. 临床医学研究与实践,2020(21). DOI:10.19347/j.cnki.2096-1413.202021046. 647. 闫雪. 针刺配合刮痧疗法治疗脑卒中后失眠的临床研究[J]. 世界睡眠医学杂志,2020(1). DOI:10.3969/j.issn.2095-7130.2020.01.015. 648. [1]杨光,岳扬,才蕊.银杏叶提取物注射液联合持续正压通气治疗急性脑梗死合并OSAHS 43例[J].西部中医药,2020,33(04):99-102. 649. [1]杨玲,郭耀光,林炜钰,严小勤.针刺结合耳穴压籽治疗中风后失眠的临床探究[J].中国全科医学,2020,23(S2):213-216. 650. 姚青,徐颖梅,钱立锋. 耳穴揿针联合五音疗法改善脑卒中后失眠效果观察[J]. 中国乡村医药,2020(24). DOI:10.3969/j.issn.1006-5180.2020.24.018. 651. 冶尕西,张瑜,关淑婷,等. “醒脑调腑”针刺法治疗中风后失眠的临床疗效评价[J]. 辽宁中医杂志,2020(10). DOI:10.13192/j.issn.1000-1719.2020.10.043. 652. 叶稳田,易珍,胡广芹,等. 鍉圆针定量痧疗术对改善脑卒中后睡眠障碍的临床观察[J]. 当代医学,2020(16). DOI:10.3969/j.issn.1009-4393.2020.16.005. 653. 尤峰.育阴宁神汤对老年脑卒中后睡眠障碍患者睡眠质量及康复效果的影响[J].中医临床研究,2020,12(16):87-91. 654. 余李强,刘芳,沈翠玲,方素珠,刘秦宇,王俊蕊.艾灸治疗脑卒中后睡眠障碍效果的系统评价[J].贵州中医药大学学报,2020,42(01):72-77.DOI:10.16588/j.cnki.issn1002-1108.2020.01.018. 655. 张光友,沈世英.益心醒脑汤联合针刺治疗脑卒中后抑郁伴失眠临床观察[J].四川中医,2020,38(12):149-151. 656. 张佳翔. 原络配穴接经疗法治疗中风后心肾不交型失眠的临床观察[D]. 2020. 657. 张久梅,李小兰. 耳穴压豆配合贴敷涌泉穴治疗脑卒中失眠患者的疗效评价[J]. 中国实用医药,2020(28). DOI:10.14163/j.cnki.11-5547/r.2020.28.071. 658. 张丽,胡军,薛炘,等. 改良易筋经对脑卒中恢复期睡眠障碍患者睡眠质量的影响[J]. 康复学报,2020(1). DOI:10.3724/SP.J.1329.2020.01015. 659. 张明秋. 穴位埋针法治疗中风后失眠的效果研究[J]. 反射疗法与康复医学,2020(17). 660. 张琼帅,孙绍骞,汲广成,等. 针刺治疗中风后失眠有效性及安全性的Meta分析[J]. 时珍国医国药,2020(5). DOI:10.3969/j.issn.1008-0805.2020.05.080. 661. 张霜梅. 卒中后失眠的临床研究及其中医药治疗的Meta分析[D]. 2020. 662. 张伟, 马坤琴, 肖洪波, et al. 针刺加重复经颅磁刺激治疗脑卒中后失眠的临床观察[J]. 针灸推拿医学（英文版）,2020(2). DOI:https://doi.org/10.1007/s11726-020-1166-z. 663. 张向宇,高旸,武连仲,等. 针刺五心穴治疗卒中后心肾不交型失眠临床观察[J]. 中华中医药杂志,2020(9). 664. 张严严,王非,顾秀锋. 中医针灸治疗中风患者失眠症的疗效观察[J]. 中西医结合心血管病电子杂志,2020(15). 665. 张阳普,夏文广,郑婵娟,等. 通任调督针法联合r-TMS治疗脑卒中后失眠的临床疗效评价[J]. 时珍国医国药,2020(11). DOI:10.3969/j.issn.1008-0805.2020.11.044. 666. 张园园,陈美琦,郑泽,等. 原穴温针灸法治疗脑卒中后失眠的临床研究[J]. 中国中医药现代远程教育,2020(7). DOI:10.3969/j.issn.1672-2779.2020.07.036. 667. 张志琴,王燕鸽,武金程,等. 百乐眠胶囊联合双抗治疗进展性脑梗死伴睡眠障碍的效果及对血清神经递质水平的影响[J]. 国际精神病学杂志,2020(1). 668. 章涛,徐珲,丁晔. 乌灵胶囊联合谷维素片治疗急性轻度脑梗死伴失眠效果观察[J]. 中国乡村医药,2020(23). DOI:10.3969/j.issn.1006-5180.2020.23.009. 669. 赵湾湾,张亚楠. 艾司唑仑片联合疏肝安神颗粒、耳穴压豆在肝气郁结型缺血性脑卒中后失眠患者中的应用[J]. 河南医学研究,2020(29). DOI:10.3969/j.issn.1004-437X.2020.29.047. 670. 赵欣,姬孟艳,董强. 加味丹栀逍遥散对中风后失眠患者睡眠质量及生活质量影响[J]. 检验医学与临床,2020(11). DOI:10.3969/j.issn.1672-9455.2020.11.018. 671. 郑建峰.曲唑酮联合乌灵胶囊治疗脑梗死伴发睡眠障碍的临床疗效及安全性[J].临床合理用药杂志,2020,13(28):39-40.DOI:10.15887/j.cnki.13-1389/r.2020.28.016. 672. 郑杰,林振. 头针治疗对中风后睡眠障碍患者焦虑抑郁症状及睡眠质量的影响[J]. 健康研究,2020(1). DOI:10.3969/j.issn.1674-6449.2020.01.030. 673. 钟宾谟,谌凯,何惠萍. 浮针联合耳穴压豆治疗卒中后失眠临床观察[J]. 特别健康,2020(10). 674. 周城林,刘琼,梅琰,陈毅,龙再慧.归脾汤加味联合小剂量曲唑酮治疗脑卒中后失眠及对血脂及血流动力学的影响[J].中医临床研究,2020,12(21):53-56. 675. 朱锦如. 以“补下清上”为法分型论治卒中后（后遗症期）失眠[D]. 2020. 676. 邹愉龙,宋玉凡,林高城,等. 浅针治疗脑卒中后失眠的临床疗效观察[J]. 中外医学研究,2020(24). DOI:10.14033/j.cnki.cfmr.2020.24.062. 677. Dai N, Li Y, Sun J, Li F, Xiong H. Self-Designed Ningxin Anshen Formula for Treatment of Post-ischemic Stroke Insomnia: A Randomized Controlled Trial. Front Neurol. 2020 Nov 9;11:537402. doi: 10.3389/fneur.2020.537402. PMID: 33240190; PMCID: PMC7680871. 678. Dong X, Zhang R, Guo Y, Chen L, Liu Y. The efficacy of Qigong exercises for post-stroke mental disorders and sleep disorders: Protocol for a systematic review and meta-analysis. Medicine (Baltimore). 2020 Aug 21;99(34):e21784. doi: 10.1097/MD.0000000000021784. PMID: 32846809; PMCID: PMC7447379. 679. Xiang J, Li H, Xiong J, Hua F, Huang S, Jiang Y, Zhou X, Liao K, Xu L. Acupuncture for post-stroke insomnia: A protocol for systematic review and meta-analysis. Medicine (Baltimore). 2020 Jul 24;99(30):e21381. doi: 10.1097/MD.0000000000021381. PMID: 32791749; PMCID: PMC7386967. 680. Zhang Y, He X, Hu S, Hu S, He F, Shen Y, Zhao F, Zhang Q, Liu T, Wang C. Efficacy and safety of massage in the treatment of post-stroke insomnia: A protocol for systematic review and meta-analysis. Medicine (Baltimore). 2020 Dec 18;99(51):e23598. doi: 10.1097/MD.0000000000023598. PMID: 33371092; PMCID: PMC7748325. 681. 孙俊俊,左政,宋冉,包雄英,朱勉生. 针刺联合艾灸治疗脑卒中后失眠疗效和安全性的系统评价和meta分析方案[C]//“中医针灸”申遗十周年特别活动暨世界针灸学会联合会2020国际针灸学术研讨会论文集.[出版者不详],2020:129-130.DOI:10.26914/c.cnkihy.2020.027430. 682. 敖祖松. 温阳汤加味联合艾司唑仑对脑梗塞合并失眠患者神经功能及睡眠质量的影响[J]. 辽宁医学杂志,2020(3). 683. 蔡淑满. 通督调神针刺法对台湾地区缺血性脑卒中后失眠的临床效果观察[D].广州中医药大学,2020.DOI:10.27044/d.cnki.ggzzu.2020.000018. 684. 曹燕,吴君怡,蔡丽,等. 针刺治疗老年缺血性卒中后失眠的临床疗效观察[J]. 老年医学与保健,2020(3). DOI:10.3969/j.issn.1008-8296.2020.03.035. 685. 常咪娜,王艳艳,张虹. 五音特色护理干预在脑卒中睡眠障碍患者中的应用效果[J]. 临床医学研究与实践,2020(15). DOI:10.19347/j.cnki.2096-1413.202015055. 686. 陈芳. 火龙罐综合灸改善中风后失眠的研究[J]. 健康必读,2020(18). 687. 陈锦泳,何玉琴,林小锋,等. 吴茱萸穴位贴敷联合耳穴压豆对卒中后失眠患者睡眠质量及血BDNF和NIHSS的影响[J]. 现代医院,2020(10). DOI:10.3969/j.issn.1671-332X.2020.10.040. 688. 陈蒙利,李佳,张瑾,等. 耳穴埋豆联合五行音乐疗法在脑卒中睡眠障碍患者中的应用[J]. 山西医药杂志,2020(21). DOI:10.3969/j.issn.0253-9926.2020.21.056. 689. 陈天容,毕建平. 针刺廉泉对脑卒中合并轻度睡眠呼吸暂停低通气综合征远期疗效的观察[J]. 医药界,2020(2). 690. 丁懿. “五音调神”法治疗脑卒中后失眠的临床研究[D]. 2020. 691. 董芸. 用加味菖蒲郁金汤对痰瘀阻窍型脑卒中后睡眠障碍患者进行治疗的效果研究[J]. 当代医药论丛,2020(9). DOI:10.3969/j.issn.2095-7629.2020.09.148. 692. 樊谷,王涛. 王涛针灸治疗中风后不寐的临床经验总结[J]. 世界最新医学信息文摘（连续型电子期刊）,2020(A2). DOI:10.3969/j.issn.1671-3141.2020.102.171. 693. 付燕琼. 柴胡加龙骨牡蛎汤治疗脑卒中后失眠的临床观察[J]. 中国民间疗法,2020(15). DOI:10.19621/j.cnki.11-3555/r.2020.1520. 694. 苟娟平,白小军,郑卫锋,呼睿,符奇飞.芒针透刺督脉组穴配合隔姜灸治疗脑卒中后睡眠障碍疗效及机制研究[J].山东中医药大学学报,2020,44(02):151-155.DOI:10.16294/j.cnki.1007-659x.2020.02.008. 695. 郭建赟,赵苏鑫,赵洪昊,等. 陈改花从“肝”论治卒中后抑郁伴失眠经验[J]. 中医药临床杂志,2020(10). DOI:10.16448/j.cjtcm.2020.1018. 696. 郭耀光,孙光伟,胡纪可,等. 体针联合耳穴贴压对脑卒中后失眠的影响[J]. 四川中医,2020(7). 697. 韩玉爱,兰亚平,谢潇侠.谢潇侠主任医师从肝论治中风后睡眠障碍经验[J].中医临床研究,2020,12(13):95-99. 698. 何凤麟,徐莉娅,程平荣,刘嘉佳.平调阴阳针刺法治疗脑卒中后失眠的临床观察[J].实用心脑肺血管病杂志,2020,28(S2):244-246. 699. 何素玲,方晓燕,阮兢.复式补泻针刺法结合靳三针治疗卒中后睡眠障碍的疗效及对PSQI和NIHSS评分影响[J].针灸临床杂志,2020,36(12):35-39. 700. 黄学娣,蒋司晨,樊萍,胡小玲,严毅,程富香,唐莉华.柔肝安神汤治疗脑卒中后睡眠障碍临床观察[J].光明中医,2020,35(17):2619-2621. 701. 贾艳花. 穴位按摩联合中药外敷涌泉穴对中风后失眠患者PSQI评分的影响[J]. 首都食品与医药,2020(18). DOI:10.3969/j.issn.1005-8257.2020.18.130. 702. 赖永金,马淑娟,魏威. 热敏灸联合佐匹克隆对卒中后睡眠障碍患者睡眠质量的影响[J]. 医学理论与实践,2020(11). DOI:10.19381/j.issn.1001-7585.2020.11.022.\ 703. 乐智卿,陶平,于萍,等. 头皮针治疗卒中后睡眠障碍的临床观察[J]. 江西医药,2020(4). DOI:10.3969/j.issn.1006-2238.2020.04.020. 704. 李光辉,张晓乐,田立军,等. 回神颗粒治疗急性脑梗死后不宁腿综合征的效果[J]. 中国城乡企业卫生,2020(12). DOI:10.16286/j.1003-5052.2020.12.012. 705. 李金山,樊跃辉. 探讨针灸对高血压脑出血偏瘫合并睡眠障碍患者康复效果的影响[J]. 世界最新医学信息文摘（连续型电子期刊）,2020(88). DOI:10.3969/j.issn.1671-3141.2020.88.105. 706. 李玲玲,王小燕,张雪雷. 时辰穴位贴敷用于老年卒中后失眠护理中的临床研究[J]. 临床医药文献电子杂志,2020(71). 707. 李梦雪,张之毓,王倩. 通督调神针法治疗脑卒中后失眠的疗效及其对神经递质水平的影响[J]. 世界中医药,2020(1). DOI:10.3969/j.issn.1673-7202.2020.01.022. 708. 李相君,金兰花.龙骨牡蛎汤联合针灸治疗缺血性脑卒中后抑郁性失眠的有效性分析[J].中医临床研究,2020,12(27):34-35. 709. 李延萍.中医综合护理在中风后心脾两虚型不寐中的应用[J].光明中医,2020,35(23):3804-3806. 710. 廖丽芬,廖燕清. 个性化护理辅助针灸在老年中风合并失眠患者中的应用效果观察[J]. 世界睡眠医学杂志,2020(9). DOI:10.3969/j.issn.2095-7130.2020.09.020. 711. 刘慧.中医辨证施护对脑卒中后失眠症患者睡眠质量及不良情绪的影响[J]. 国际护理学杂志,2020(23). DOI:10.3760/cma.j.cn221370-20190816-01336. 712. 刘晶晶,张小健,刘宏伟,等. 三七合枕清眠安汤联合通督益脑安神针治疗卒中后失眠气滞血瘀证疗效观察[J]. 现代中西医结合杂志,2020(18). DOI:10.3969/j.issn.1008-8849.2020.18.006. 713. 刘莲,李梦雪. 温针灸联合耳穴压丸治疗缺血性脑卒中心脾两虚型失眠的疗效观察[J]. 世界睡眠医学杂志,2020(4). DOI:10.3969/j.issn.2095-7130.2020.04.018. 714. 刘牧军,李振南,陈丹. 五志过极护理在体外反搏治疗缺血性脑卒中后睡眠障碍患者中的应用效果[J]. 中国当代医药,2020(21). DOI:10.3969/j.issn.1674-4721.2020.21.059. 715. 刘顺美,刘莉,付相利,等. 百乐眠胶囊辅助阿司匹林与氯吡格雷治疗脑梗死后睡眠障碍临床效果以及对睡眠质量、负性情绪的影响[J]. 中华中医药学刊,2020(9). DOI:10.13193/j.issn.1673-7717.2020.09.025. 716. 王建玲.中医针灸法治疗中风后睡眠障碍的临床研究[J].中医临床研究,2020,12(02):90-91. 717. 张俊杰,张丽华,刘悦. 中西医结合治疗中风后肝气郁结型失眠30例临床观察[J]. 湖南中医杂志,2020(3). DOI:10.16808/j.cnki.issn1003-7705.2020.03.015. 718. 聂芬芬,胡莎,周丽莎,等. 针刺治疗中风后失眠临床疗效Meta分析[J]. 陕西中医,2020(1). DOI:10.3969/j.issn.1000-7369.2020.01.035. 719. 闫雪. 针刺配合刮痧疗法治疗脑卒中后失眠的临床研究[J]. 世界睡眠医学杂志,2020(1). DOI:10.3969/j.issn.2095-7130.2020.01.015. 720. Wei Zhang et al. Clinical observation of acupuncture plus repetitive transcranial magnetic stimulation in the treatment of post-stroke insomnia[J]. Journal of Acupuncture and Tuina Science: Acupuncture, Moxibustion and Therapeutic Massage, 2020, 18(7) : 122-128. 721. 陈颖,马欣,毛水先,等. 穴位贴敷治疗中风恢复期合并失眠患者随机对照试验[J]. 中国中医基础医学杂志,2020(1). DOI:10.3969/j.issn.1006-3250.2020.01.029. 722. 何锦玉. 穴位按摩联合护理干预对中风恢复期心脾两虚型失眠患者生活质量的影响[J]. 实用临床护理学电子杂志,2020(10). 723. 李梦雪,张之毓,王倩. 通督调神针法治疗脑卒中后失眠的疗效及其对神经递质水平的影响[J]. 世界中医药,2020(1). DOI:10.3969/j.issn.1673-7202.2020.01.022. 724. 罗丽红,李昌生,赖秀娟. 四神针配合手智针治疗脑卒中后失眠临床观察[J]. 实用中医药杂志,2020(4). 725. 王雪霞,张洪雷. “三神针”治疗中风后失眠[J]. 中医学报,2020(3). DOI:10.16368/j.issn.1674-8999.2020.03.150. 726. 史春梅. 祛风化痰活血通络法联合耳穴压豆治疗脑卒中后失眠临床观察[J]. 光明中医,2020(1). DOI:10.3969/j.issn.1003-8914.2020.01.035. 727. 窦海玲,李世泽,赵松耀. 曲唑酮联合百乐眠治疗卒中后失眠的效果[J]. 河南医学研究,2020(7). DOI:10.3969/j.issn.1004-437X.2020.07.007. 728. 章颖,薛翠丽. 脑卒中相关睡眠障碍给予针刺治疗的效果与安全性评价[J]. 中国处方药,2020(2). DOI:10.3969/j.issn.1671-945X.2020.02.072. 729. 王艳. 脑卒中后睡眠障碍的中西医护理效果观察[J]. 中国城乡企业卫生,2020(1). DOI:10.16286/j.1003-5052.2020.01.046. 730. [1]杨晓静,赵辉,邓力威.脑卒中后康复期患者抑郁失眠的中医辨证施护[J].按摩与康复医学,2020,11(04):75-77.DOI:10.19787/j.issn.1008-1879.2020.04.030. 731. [1]王子豪,王恩龙.耳穴压豆治疗中风后失眠临床观察[J].湖北中医杂志,2020,42(01):33-34. 732. 姚青,钱立锋,陆海娟. 耳穴揿针联合引阳入阴推拿治疗脑卒中后失眠效果观察[J]. 中国乡村医药,2020(3). DOI:10.3969/j.issn.1006-5180.2020.03.009. 733. 曹燕,严寅杰,刘依萍,等. 调督安神针刺治疗卒中后失眠的随机对照研究[J]. 上海针灸杂志,2020(3). DOI:10.13460/j.issn.1005-0957.2020.03.0280. 734. 王改凤,王松龄. 导痰汤加减联合常规治疗对中风后OSAHS风痰瘀阻证患者的临床疗效[J]. 中成药,2020(3). DOI:10.3969/j.issn.1001-1528.2020.03.016. 735. 王双双,朱青霞.从瘀论治中风后失眠[J].中医临床研究,2020,12(03):60-61. 736. 莫柏威.柴胡加龙骨牡蛎汤治疗中风后失眠的效果观察[J].内蒙古中医药,2020,39(03):59-60.DOI:10.16040/j.cnki.cn15-1101.2020.03.036. 737. 张天虹,苏银彪,姚海军,何卫平.百乐眠胶囊联合黛力新治疗脑梗死后焦虑抑郁失眠症的效果观察[J].临床合理用药杂志,2020,13(10):10-11.DOI:10.15887/j.cnki.13-1389/r.2020.10.005. 738. 李贺. 中风后失眠采用健脾调神法针灸治疗的临床效果分析[J]. 养生保健指南,2021(20). 739. 李梦华,徐晔. 酸枣仁汤加减治疗脑卒中后失眠48例[J]. 河南中医,2021(6). DOI:10.16367/j.issn.1003-5028.2021.06.0193. 740. 李鹏,范振崴,张敏. 艾司唑仑联合八段锦治疗脑卒中患者失眠、焦虑及抑郁的效果研究[J]. 中华养生保健,2021(4). 741. 梁文萍. 中西医结合治疗脑梗死失眠疗效观察[J]. 实用中医药杂志,2021(4). 742. 刘吉权,邓丽霞. "眠三针"配合重复经颅磁刺激改善脑卒中后失眠的临床研究[J]. 中医药导报,2021(1). 743. 刘婧,刘睿,庄兰英. 醒脑开窍针法结合项八针治疗卒中后睡眠倒错的临床评价[J]. 江西中医药大学学报,2021(3). 744. 刘娟侠. 观察耳穴埋豆配合放松训练对中风后失眠的临床护理效果影响[J]. 东方药膳,2021(5). 745. 刘立斌,张雷,吴妍,等. 乌灵胶囊联合右佐匹克隆治疗脑卒中后失眠疗效观察[J]. 中国药业,2021(5). DOI:10.3969/j.issn.1006-4931.2021.05.020. 746. 潘丹,徐文斌,泮金亮,等. 醒脑开窍针法结合项八针治疗卒中后睡眠倒错临床观察[J]. 中国现代医生,2021(21). 747. 潘燕蝶,王凤玲,韩秀琴. 中药竹罐治疗中风风痰阻络证致睡眠障碍临床研究[J]. 四川中医,2021(7). 748. 彭美瑶,朱诗林. "中医辨体调护"改善气郁质中风恢复期失眠患者睡眠质量60例观察[J]. 湖南中医杂志,2021(7). DOI:10.16808/j.cnki.issn1003-7705.2021.07.036. 749. 阮娟娟,魏霞. 艾司唑仑联合自拟黄连清热化痰方对痰热内扰型卒中后失眠患者生活质量的影响[J]. 承德医学院学报,2021(4). 750. 盛珊珊. 优质护理联合中药药枕改善卒中后患者睡眠障碍的效果观察[J]. 东方药膳,2021(3). 751. 盛扬,魏海棠,张俊雄,等. 经颅磁电刺激联合头皮针治疗脑卒中后失眠患者的临床疗效[J]. 广西医学,2021(9). DOI:10.11675/j.issn.0253-4304.2021.09.26. 752. 石代乐,高继英,杨李鹏,等. 养血解郁醒脑汤联合曲舍林治疗脑出血睡眠障碍及对神经递质、炎症因子的影响[J]. 中华中医药学刊,2021(4). DOI:10.13193/j.issn.1673-7717.2021.04.055. 753. 舒适,王琦,陈宝瑾,等. 中药联合耳穴治疗肝郁脾虚型卒中后失眠临床疗效观察[J]. 中医药通报,2021(3). 754. 宋淑玲,周相娟,郑立强. 耳穴贴压联合针刺对中风后睡眠障碍患者睡眠质量和神经递质的影响[J]. 现代中西医结合杂志,2021(3). DOI:10.3969/j.issn.1008-8849.2021.03.014. 755. 王丹,钟建国. 基于"多感官刺激"理论的中医综合康复方案对老年缺血性脑卒中后失眠患者睡眠功能的影响[J]. 实用医学杂志,2021(12). DOI:10.3969/j.issn.1006⁃5725.2021.12.021. 756. 王东岩,霍宏,冯丽媛,等. 外金津、玉液不同靶向电针对脑梗死相关阻塞性睡眠呼吸暂停综合征患者颏舌肌收缩的影响[J]. 针灸临床杂志,2021(5). DOI:10.19917/j.cnki.1005-0779.021097. 757. 王凤玲,王宝爱,苟荣,等. 中药竹罐疗法对中风后睡眠质量及神经功能缺损的影响[J]. 中国医药导报,2021(11). 758. 王凤玲,王宝爱,黄惠芬,等. 中药竹罐疗法治疗中风后失眠[J]. 河南中医,2021(1). DOI:10.16367/j.issn.1003-5028.2021.01.0021. 759. Kim SH, Lim JH. Herbal medicine for post-stroke insomnia: A protocol for systematic review and meta-analysis. Medicine (Baltimore). 2021 Jun 4;100(22):e26223. doi: 10.1097/MD.0000000000026223. PMID: 34087901; PMCID: PMC8183778. 760. 王恒,杨本德. 隔药灸神阙穴联合中医情志疗法治疗卒中后抑郁失眠共病的临床研究[J]. 光明中医,2021(15). DOI:10.3969/j.issn.1003-8914.2021.15.038. 761. 王惠娟,曹惠茹. 分析耳穴埋豆干预对中风后失眠的护理改善效果[J]. 母婴世界,2021(13). 762. Sun J, Zuo Z, Song R, Bao X, Zhu M. Acupuncture combined with moxibustion for insomnia after stroke: A protocol for systematic review and meta analysis. Medicine (Baltimore). 2021 Jan 22;100(3):e24112. doi: 10.1097/MD.0000000000024112. PMID: 33546019; PMCID: PMC7837922. 763. 王玲姝,张宇,刘爽,等. 针刺联合低频重复经颅磁刺激治疗中风后失眠的随机对照研究[J]. 现代中西医结合杂志,2021(6). DOI:10.3969/j.issn.1008-8849.2021.06.009. 764. Zhan C, Hu ZD, Zhao Y, Fang XM, Cheng W, Lu S, Chen ZW. Acupuncture and related therapies for poststroke insomnia: A protocol for systematic review and network meta-analysis. Medicine (Baltimore). 2021 Mar 5;100(9):e25039. doi: 10.1097/MD.0000000000025039. PMID: 33655980; PMCID: PMC7939198. 765. 王晓娟,刘少妮,徐慧,等. 失眠认知行为疗法联合百乐眠胶囊对脑卒中后睡眠障碍患者睡眠质量和血清神经递质水平的影响[J]. 临床医学研究与实践,2021(18). DOI:10.19347/j.cnki.2096-1413.202118043. 766. 吴林,伍媛,劳祎林,等. 基于玄府理论探讨脑卒中后失眠的病机及治疗[J]. 辽宁中医杂志,2021(7). DOI:10.13192/j.issn.1000-1719.2021.07.018. 767. 吴晓. 耳穴埋豆干预中风后失眠的护理分析[J]. 糖尿病天地,2021(3). 768. 徐东娥,陈紫君,金金,等. 精油芳香疗法联合引阳入阴推拿干预脑卒中后不寐的效果观察[J]. 护理与康复,2021(5). DOI:10.3969/j.issn.1671-9875.2021.05.018. 769. 徐栋梁. 对脑卒中后睡眠障碍病人实施中医针灸疗法效果评价[J]. 中国保健营养,2021(17). 770. 徐丽娟,李海燕,田军彪. 益智解郁汤对卒中后抑郁患者失眠的疗效观察[J]. 健康必读,2021(11). 771. 闫改霞,白振军,宋雅琴,等. 邢氏针法联合黄连温胆汤加减治疗对痰热内扰型中风后失眠症睡眠、疲劳和生活质量的影响[J]. 中华中医药学刊,2021(10). DOI:10.13193/j.issn.1673-7717.2021.10.058. 772. 杨丽霞,彭志华,李慧莺,等. 通元针法治疗缺血性脑卒中后睡眠障碍的临床疗效研究[J]. 按摩与康复医学,2021(4). DOI:10.19787/j.issn.1008-1879.2021.04.029. 773. 杨倩,马付玉,王洁. 百乐眠胶囊联合重复经颅磁刺激治疗缺血性脑卒中继发失眠的临床疗效及其对多导睡眠图参数的影响[J]. 临床合理用药杂志,2021(15). DOI:10.15887/j.cnki.13-1389/r.2021.15.010. 774. 杨田野,王磊,孙雨桐. 益心安神汤联合调神针法治疗脑卒中后睡眠障碍[J]. 中医学报,2021(5). DOI:10.16368/j.issn.1674-8999.2021.05.232. 775. 杨艳平,王少敏（通讯作者）. 耳穴埋豆联合雷火灸治疗中风后遗症期失眠患者疗效观察[J]. 健康管理,2021(4). 776. Qiu X, Han NS, Yao JX, Yu FR, Lin YY, Zhuang X. Acupuncture Reduced the Risk for Insomnia in Stroke Patients: A Propensity-Score Matched Cohort Study. Front Aging Neurosci. 2021 Aug 13;13:698988. doi: 10.3389/fnagi.2021.698988. PMID: 34483881; PMCID: PMC8414891. 777. Seo Y, Jin C, Jang BH, Jeon JP, Lee YS, Yang SB, Jung WS, Moon SK, Cho KH, Kwon S. Successful treatment of restless leg syndrome with the traditional herbal medicines Dangguijakyak-san and Shihogyeji-tang: A case report (CARE-compliant). Medicine (Baltimore). 2021 Aug 6;100(31):e26800. doi: 10.1097/MD.0000000000026800. PMID: 34397832; PMCID: PMC8341247. 778. [1]邵斌,黄凯烨,王建斌,陈捷.针刺对中风后睡眠-觉醒障碍患者日间功能的影响:随机对照试验（英文）[J].World Journal of Acupuncture-Moxibustion,2021,31(02):95-99. 779. Song Y, Wang X, Schubert F. Application of Wireless Dynamic Sleep Monitor in Acupuncture Treatment of Insomnia after Ischemic Stroke: A Retrospective Study. Evid Based Complement Alternat Med. 2021 Apr 1;2021:5524622. doi: 10.1155/2021/5524622. PMID: 33868435; PMCID: PMC8035007. 780. Yang J. Acupuncture treatment for post-stroke insomnia: A systematic review and meta-analysis of randomized controlled trials. Complement Ther Clin Pract. 2021 Aug;44:101396. doi: 10.1016/j.ctcp.2021.101396. Epub 2021 Apr 23. PMID: 33957493. 781. Zhang P, Cheng L, Tian Q, Chen G, Chen C, Xu J. Effect of acupuncture on sleep quality and neurological function in stroke patients with sleep apnea syndrome. Am J Transl Res. 2021 May 15;13(5):5635-5640. PMID: 34150169; PMCID: PMC8205691. 782. 陈丽萍,韩棉梅,傅思媚. 电针联合重复经颅磁刺激治疗脑卒中后抑郁伴失眠的临床研究[J]. 广州医药,2021(2). DOI:10.3969/j.issn.1000-8535.2021.02.002. 783. 陈鑫成,沈俭,张路. 酸枣仁汤加味治疗老年脑卒中后认知障碍伴失眠的效果及对脑血流的影响[J]. 中国医学创新,2021(23). DOI:10.3969/j.issn.1674-4985.2021.23.025. 784. 陈苑婷,陈薇薇. 安神沐足方联合坎离砂外敷涌泉穴治疗中风后失眠的效果[J]. 中国卫生标准管理,2021(12). DOI:10.3969/j.issn.1674-9316.2021.12.042. 785. 陈赟,王鑫栋,张闻东,等. 艾灸治疗卒中相关失眠28例临床观察[J]. 甘肃中医药大学学报,2021(2). DOI:10.16841/j.issn1003-8450.2021.02.11. 786. 董致郅,段娜,李明真,等. "调阴阳、和营卫"法对中风后失眠病人睡眠质量的影响[J]. 中西医结合心脑血管病杂志,2021(13). DOI:10.12102/j.issn.1672-1349.2021.13.006. 787. 杜婷,赵惠. 中药熏洗方足浴干预中风后失眠的疗效观察及护理体会[J]. 中医外治杂志,2021(2). DOI:10.3969/j.issn.1006-978X.2021.02.038. 788. 范晓红. 脑梗死后遗症患者失眠的中医护理干预效果分析[J]. 母婴世界,2021(15). 789. 付美婷,刘娟. 开天门联合五行音乐疗法治疗脑卒中后失眠的疗效观察[J]. 中医临床研究,2021(2). DOI:10.3969/j.issn.1674-7860.2021.02.025. 790. 高毅东,黄俊山,尹鹭峰,等. 昼夜分治法治疗中风后失眠的随机对照研究[J]. 世界睡眠医学杂志,2021(4). DOI:10.3969/j.issn.2095-7130.2021.04.006. 791. 古艳湘,刘健红. 中药足浴治疗肝郁气滞型脑梗死后失眠临床研究[J]. 广州中医药大学学报,2021(4). DOI:10.13359/j.cnki.gzxbtcm.2021.04.020. 792. 郭健. 针刺联合艾灸引气归元穴组疗法在脑卒中后失眠患者中的应用效果[J]. 黑龙江医学,2021(3). DOI:10.3969/j.issn.1004-5775.2021.03.005. 793. 郭银凤. 耳穴埋豆配合放松训练对中风后失眠的护理效果研究[J]. 康颐,2021(9). DOI:10.12332/j.issn.2095-6525.2021.09.210. 794. 韩朋娜. 中医体质护理对脑卒中后遗症期失眠患者睡眠质量的疗效观察[J]. 世界最新医学信息文摘（连续型电子期刊）,2021(13). DOI:10.3969/j.issn.1671-3141.2021.13.143. 795. 何鑫,刘翠. 中西医结合护理对脑梗塞后遗症期失眠患者睡眠质量的影响研究[J]. 贵州医药,2021(3). DOI:10.3969/j.issn.1000-744X.2021.03.084. 796. 黄玉娴,唐旭丽,蒋颖,等. 中药药枕联合生理-行为睡眠护理干预在脑卒中后睡眠障碍患者中的应用[J]. 护理实践与研究,2021(6). DOI:10.3969/j.issn.1672-9676.2021.06.043. 797. 焦丽娜,刘志强,王玉华. 腕踝针治疗中风后失眠29例[J]. 光明中医,2021(8). DOI:10.3969/j.issn.1003-8914.2021.08.042. 798. 焦雪蕾,周艳朋,刘宏伟,等. 通督益脑安神方在提高气滞血瘀型卒中后失眠患者PSQI睡眠指数及生活质量的效果观察[J]. 四川中医,2021(8). 799. 金鑫,王娜娜,邹蕴,等. 杵针结合针灸疗法缓解中风患者睡眠障碍临床观察[J]. 辽宁中医药大学学报,2021(8). DOI:10.13194/j.issn.1673-842x.2021.08.029. 800. 兰花琴. 针刺治疗中风后失眠的效果观察[J]. 当代医药论丛,2021(10). DOI:10.3969/j.issn.2095-7629.2021.10.105. 801. [1]吴玉红,王松龄.王松龄教授治疗中风病失眠的临床观察[J/OL].辽宁中医杂志:1-8[2022-01-27].http://kns.cnki.net/kcms/detail/21.1128.R.20210427.1446.102.html. 802. 夏卉,李琴,钱红霞. 八段锦对脑卒中后失眠的效果分析[J]. 特别健康,2021(25). 803. 辛衍代. 自拟通脑清心汤治疗脑卒中后抑郁失眠的效果观察[J]. 医药前沿,2021(14). 804. 叶稳田,唐翠云,易珍. 黄连阿胶汤加减治疗脑卒中后失眠阴虚火旺证的疗效及复发率评价[J]. 当代医学,2021(24). DOI:10.3969/j.issn.1009-4393.2021.24.051. 805. [1]于小洁.针刺四关穴配合耳穴压豆治疗脑卒中后失眠的临床疗效[J].中国全科医学,2021,24(S1):175-176. 806. 袁强强. 对使用中医针灸治疗中风患者失眠症治疗结果进行分析[J]. 健康管理,2021(10). 807. 原亚静,商庆新. 针药结合治疗脑卒中后心肾不交型失眠的临床疗效[J]. 中西医结合心脑血管病杂志,2021(4). DOI:10.12102/j.issn.1672-1349.2021.04.038. 808. 张晓铃. 中西医联合治疗脑梗死并失眠在改善神经功能及睡眠质量方面的效果[J]. 世界睡眠医学杂志,2021(5). DOI:10.3969/j.issn.2095-7130.2021.05.027. 809. 赵冬芝,洪秋阳,陈东丽,等. 柴芍龙牡汤加味治疗肝郁血虚型脑卒中后失眠的疗效观察[J]. 中西医结合心脑血管病杂志,2021(8). DOI:10.12102/j.issn.1672-1349.2021.08.035. 810. 朱俊玲. 耳穴压豆治疗中风后失眠临床观察[J]. 东方药膳,2021(12). |
| --- |
