# Supplementary material for: Traditional Chinese Medicine for Post-stroke Sleep Disorders: The Evidence Mapping of Clinical Studies
Source: Front Psychiatry. 2022 Jun 15;13:865630. doi: 10.3389/fpsyt.2022.865630 (PMC9240765; doi:10.3389/fpsyt.2022.865630)
Supplement: Supplementary file 3 [file Table_3.DOCX]

**Table S3. The evidence composition in terms of intervention**

| **Types of intervention** | **Number of studies (n=810)** |
| --- | --- |
| **CHM (n=265)** | |
| Oral CHM formulae | 131 |
| Oral Chinese patent drug | 100 |
| CHM bathing therapy | 13 |
| CHM aromatherapy | 7 |
| Combination of multiple CHM therapies | 6 |
| CHM with oxygen inhalation | 4 |
| CHM preparation unspecified | 3 |
| CHM iontophoresis | 1 |
| **Acupuncture therapies (n=349)** | |
| Needling acupuncture | 194 |
| Combination of multiple acupuncture therapies | 65 |
| Auricular acupressure | 33 |
| Moxibustion | 16 |
| Acupoint application | 13 |
| Electro-acupuncture | 10 |
| Acupressure | 10 |
| Acupoint catgut embedding | 3 |
| Acupuncture therapies not specified | 2 |
| Acupoint injection | 1 |
| Acupoint bloodletting | 1 |
| Acupoint cupping | 1 |
| **Other TCM therapies (n=22)** | |
| Massage | 8 |
| Five-element musical therapy | 6 |
| Traditional physical exercises | 5 |
| Cupping | 1 |
| Skin scraping | 1 |
| Psychotherapy guided by TCM theory | 1 |
| **Combination of multiple TCM therapies (n=172)** | |
| **TCM therapies without details (n=2)** | |

**Notes:** CHM: Chinese herbal medicine; TCM: traditional Chinese medicine.
