# Supplementary material for: Traditional Chinese Medicine for Post-stroke Sleep Disorders: The Evidence Mapping of Clinical Studies
Source: Front Psychiatry. 2022 Jun 15;13:865630. doi: 10.3389/fpsyt.2022.865630 (PMC9240765; doi:10.3389/fpsyt.2022.865630)
Supplement: Supplementary file 4 [file Table_4.DOCX]

**Table S4 The full ingredients of Chinese herbal medicine prescriptions**

| **Name of prescriptions** | **Ingredients in Chinese Pinyin** | **Ingredients in English name** | **Species** |
| --- | --- | --- | --- |
| *Yangxueqingnao g*ranule (Chinese patent drug) | 1. *Danggui* 2. *Chuanxiong* 3. *Baishao* 4. *Shudihuang* 5. *Gouteng* 6. *Jixueteng* 7. *Xiakucao* 8. *Juemingzi* 9. *Zhenzhumu* 10. *Yanhusuo* | 1. ANGELICAE SINENSIS RADIX 2. CHUANXIONG RHIZOMA 3. PAEONIAE RADIX ALBA 4. REHMANNIAE RADIX PRAEPARATA 5. UNCARIAE RAMULUS CUM UNCIS 6. SPATHOLOBI CAULIS 7. PRUNELLAE SPICA 8. CASSIAE SEMEN 9. MARGARITIFERA CONCHA 10. CORYDALIS RHIZOMA | 1. dried root of *Angelica sinensis (Oliv.) Diels*, 2. dried rhizome of *Ligusticum chuanxiong Hort.*, 3. dried rhizome of *Paeonia lactiflora Pall*., 4. Processed products of Fresh or dried root tubers of *Rehmannia glutinosa Libosc*h., 5. dried hooked stem branches of *Uncaria rhynchophylla (Miq.) Miq. ex Havil., Uncaria macrophylla Wall., Uncaria hirsuta Havil., Uncaria sinensis (Oliv.) Havil. or Uncaria sessilifrudus Roxb.,* 6. dried stem of *Spatholobus suberectus Dunn*, 7. dried ear of *Prunella vulgaris L*., 8. dried ripe seed of *Cassia obtusifolia L. or Cassia tora L.,* 9. conch of *Hyriopsis cumingii (Lea) , Cristaria plicata (leach) , Pteria martensii (Dunker)*, 10. dried tuber of *Corydalis yanhusuo W.T.Wang* |
| *Bailemian* capsule (Chinese patent drug) | 1. *Baihe* 2. *Ciwujia* 3. *Shouwuteng* 4. *Hehuanhua* 5. *Zhenzhumu* 6. *Shigao* 7. *Suanzaoren* 8. *Fuling* 9. *Yuanzhi* 10. *Xuanshen* 11. *Dihuang* 12. *Maidong* 13. *Wuweizi* 14. *Dengxincao* 15. *Danshen* | 1. LILII BULBUS 2. ACANTHOPANACIS SENTICOSI RADIX ET RHIZOMA SEU CAULIS 3. POLYGONI MULTIFLORI CAULIS 4. ALBIZIAE FLOS 5. MARGARITIFERA CONCHA 6. GYPSUM FIBROSUM 7. ZIZIPHI SPINOSAE SEMEN 8. PORIA 9. POLYGALAE RADIX 10. SCROPHULARIAE RADIX 11. REHMANNIAE RADIX 12. OPHIOPOGONIS RADIX 13. SCHISANDRAE CHINENSIS FRUCTUS 14. JUNCI MEDULLA 15. SALVIAE MILTIORRHIZAE RADIX ET RHIZOMA | 1. dried fleshy scaly leaves of *Lilium lancifolium Thunb. Lilium brownii F.E.Brown var. viridulum Baker or Lilium pumilum DC.*, 2. dried roots and rhizomes or stems of *Acanthopanax senticosus (Rupr.etMaxim.) Harms*, 3. dried stem of *Polygonum multiflorum Thunb.*, 4. dried inflorescence or bud of *Albizia julibrissin Durazz.*, 5. shell of *Hyriopsis cumingii (Lea)*, *Cristaria plicata (leach) or Pteria martensii (Dunker)* , 6. CaSO4·2H2O, 7. dried ripe seed of *Ziziphus jujuba Mill. var. Spinosa (Bunge) Hu ex H. F. Chou*, 8. dried sclerotia of *Poria cocos (Schw.) Wolf*, 9. dried root of *Polygala tenuifolia Willd. or Polygala sibirica L.*, 10. dried root of *Scrophularia ningpoensis Hemsl.*, 11. Fresh or dried root tubers of *Rehmannia glutinosa Libosch.*, 12. dried root of *Ophiopogon japonicus (L.f) Ker-Gawl*., 13. dried ripe fruit of *Schisandra chinensis (Turcz.) Baill.*, 14. dried stem pith of *Juncus effusus L*., 15. dried roots and rhizomes of *Salvia miltiorrhiza Bge*. |
| *Suanzaoren* decoction (traditional formulae) | 1. *Suanzaoren* 2. *Gancao* 3. *Zhimu* 4. *Fuling* 5. *Chuanxiong* | 1. ZIZIPHI SPINOSAE SEMEN 2. GLYCYRRHIZAE RADIX ET RHIZOMA 3. ANEMARRHENAE RHIZOMA 4. PORIA 5. CHUANXIONG RHIZOMA | 1. dried ripe seed of *Ziziphus jujuba Mill. var. Spinosa (Bunge) Hu ex H. F. Chou*, 2. dried roots and rhizomes of *Glycyrrhiza uralensis Fisch.,Glycyrrhiza inflata Bat.or Glycyrrhiza glabra L.*, 3. dried rhizome of *Anemarrhena asphodeloides Bge*., 4. dried sclerotia of *Poria cocos (Schw.) Wolf*, 5. dried rhizome of *Ligusticum chuanxiong Hort*. |
| *Chaihulonggumuli* decoction (traditional formulae) | 1. Chaihu 2. Muli 3. Shengjiang 4. Renshen 5. Guizhi 6. Fuling 7. Banxia 8. Huangqin 9. Dahuang 10. Dazao | 1. BUPLEURI RADIX 2. OSTREAE CONCHA 3. ZINGIBERIS RHIZOMA RECENS 4. GINSENG RADIX ET RHIZOMA 5. CINNAMOMI RAMULUS 6. PORIA 7. PINELLIAE RHIZOMA 8. SCUTELLARIAE RADIX 9. RHEI RADIX ET RHIZOMA 10. JUJUBAE FRUCTUS | 1. dried root of *Bupleurum chinense DC. or Bupleurum scorzonerifolium Willd.*, 2. shell of *Ostrea gigas Thunberg, Ostrea talienwhanensis Crosse or Ostrea rivularis Gould*, 3. fresh rhizome of *Zingiber officinale Rosc.*, 4. dried roots and rhizomes of *Panax ginseng C. A. Mey.* , 5. dried twigs of *Cinnamomum cassia Presl*, 6. dried sclerotia of *Poria cocos (Schw.) Wolf*, 7. dried tuber of *Pinellia ternata (Thunb.) Breit.*, 8. dried root of *Scutellaria baicalensis Georgi*, 9. dried roots and rhizomes of *Rheum palmatum L.*, *Rheum tanguticum Maxim. ex Bal£. or Rheum officinale Bail1*, 10. dried ripe fruit of *Ziziphus jujuba Mill*. |
