# Supplementary material for: Traditional Chinese Medicine for Post-stroke Sleep Disorders: The Evidence Mapping of Clinical Studies
Source: Front Psychiatry. 2022 Jun 15;13:865630. doi: 10.3389/fpsyt.2022.865630 (PMC9240765; doi:10.3389/fpsyt.2022.865630)
Supplement: Supplementary file 5 [file Table_5.DOCX]

**Table S5. Quality assessment of systematic review**

| **Study** | **1** | **2** | **3** | **4** | **5** | **6** | **7** | **8** | **9** | **10** | **11** | **12** | **13** | **14** | **15** | **16** | **Ranking of quality** |
| --- | --- | --- | --- | --- | --- | --- | --- | --- | --- | --- | --- | --- | --- | --- | --- | --- | --- |
| Chai B 2019 | Y | N | N | N | Y | Y | N | PY | PY | N | N | N | N | N | N | N | Very low level |
| Fu M 2018 | Y | N | N | PY | Y | N | N | N | PY | N | Y | N | Y | Y | N | N | Very low level |
| Lee SH 2016 | Y | N | N | PY | N | Y | N | Y | Y | N | N | N | Y | Y | Y | N | Very low level |
| Li Y 2018 | Y | N | N | PY | N | N | N | N | PY | N | N | N | Y | N | N | N | Very low level |
| Liang X 2015 | N | N | N | PY | N | N | N | N | N | N | N | N | N | Y | N | N | Very low level |
| Liu X 2019 | Y | N | N | PY | N | N | N | N | N | N | NA | NA | N | N | N | N | Very low level |
| Nie F 2020 | Y | N | N | PY | Y | Y | N | PY | Y | N | N | N | Y | Y | Y | N | Very low level |
| Zhang S 2019a | Y | N | N | PY | Y | Y | N | N | Y | N | Y | N | Y | Y | Y | N | Very low level |
| Yang J 2021 | Y | N | N | PY | N | N | N | PY | PY | N | Y | N | Y | Y | N | Y | Very low level |
| Yu L 2020 | Y | N | N | PY | Y | N | N | PY | PY | N | Y | N | Y | Y | N | N | Very low level |
| Zeng L 2015 | Y | N | N | Y | Y | Y | N | PY | Y | N | Y | Y | Y | N | N | N | Very low level |
| Zhang Q 2020 | Y | N | N | PY | Y | N | N | N | PY | N | N | Y | Y | Y | N | N | Very low level |
| Zhang S 2019b | Y | N | N | PY | Y | N | N | N | Y | N | Y | N | Y | Y | Y | N | Very low level |
| Zhang S 2020 | Y | N | N | PY | Y | N | N | N | Y | N | Y | N | Y | N | N | N | Very low level |

**Notes:** Abbreviations: NA for not applicable due to no meta conducted, Y for yes, PY for partial yes, N for no.

**AMSTAR 2 items:**

- item 1 for Did the research questions and inclusion criteria for the review include the components of PICO?,
- item 2 for Did the report of the review contain an explicit statement that the review methods were established prior to the conduct of the review and did the report justify any significant deviations from the protocol?,
- item 3 for Did the review authors explain their selection of the study designs for inclusion in the review? ,
- item 4 for Did the review authors use a comprehensive literature search strategy?,
- item 5 for Did the review authors perform study selection in duplicate?,
- item 6 for Did the review authors perform data extraction in duplicate?,
- item 7 for Did the review authors provide a list of excluded studies and justify the exclusions?,
- item 8 for Did the review authors describe the included studies in adequate detail?,
- item 9 for Did the review authors use a satisfactory technique for assessing the risk of bias (RoB) in individual studies that were included in the review? ,
- item 10 for Did the review authors report on the sources of funding for the studies included in the review?,
- item 11 for If meta-analysis was performed did the review authors use appropriate methods for statistical combination of results?,
- item 12 for If meta-analysis was performed, did the review authors assess the potential impact of RoB in individual studies on the results of the meta-analysis or other evidence synthesis?,
- item 13 for Did the review authors account for RoB in individual studies when interpreting/ discussing the results of the review?,
- item 14 for Did the review authors provide a satisfactory explanation for, and discussion of, any heterogeneity observed in the results of the review?,
- item 15 for If they performed quantitative synthesis did the review authors carry out an adequate investigation of publication bias (small study bias) and discuss its likely impact on the results of the review? ,
- item 16 for Did the review authors report any potential sources of conflict of interest, including any funding they received for conducting the review?.
