# Supplementary material for: Traditional Chinese Medicine for Post-stroke Sleep Disorders: The Evidence Mapping of Clinical Studies
Source: Front Psychiatry. 2022 Jun 15;13:865630. doi: 10.3389/fpsyt.2022.865630 (PMC9240765; doi:10.3389/fpsyt.2022.865630)
Supplement: Supplementary file 6 [file Table_6.DOCX]

**Supplementary Table 6**

**Pooled results of RCTs on TCM for PSSDs in details (n=632 RCTs)**

| Insomnia | | | | | | | | | | | | |
| --- | --- | --- | --- | --- | --- | --- | --- | --- | --- | --- | --- | --- |
| Outcome | Chinese herbal medicine (n=125) | | | Acupuncture (n=195) | | | Other TCM therapies (n=8) | | | Combination therapies (n=94) | | |
|  | Favoring TCM | No difference | Favoring comparator | Favoring TCM | No difference | Favoring comparator | Favoring TCM | No difference | Favoring comparator | Favoring TCM | No difference | Favoring comparator |
| Sleep quality | 81 | 7 | 0 | 144 | 13 | 0 | 4 | 2 | 0 | 74 | 2 | 0 |
| Sleep quality | 7 | 2 | 0 | 19 | 5 | 0 | 0 | 0 | 0 | 13 | 1 | 0 |
| Global effectiveness | 88 | 12 | 0 | 123 | 30 | 1 | 4 | 2 | 0 | 64 | 5 | 0 |
| Recurrent insomnia | 0 | 0 | 0 | 0 | 0 | 0 | 0 | 0 | 0 | 1 | 0 | 0 |
| Neurological scales | 10 | 3 | 0 | 16 | 2 | 0 | 0 | 1 | 0 | 6 | 4 | 0 |
| Cognition | 3 | 0 | 0 | 3 | 0 | 0 | 0 | 0 | 0 | 1 | 0 | 0 |
| Movement | 1 | 0 | 0 | 0 | 0 | 0 | 0 | 1 | 0 | 3 | 0 | 0 |
| QoL | 7 | 0 | 0 | 14 | 0 | 0 | 0 | 1 | 0 | 10 | 1 | 0 |
| ADL | 7 | 1 | 0 | 10 | 3 | 0 | 0 | 0 | 0 | 5 | 4 | 0 |
| Emotion | 21 | 3 | 0 | 33 | 5 | 0 | 1 | 0 | 0 | 14 | 0 | 0 |
| TCM syndromes | 12 | 0 | 0 | 8 | 3 | 0 | 0 | 0 | 0 | 10 | 1 | 0 |
| Laboratory test | 11 | 3 | 0 | 17 | 8 | 0 | 1 | 1 | 0 | 10 | 0 | 0 |
| Sleep related breathing disorders | | | | | | | | | | | | |
| Outcome | Chinese herbal medicine (n=16) | | | Acupuncture (n=9) | | | Other TCM therapies (n=0) | | | Combination therapies (n=1) | | |
|  | Favoring TCM | No difference | Favoring comparator | Favoring TCM | No difference | Favoring comparator | Favoring TCM | No difference | Favoring comparator | Favoring TCM | No difference | Favoring comparator |
| Sleep quality | 4 | 0 | 0 | 5 | 0 | 0 | 0 | 0 | 0 | 1 | 0 | 0 |
| Sleep quantity | 10 | 3 | 0 | 7 | 0 | 0 | 0 | 0 | 0 | 1 | 0 | 0 |
| Global effectiveness | 10 | 0 | 0 | 6 | 0 | 0 | 0 | 0 | 0 | 0 | 0 | 0 |
| Neurological scales | 9 | 1 | 0 | 0 | 0 | 0 | 0 | 0 | 0 | 0 | 0 | 0 |
| Cognition | 4 | 0 | 0 | 1 | 0 | 0 | 0 | 0 | 0 | 0 | 0 | 0 |
| Movements | 1 | 0 | 0 | 0 | 0 | 0 | 0 | 0 | 0 | 0 | 0 | 0 |
| Dysphasia | 1 | 0 | 0 | 0 | 0 | 0 | 0 | 0 | 0 | 0 | 0 | 0 |
| QoL | 1 | 1 | 0 | 0 | 0 | 0 | 0 | 0 | 0 | 0 | 0 | 0 |
| ADL | 3 | 0 | 0 | 0 | 0 | 0 | 0 | 0 | 0 | 0 | 0 | 0 |
| Recurrent stroke | 1 | 0 | 0 | 0 | 0 | 0 | 0 | 0 | 0 | 0 | 0 | 0 |
| Mortality | 0 | 1 | 0 | 0 | 0 | 0 | 0 | 0 | 0 | 0 | 0 | 0 |
| Anatomic index | 1 | 0 | 0 | 1 | 0 | 0 | 0 | 0 | 0 | 0 | 0 | 0 |
| TCM syndrome | 4 | 0 | 0 | 0 | 0 | 0 | 0 | 0 | 0 | 0 | 0 | 0 |
| Laboratory test | 7 | 1 | 0 | 3 | 1 | 0 | 0 | 0 | 0 | 0 | 0 | 0 |
| Other types of sleep disorders | | | | | | | | | | | | |
| Outcome | Chinese herbal medicine (n=3) | | | Acupuncture (n=15) | | | Other TCM therapies (n=2) | | | Combination therapies (n=0) | | |
|  | Favoring TCM | No difference | Favoring comparator | Favoring TCM | No difference | Favoring comparator | Favoring TCM | No difference | Favoring comparator | Favoring TCM | No difference | Favoring comparator |
| Sleep quality | 1 | 1 | 0 | 11 | 1 | 0 | 2 | 0 | 0 | 0 | 0 | 0 |
| Sleep quantity | 0 | 1 | 0 | 4 | 0 | 0 | 1 | 1 | 0 | 0 | 0 | 0 |
| Global effectiveness | 1 | 1 | 0 | 8 | 2 | 0 | 1 | 0 | 0 | 0 | 0 | 0 |
| Neurological scales | 0 | 1 | 0 | 2 | 1 | 0 | 0 | 0 | 0 | 0 | 0 | 0 |
| Movement | 0 | 0 | 0 | 1 | 0 | 0 | 0 | 0 | 0 | 0 | 0 | 0 |
| Emotion | 0 | 0 | 0 | 1 | 1 | 0 | 0 | 0 | 0 | 0 | 0 | 0 |
| ADL | 0 | 1 | 0 | 1 | 0 | 0 | 0 | 0 | 0 | 0 | 0 | 0 |
| No specific type of sleep disorders | | | | | | | | | | | | |
| Outcome | Chinese herbal medicine (n=62) | | | Acupuncture (n=59) | | | Other TCM therapies (n=8) | | | Combination therapies (n=35) | | |
|  | Favoring TCM | No difference | Favoring comparator | Favoring TCM | No difference | Favoring comparator | Favoring TCM | No difference | Favoring comparator | Favoring TCM | No difference | Favoring comparator |
| Sleep quality | 52 | 1 | 0 | 53 | 4 | 0 | 7 | 1 | 0 | 35 | 0 | 0 |
| Sleep quantity | 8 | 0 | 0 | 4 | 1 | 0 | 0 | 0 | 0 | 1 | 0 | 0 |
| Global effectiveness | 37 | 1 | 0 | 35 | 4 | 0 | 5 | 0 | 0 | 15 | 1 | 0 |
| Neurological scales | 23 | 2 | 0 | 11 | 2 | 0 | 0 | 0 | 0 | 6 | 1 | 0 |
| Cognition | 1 | 0 | 0 | 0 | 0 | 0 | 1 | 0 | 0 | 1 | 0 | 0 |
| Movement | 0 | 1 | 0 | 0 | 0 | 0 | 0 | 0 | 0 | 3 | 0 | 0 |
| QoL | 1 | 0 | 0 | 2 | 0 | 0 | 2 | 0 | 0 | 10 | 1 | 0 |
| ADL | 4 | 1 | 0 | 1 | 2 | 0 | 0 | 0 | 0 | 4 | 0 | 0 |
| Emotion | 18 | 0 | 0 | 6 | 1 | 0 | 2 | 0 | 0 | 6 | 0 | 0 |
| Recurrent stroke | 0 | 0 | 0 | 0 | 0 | 0 | 0 | 0 | 0 | 1 | 0 | 0 |
| TCM syndromes | 7 | 1 | 0 | 3 | 0 | 0 | 1 | 0 | 0 | 2 | 0 | 0 |
| Laboratory test | 13 | 0 | 0 | 7 | 0 | 0 | 0 | 0 | 0 | 2 | 0 | 0 |

**Notes:** RCTs: PSSDs: post-stroke sleep disorders; randomized controlled trials; TCM: traditional Chinese medicine. The results are presented with the number of studies.
